# Calcitonin Receptor Neurons in the Mouse Nucleus Tractus Solitarius Control Energy Balance via the Non-aversive Suppression of Feeding

**DOI:** 10.1016/j.cmet.2019.12.012

**Published:** 2020-02-04

**Authors:** Wenwen Cheng, Ian Gonzalez, Warren Pan, Anthony H. Tsang, Jessica Adams, Ermelinda Ndoka, Desiree Gordian, Basma Khoury, Karen Roelofs, Simon S. Evers, Andrew MacKinnon, Shuangcheng Wu, Henriette Frikke-Schmidt, Jonathan N. Flak, James L. Trevaskis, Christopher J. Rhodes, So-ichiro Fukada, Randy J. Seeley, Darleen A. Sandoval, David P. Olson, Clemence Blouet, Martin G. Myers

**Affiliations:** 1 Department of Internal Medicine, University of Michigan, Ann Arbor, MI 48105, USA; 2 Division of Endocrinology, Department of Pediatrics and Communicable Diseases, University of Michigan, Ann Arbor, MI 48105, USA; 3 Department of Molecular and Integrative Physiology, University of Michigan, Ann Arbor, MI 48105, USA; 4 Graduate Program in Cellular and Molecular Biology, University of Michigan, Ann Arbor, MI 48105, USA; 5 MRC Metabolic Diseases Unit, University of Cambridge Metabolic Research Laboratories, WT-MRC Institute of Metabolic Science, University of Cambridge, Cambridge CB2 0QQ, UK; 6 Department of Surgery, University of Michigan, Ann Arbor, MI 48105, USA; 7 Cardiovascular, Renal and Metabolic Diseases, AstraZenica LLC, Gaithersburg, MD 20878, USA; 8 Laboratory of Molecular and Cellular Physiology, Osaka University, Osaka 565-0871, Japan

**Keywords:** NTS, PBN, calcitonin receptor, aversion, anorexia, obesity

## Abstract

To understand hindbrain pathways involved in the control of food intake, we examined roles for calcitonin receptor (CALCR)-containing neurons in the NTS. Ablation of NTS *Calcr* abrogated the long-term suppression of food intake, but not aversive responses, by CALCR agonists. Similarly, activating Calcr^NTS^ neurons decreased food intake and body weight but (unlike neighboring Cck^NTS^ cells) failed to promote aversion, revealing that Calcr^NTS^ neurons mediate a non-aversive suppression of food intake. While both Calcr^NTS^ and Cck^NTS^ neurons decreased feeding via projections to the PBN, Cck^NTS^ cells activated aversive CGRP^PBN^ cells while Calcr^NTS^ cells activated distinct non-CGRP PBN cells. Hence, Calcr^NTS^ cells suppress feeding via non-aversive, non-CGRP PBN targets. Additionally, silencing Calcr^NTS^ cells blunted food intake suppression by gut peptides and nutrients, increasing food intake and promoting obesity. Hence, Calcr^NTS^ neurons define a hindbrain system that participates in physiological energy balance and suppresses food intake without activating aversive systems.

## Highlights

• NTS *Calcr* mediates food intake suppression but not aversive responses to sCT
• Activating NTS Calcr neurons non-aversively suppresses feeding
• These neurons act via non-CGRP PBN neurons
• These neurons control long-term energy balance, not just short-term feeding

## Graphical Abstract

**Figure:**
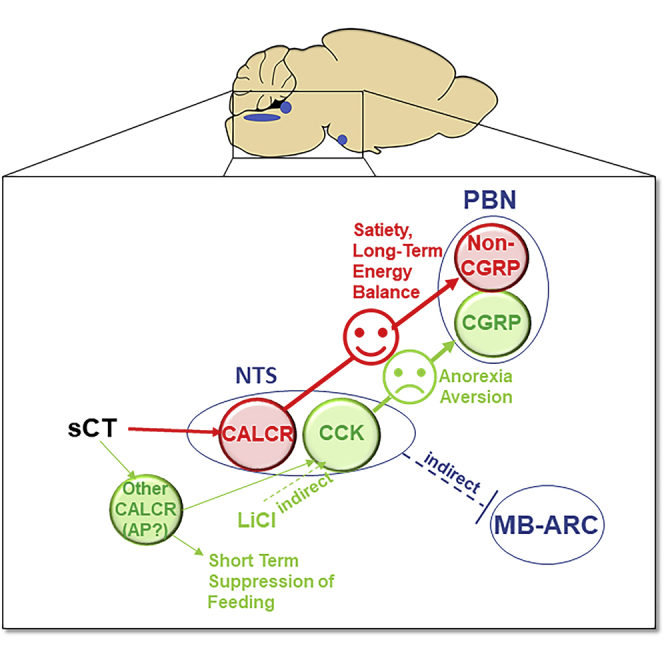

## Teaser

While the hindbrain is often postulated to control only short-term parameters of feeding via circuits that mediate aversive responses when activated strongly, Cheng et al. have identified a hindbrain system that participates in the physiological control of energy balance and suppresses food intake without activating aversive systems or symptoms.

## Context and Significance

**The most primitive portion of the brain, known as the hindbrain, has circuits that are widely thought to mediate only the short-term suppression of feeding by gut signals under normal physiologic conditions. These hindbrain circuits have been generally thought to suppress feeding through nausea and other aversive responses when activated strongly, as by drugs that mimic gut peptides. However, researchers at the University of Michigan identified a hindbrain system that not only participates in the physiological control of long-term energy balance, but also strongly and durably suppresses food intake without activating aversive systems or symptoms. In addition to revising our concept of food intake control by the hindbrain, these findings identify a circuit that represents a potentially ideal target for the treatment of obesity.**

## Introduction

Obesity affects over one-third of the adult population in developed countries, leading to diabetes, cardiovascular disease, and other conditions that cause substantial morbidity and mortality (https://www.cdc.gov/obesity/data/adult.html). Unfortunately, most current medical therapies are ineffective in treating obesity (Vasanth Rao et al., 2019). Agents that mimic the action of gut peptides (such as agonists [including calcitonin and amylin] for the calcitonin receptor [CALCR] and glucagon-like peptide-1 receptor [GLP1R]) suppress long-term food intake and promote significant weight loss, though (Adams et al., 2018, Mietlicki-Baase et al., 2013, Secher et al., 2014, Lutz et al., 1995, Yamaguchi et al., 2015, Fujioka, 2015, Ladenheim, 2015). Thus, it is important to understand the neural mechanisms by which such peptides mediate their anorexic effects.

*Calcr* is widely distributed in the brain, including in several areas linked to food intake, including the hypothalamic arcuate nucleus (ARC), the paraventricular hypothalamic nucleus (PVH), and the amygdala (Becskei et al., 2004). The nucleus tractus solitarius (NTS; a CNS region crucial for integrating gut-derived prandial signals and promoting meal termination) (Beutler et al., 2017, Blouet and Schwartz, 2012, D’Agostino et al., 2016, Grill and Hayes, 2009, Grill and Hayes, 2012, Hayes et al., 2011, Hayes et al., 2016a, Hayes et al., 2010, Hayes et al., 2016b) also contains *Calcr* cells (Calcr^NTS^ neurons). Importantly, while agonists for CALCR (and other gut peptide receptors) decrease food intake, they also produce aversive responses that mimic gut malaise (Adams et al., 2018, Halatchev and Cone, 2005, Schier et al., 2012, Verbaeys et al., 2008), potentially limiting their therapeutic utility.

One line of thinking holds that meal-terminating brainstem circuits must also mediate aversive responses, such that overfeeding (and other gut-malaise-associated stimuli) would promote aversive signals by more strongly activating these circuits than would a normal-sized meal. Indeed, previous work has demonstrated that calcitonin gene-related peptide (CGRP)-containing neurons of the parabrachial nucleus (PBN; CGRP^PBN^ cells) mediate aversion (as well as anorexia) in response to a variety of cues of gastrointestinal malaise (including that induced by intraperitoneal LiCl) and that silencing CGRP^PBN^ cells increases meal size (Campos et al., 2016, Carter et al., 2015, Carter et al., 2013). For instance, stimulating PBN projections from *Cck*-expressing NTS (Cck^NTS^) neurons activates CGRP^PBN^ cells and promotes aversion and anorexia (Roman et al., 2017).

Normal food ingestion (or nutrient infusion into the gut) mediates positive reinforcement even while stimulating meal termination (Sclafani, 2013, Sclafani and Ackroff, 2012). Therefore, the circuits that terminate normal feeding must differ at least in part from those that convey aversive signals in response to gut malaise, and it should be possible to identify populations of NTS neurons that suppress food intake without activating CGRP^PBN^ neurons or causing aversion.

While gut-peptide-responsive cells in the NTS (and in the adjacent area postrema [AP]) play prominent roles in meal termination and the control of meal size (Grill and Hayes, 2012), the gut peptide receptors on these cells only modestly (if at all) impact the long-term physiologic control of food intake and energy balance (Blouet and Schwartz, 2012). Hence, conventional wisdom holds that while NTS cells play important roles in the suppression of feeding in response to gut peptide receptor agonism and the control of meal termination, these NTS neurons do not participate in the physiologic regulation of long-term energy balance. This idea has not been tested directly by interfering with the overall function of NTS neurons, though. Thus, in addition to understanding the roles for NTS cells in the anorectic response to pharmacologic CALCR agonism and defining hindbrain systems that mediate the non-aversive suppression of food intake, it will be important to directly determine roles for NTS cells in the physiologic control of food intake and energy homeostasis. Here, we examined roles for Calcr^NTS^ cells, demonstrating their role in mediating the non-aversive suppression of food intake and revealing their importance for the long-term control of energy balance.

## Results

### Attenuation of the Anorectic, but Not Aversive, Response to CALCR Agonism in Calcr^NTS^KO Mice

**Figure 1:**
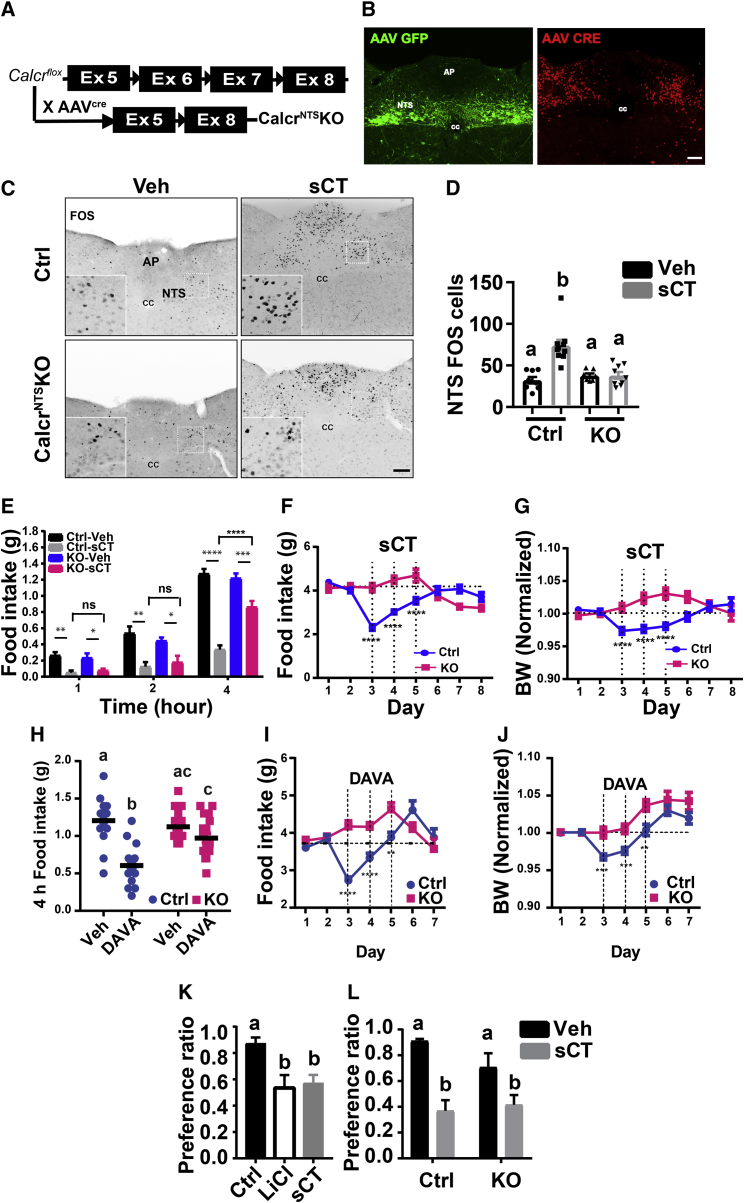
Deletion of *Calcr* in Calcr^NTS^ Neurons Prevents Food Intake Suppression but Not CTA Formation to sCT Treatment (A) Schematic diagram showing intra-NTS AAV^cre^ delivery in *Calcr*^*flox*^ mice to generate Calcr^NTS^KO mice. (B) Representative images show AAV^GFP^ (GFP, green, left panel) and AAV^cre^ (mCherry, red, right panel) expression in the NTS of control and Calcr^NTS^KO mice. Scale bar, 150 μm. (C) Representative images showing FOS-IR (black) in vehicle- (Veh) and sCT- (IP, 150 μg/kg, 2 h) injected control and Calcr^NTS^KO mice. Scale bar, 150 μm; cc, central canal. (D) Quantification of FOS-IR cells in the NTS of mice treated as in (C). Shown is mean ± SEM; n = 4–10 per group. (E) Control and Calcr^NTS^KO mice were treated with Veh or sCT (150 μg/kg, IP) and food intake was measured for the subsequent 4 h. n = 8 in 1-h and 2-h groups, n = 26 in 4-h groups. (F and G) Daily food intake (F) and body weight (G) were measured during 2 days of vehicle, 3 days of sCT (IP, 150 μg/kg, BID), and 3 additional days of vehicle injection. Food intake and body weight were normalized to baseline. n = 18 in control group, n = 8–10 in Calcr^NTS^KO group. (H–J) Control (Ctrl, blue) and Calcr^NTS^KO (KO, red) mice were treated with Davalintide (560 μg/kg, IP, twice daily) and food intake was measured for the first 4 h of the dark cycle (H) or food intake (I) and body weight (J) were measured daily over 3 days of injections (dashed lines); n = 18 per group. (K and L) Conditioned taste aversion (CTA) was determined following the IP injection of Veh, LiCl (126 mg/kg), or sCT (150 μg/kg) in control (K) or control and Calcr^NTS^KO mice (L); n = 7–10 per group. All graphs: Mean ± SEM is shown. One-way ANOVA, Tukey’s multiple comparisons was performed in (D), (H), (K), and (L), different letters indicate differences between groups, p < 0.05. The same test was performed for each time point in (E); ns, not significant. Two-way ANOVA, Sidak’s multiple comparisons test was performed for (F, G, I, and J). ^∗^p < 0.05, ^∗∗^p < 0.01, ^∗∗∗^p < 0.001, ^∗∗∗∗^p < 0.0001.

We sought to understand the roles for specific *Calcr*-expressing neurons for the control of food intake. Based upon *Calcr* expression patterns in the hypothalamus, we crossed *Calcr*^*flox*^ (Yamaguchi et al., 2015) onto the *Sim1*^*cre*^ (Balthasar et al., 2005) or *Lepr*^*cre*^ (Patterson et al., 2011) backgrounds (Calcr^Sim1^KO [knockout] and Calcr^LepRb^KO mice, respectively) (Figure S1A) to ablate *Calcr* in neurons of the PVH and in leptin receptor (LepRb) neurons (primarily neuropeptide Y-, agouti-related peptide-, and gamma-aminobutyric-acid-containing (NAG) cells of the ARC) (Pan et al., 2018). We also injected AAV^cre−mCherry^ or AAV^GFP^ (control) into the NTS of *Calcr*^*flox/flox*^ mice to ablate *Calcr* expression in this brain region (Calcr^NTS^KO mice) (Figure 1A). We examined AAV reporter expression in the brains of all injected animals following the completion of experiments to ensure correct targeting (Figure 1B).

As expected, Calcr^NTS^KO mice exhibited reduced salmon calcitonin (sCT)-stimulated NTS FOS-immunoreactivity (IR) (Figures 1C, 1D, and S1F). In contrast, Calcr^LepRb^KO mice exhibited unchanged sCT-stimulated FOS-IR in the ARC (Figures S1B and S1C), and we detected increased sCT-stimulated FOS in the PVH of Calcr^Sim1^KO mice despite decreased PVH *Calcr* expression in Calcr^Sim1^KO mice (Figures S1D and S1E). These findings suggest that most sCT-stimulated FOS in the ARC and PVH is mediated indirectly (by other CALCR cells) and that PVH CALCR may suppress the sCT-dependent activation of PVH neurons by other CALCR-expressing cells.

Our analysis revealed no alteration in food intake, body weight, or body composition in Calcr^Sim1^KO, Calcr^LepRb^KO, and Calcr^NTS^KO mice (Figures S2A–S2I). Hence, *Calcr* expression in the PVH, the NTS, and in ARC LepRb neurons does not meaningfully contribute to long-term energy balance under normal conditions.

To determine the anorectic response of these KO animals to CALCR agonists, we examined feeding following sCT administration. We found that sCT decreased food intake by >40% during the first 4 h of the dark cycle and that 3 days of twice-daily sCT administration suppressed food intake and lowered body weight similarly in Calcr^Sim1^KO and Calcr^LepRb^KO mice and their controls (Figures S2J–S2O). Thus, *Calcr* expression in PVH Sim1 neurons and hypothalamic LepRb neurons is not required for the suppression of food intake by sCT.

While we found that sCT suppressed food intake normally in Calcr^NTS^KO mice for the first 2 h at the onset of the dark cycle, the anorectic effect of sCT in Calcr^NTS^KO mice attenuated by 4 h, and sCT failed to suppress 24 h food intake and reduce body weight during 3 days of sCT administration in these animals (Figures 1E–1G). Indeed, 3-day treatment with sCT promoted body weight gain (p = 0.047) in the Calcr^NTS^KO mice. Thus, not only are NTS *Calcr* (Calcr^NTS^) neurons crucial for the anorectic response to sCT, but an unidentified set of *Calcr* neurons elsewhere in the brain may mediate an orexigenic signal that is normally overcome by the anorexic signal from the Calcr^NTS^ cells.

In contrast to the reduced efficacy of sCT and davalintide (a co-agonist for the CALCR and amylin receptor [Mack et al., 2010]) on food intake suppression in Calcr^NTS^KO mice (Figures 1E–1J), Calcr^NTS^KO and control mice responded similarly to the NPY2R agonist, PYY3-36, and lipopolysaccharide (LPS) (Figures S2P–S2R), consistent with the specificity of NTS *Calcr* for the anorexic response to CALCR agonists. Overall, these data suggest that while NTS *Calcr* is not required for the control of energy balance at baseline, *Calcr* in the NTS mediates the long-term effects of CALCR agonists on food intake and body weight.

To understand the potential role for Calcr^NTS^ neurons in the aversive response to sCT, we utilized the conditioned taste aversion (CTA) assay, in which pairing of a stimulus with exposure to a novel tastant (e.g., grape flavor or 0.15% saccharine in drinking water, or high-fat diet [HFD]) inhibits the subsequent consumption of aversive stimulus-paired tastants. Like LiCl, sCT promotes a CTA in normal mice (Freed et al., 1981) (Figure 1K). Interestingly, although deletion of NTS *Calcr* abrogated the ability of sCT to reduce food intake (Figures 1E–1J), sCT treatment promoted a CTA in Calcr^NTS^KO mice similarly to control animals (Figure 1L), revealing that NTS *Calcr* is not required for the formation of a CTA to sCT, thus suggesting that Calcr^NTS^ cells might suppress food intake without participating in aversive responses.

### Calcr^NTS^ Neurons Non-aversively Decrease Food Intake and Body Weight

To understand the relationship of Calcr^NTS^ neurons to previously studied populations of NTS neurons, we examined the potential colocalization of Calcr^NTS^ cells with NTS neurons that express LepRb, cholecystokinin (Cck), or tyrosine hydroxylase (Th) (Figures S3A–S3D). While LepRb^NTS^ and Cck^NTS^ were distinct from Calcr^NTS^ cells, a significant proportion of Th^NTS^ cells contain *Calcr* (and vice versa); Th^NTS^ cells do not overlap significantly with Cck^NTS^ cells.

**Figure 2:**
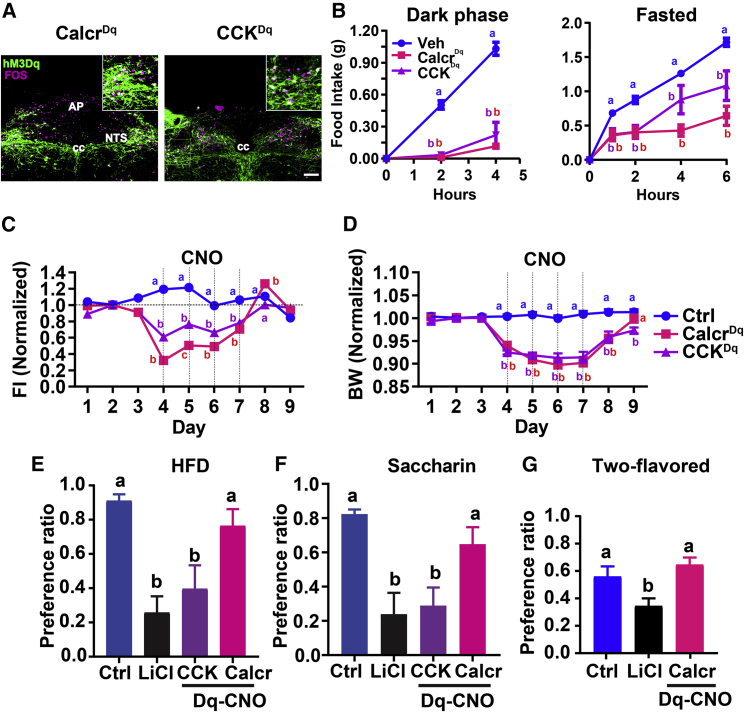
Effects of DREADD-Mediated NTS Neuron Activation on Food Intake and Body Weight, and CTA Induction (A) Representative images showing FOS (purple) and hM3Dq (green) 2 h after CNO injection (1 mg/kg, IP) in Calcr^NTS^-Dq (Calcr^Dq^) and Cck^NTS^-Dq (CCK^Dq^) mice. Scale bar, 150 μm; cc, central canal. (B) Food (chow) intake during CNO (1 mg/kg, IP) treatment of control (n = 23–25), Calcr^Dq^ (n = 7–20), and CCK^Dq^ (n = 5–9) mice during the first 4 h of the dark cycle (left panel) and the first 6 h following refeeding in overnight fasted mice (right panel). (C and D) Daily food intake (C) and body weight (D) were measured during 3 days of vehicle, 4 days of CNO (IP, 1 mg/kg, BID), and 2 additional days of vehicle injection. Food intake and body weight were normalized to baseline. Control: n = 12; Calcr^Dq^: n = 12; and CCK^Dq^: n = 7. (E–G) CTA was determined for control, Calcr^hm3dq^, and CCK^hm3dq^ groups in which stimulus was paired with HFD (E, n = 10, 10, 11, and 9 for control, LiCl, CCK^Dq^, and Calcr^Dq^ groups, respectively), Saccharin (F, n = 13, 11, 8, and 12 for control, LiCl, CCK^Dq^, and Calcr^Dq^ groups, respectively) or one of two flavors (G, n = 11, 12, and 8 for control, LiCl, and Calcr^Dq^ groups, respectively). All graphs: Mean ± SEM is shown. Two-way ANOVA, Sidak’s multiple comparisons test for (B), (C), and (D). One-way ANOVA, Tukey’s multiple comparisons was performed in (E), (F), and (G). Different letters indicate difference between groups, p < 0.05.

Because the chemogenetic activation of Cck^NTS^ cells has been shown to promote the aversive suppression of food intake (Roman et al., 2017), we utilized these cells as comparators for Calcr^NTS^ neurons (Figure 2). We bilaterally injected AAV^Flex−hM3Dq^ into the NTS of *Calcr*^*cre*^ or *Cck*^*cre*^ mice to cre-dependently express the Gq-coupled (activating; hM3Dq) designer receptor exclusively activated by designer drugs (DREADD) in Calcr^NTS^ and Cck^NTS^ cells, permitting their activation by the injection of clozapine-N-oxide (CNO) (Smith et al., 2016, Zhu and Roth, 2014). We used the post hoc detection of mCherry (which is fused to hM3Dq in AAV^Flex−hM3Dq^) in these Calcr^NTS^-Dq and Cck^NTS^-Dq mice to ensure that we analyzed only mice with NTS-restricted hM3Dq expression (Figure 2A).

CNO treatment promoted FOS-IR in mCherry-expressing cells in the NTS of Calcr^NTS^-Dq and Cck^NTS^-Dq mice, consistent with the DREADD-mediated activation of Calcr^NTS^ and Cck^NTS^ cells, respectively, in these animals (Figure 2A). The activation of Calcr^NTS^ or Cck^NTS^ cells at the onset of the dark cycle or following an overnight fast completely abrogated the intake of normal chow for 2 h and continued to dramatically reduce food intake for several hours (Figures S3E and S3F), although food intake suppression was less robust in Cck^NTS^-Dq mice than in Calcr^NTS^-Dq animals by 4 h after refeeding in fasted animals (Figure 2B). Furthermore, the twice-daily injection of CNO reduced food intake by 70% for the first 24 h and then by approximately 50% thereafter in Calcr^NTS^-Dq mice (Figure 2C). CNO reduced long-term food intake less dramatically in Cck^NTS^-Dq mice, but both mouse lines lost approximately 10% of their body weight over the 4-day treatment (Figures 2C and 2D). Thus, both Calcr^NTS^ and Cck^NTS^ cells suppress food intake and body weight, although Calcr^NTS^ cells mediate a greater long-term suppression of feeding than Cck^NTS^ cells.

Interestingly, despite the rapid and complete suppression of short-term food intake by both Calcr^NTS^ and Cck^NTS^ cells, only the activation of Cck^NTS^ cells promoted a CTA (Figures 2E–2G). Furthermore, in the two-flavor choice paradigm, animals consumed more of the flavor that was paired with the activation of Calcr^NTS^ cells (Figure S3G); the lack of significant change in the preference ratio likely reflects the lower sensitivity of this measure due to normalization for total volume consumed. Thus, not only is *Calcr* in NTS neurons crucial for the anorectic (but not aversive) response to sCT treatment, but the activation of Calcr^NTS^ cells causes the acute and chronic suppression of food intake while promoting reinforcement rather than aversion.

Because of the overlap between Calcr^NTS^ and Th^NTS^ cells, we also examined the response to activating Th^NTS^ cells (Figures S3H–S3L). While (like Calcr^NTS^ cells) we found that Th^NTS^ activation failed to promote a CTA, activating Th^NTS^ cells only weakly suppressed food intake compared to Calcr^NTS^ cells; thus, we continued to focus on Calcr^NTS^ neurons to understand NTS cells that mediate the non-aversive suppression of food intake. The more robust suppression of food intake by Calcr^NTS^ than Th^NTS^ cells might reflect some property of the non-Th Calcr^NTS^ cells that mediate a stronger anorectic response than Th^NTS^ cells.

**Figure 3:**
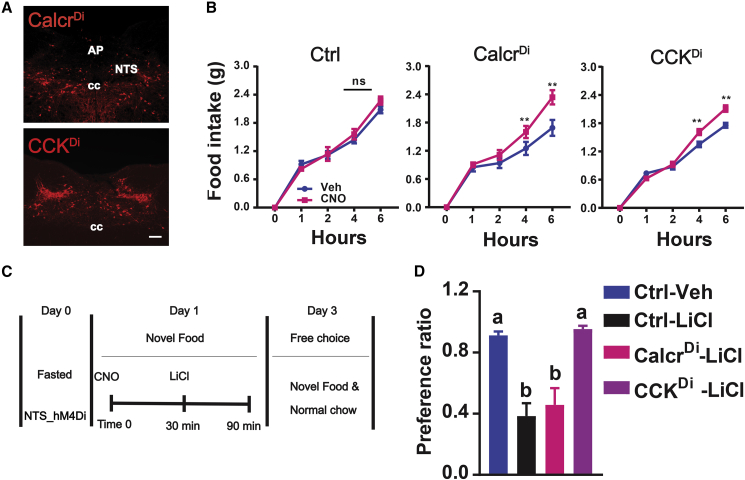
Distinct Roles for Calcr^NTS^ and Cck^NTS^ Neurons in CTA Formation (A) Representative images showing hM4Di (red) in Calcr^NTS^-Di (Calcr^Di^) and Cck^NTS^-Di (CCK^Di^) mice. Scale bar, 150 μm; cc, central canal. (B) Food intake for the first 6 h of refeeding following an overnight fast in the indicated groups of mice following treatment with vehicle (Veh) or CNO (IP, 1 mg/kg). Assay was performed using a crossover design with 1 week between conditions. Control: n = 5; Calcr^Di^ and CCK^Di^: n = 8 each. (C) Schematic diagram showing the protocol for testing CTA acquisition to novel food (HFD) by LiCl injection paired with CNO-mediated (IP, 1 mg/kg) silencing of Calcr^NTS^ or CCK^NTS^ neurons. (D) Quantification of CTA to HFD produced by LiCl injection paired with silencing Calcr^NTS^ or CCK^NTS^ neurons (n = 11 in vehicle and LiCl groups, n = 8 for in Calcr^Di^ and CCK^Di^ groups). Mean ± SEM is shown. Paired t test was performed for each time point in (B), ^∗∗^p < 0.01. One-way ANOVA, Tukey’s multiple comparisons was performed in (D), different letters indicate difference between conditions, p < 0.05.

Although the known role for the NTS in the control of food intake is consistent with the notion that Calcr^NTS^ and Cck^NTS^ cells suppress food intake directly, it is also possible that the activation of these cells could promote other effects (e.g., pain) that indirectly blunt feeding. We thus bilaterally injected AAV^Flex−hM4Di^ to express the Gi-coupled inhibitory hM4Di DREADD in Calcr^NTS^ and Cck^NTS^ cells (Calcr^NTS^-Di and Cck^NTS^-Di mice, respectively) (Figure 3A), permitting their acute inhibition by CNO. We found that CNO increased food intake following an overnight fast similarly in Calcr^NTS^-Di and Cck^NTS^-Di mice (Figure 3B). Furthermore, while the hM4Di-mediated inhibition of Calcr^NTS^ cells did not alter CTA formation in response to LiCl administration, CNO administration blocked CTA formation in Cck^NTS^-Di mice (Figures 3C and 3D). Thus, while Cck^NTS^ cells provoke aversive responses while suppressing food intake and are required for CTA formation in response to LiCl, Calcr^NTS^ cells similarly suppress food intake but are neither necessary nor sufficient to mediate a CTA.

### Downstream Targets of Calcr^NTS^ and Cck^NTS^ Neurons

**Figure 4:**
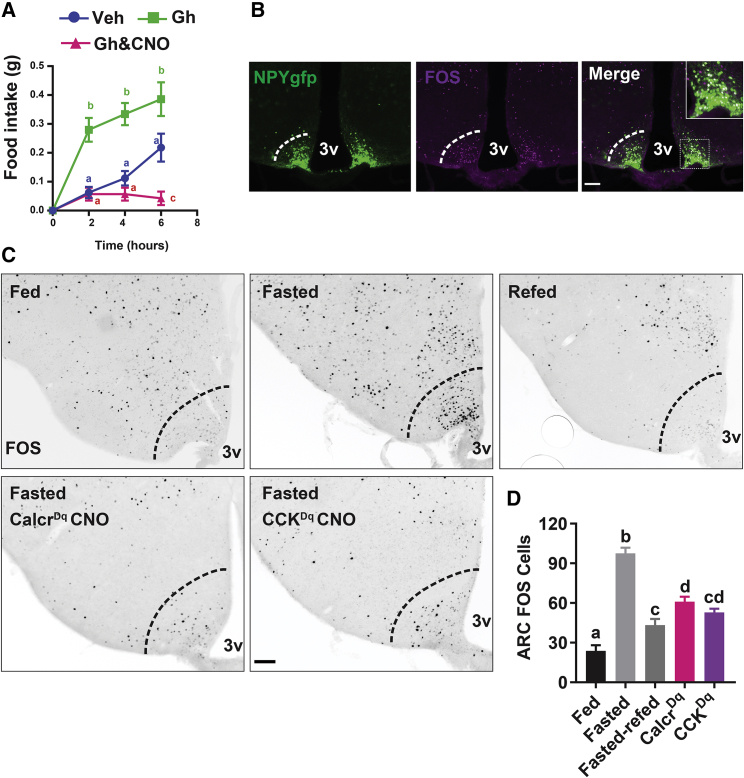
Inhibition of Fasting-Induced MB-ARC FOS during Calcr^NTS^ Neuron Activation (A) Calcr^NTS^-Dq mice were treated with vehicle (Veh), Ghrelin (Gh, 400 μg/kg, IP), or Gh plus CNO (IP, 1 mg/kg) 3 h after the onset of the light cycle (9 AM) and food intake was monitored over the subsequent 6 h. Veh: n = 14, Gh and Gh+CNO: n = 12. (B) Shown is a representative image of FOS-IR (purple) and GFP (green) in NPY^GFP^ brain sections from overnight-fasted mice. Scale bar, 150 μm; 3V, third cerebral ventricle. (C) Representative images showing ARC FOS (black) 3 h after the onset of the light cycle in *ad libitum*-fed (Fed), overnight fasted, overnight fasted and refed (Fast-refed), and overnight fasted with CNO (1 mg/kg, IP, 2 h prior to perfusion) in Calcr^Dq^ and CCK^Dq^ mice. Scale bar, 150 μm; 3V, third cerebral ventricle. Dashed line shows the limits of the MB-ARC region used for quantification. (D) Quantification of ARC FOS for the groups shown in (C); n = 5, 6, 4, 9, and 5, respectively. Mean ± SEM is shown. Two-way ANOVA, Sidak’s multiple comparisons test for (A). One-way ANOVA, Tukey’s multiple comparisons was performed in (D). Different letters indicate difference between conditions, p < 0.05.

Because Cck^NTS^ cells promote CTA formation while Calcr^NTS^ cells play no role in this process, these NTS cell types must act via different downstream circuits, at least in part. Nutritional signals from the gut suppress the activity of ARC NAG neurons (Beutler et al., 2017), presumably via a brainstem circuit. Because we found that the chemogenetic activation of Calcr^NTS^ cells suppressed ghrelin-simulated food intake (which is largely mediated by the activation of NAG neurons) (Figure 4A), we tested the potential role for Calcr^NTS^ cells in the inhibition of NAG cells. The fasting-induced activation of NAG cells can be monitored by the accumulation of FOS-IR in the medial basal ARC (MB-ARC; Figures 4B–4D) (Beutler et al., 2017, Olofsson et al., 2009); this fasting-induced MB-ARC activation can be decreased by refeeding. Similarly, the chemogenetic activation of Calcr^NTS^ cells suppressed fasting-induced MB-ARC FOS-IR (Figures 4C and 4D). Thus, Calcr^NTS^ cells inhibit fasting-induced MB-ARC FOS-IR cells, suggesting that hindbrain neurons control hypothalamic circuits crucial for the regulation of feeding. While the inhibition of MB-ARC cells may contribute to the suppression of food intake by Calcr^NTS^ neurons, the activation of Cck^NTS^ neurons also blunts fasting-induced MB-ARC FOS (Figures 4C and 4D). Hence, the difference in downstream signaling between Calcr^NTS^ and Cck^NTS^ cells must lie elsewhere.

**Figure 5:**
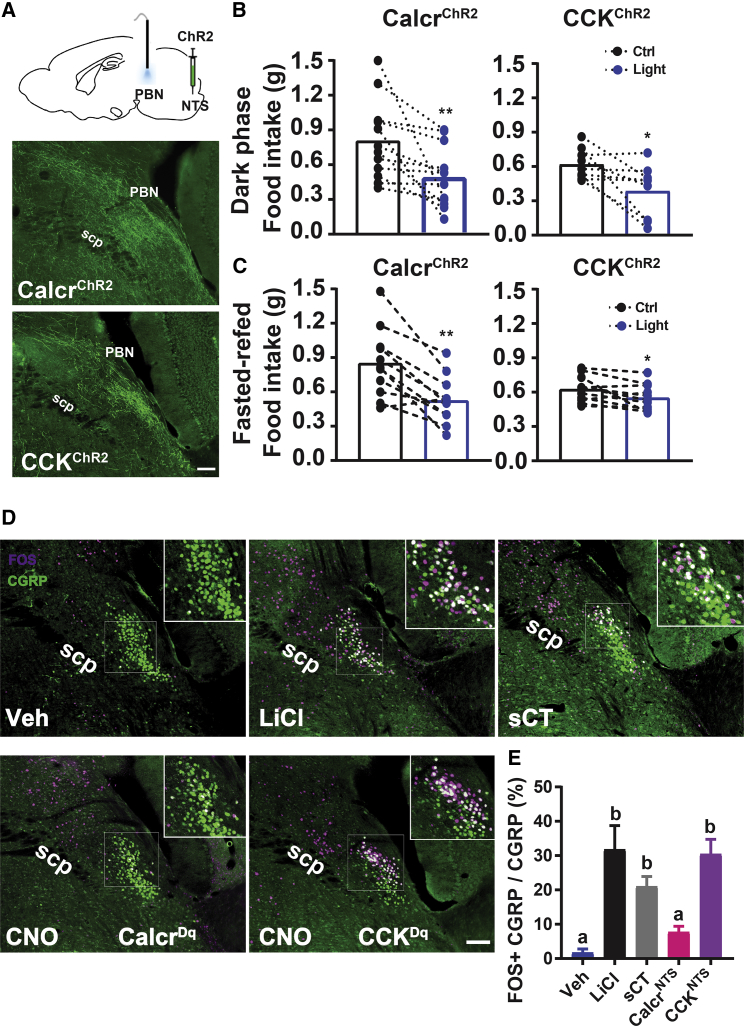
Roles for PBN Projections from Calcr^NTS^ and CCK^NTS^ Neurons (A) Diagram showing experimental setup and images showing ChR2-GFP-containing projections (green) into the PBN of Calcr^NTS^-ChR2 (Calcr^ChR2^) and Cck^NTS^-ChR2 (CCK^ChR2^) mice. Scale bar, 150 μm; scp, superior cerebellar peduncle. (B and C) Food intake over the first 2 h of the dark cycle (B) or during the first hour following the return of food to overnight-fasted mice (C) under control (Ctrl) or blue-light-stimulated (Light) conditions. Mean ± SEM is shown, paired t test was performed, ^∗^p < 0.05, ^∗∗^p < 0.01. Dark cycle: Calcr^ChR2^ n = 12, CCK^ChR2^ n = 9; refeeding: Calcr^ChR2^ n = 12, CCK^ChR2^ n = 11. (D) Representative images of FOS (purple) and CGRP cells (GFP, green) in the PBN of mice on the *Calca*^*cre−GFP*^ background 2 h after treatment with vehicle (Veh), sCT (150 μg/kg, IP), LiCl (126 mg/kg, IP), or CNO (1 mg/kg, IP) in Calcr^NTS^-Dq (Calcr^Dq^) and Cck^NTS^-Dq (CCK^Dq^) mice. Scale bar, 150 μm; scp, superior cerebellar peduncle. (E) Quantification of FOS-positive CGRP^PBN^ neurons from the groups in (D). Mean ± SEM is shown; n = 3 per group. One-way ANOVA, Tukey’s multiple comparisons was performed, different letters indicate differences, p < 0.05.

To define potential differences in the food-intake-suppressing systems engaged by Calcr^NTS^ and Cck^NTS^ neurons, we identified their downstream projections by the cre-dependent expression of a synaptophysin-mCherry (Syn-mCherry) fusion protein in these cells following the unilateral injection of AAV^Flex−Syn−mCherry^ into the NTS of *Calcr*^*cre*^ and *Cck*^*cre*^ animals. This analysis revealed projections to the PBN, PVH, and dorsomedial hypothalamic nucleus (DMH) from both Calcr^NTS^ and Cck^NTS^ neurons (Figures S4A–S4H). Similarly, activation of Calcr^NTS^ and Cck^NTS^ cells each increased FOS-IR in the NTS, PBN, PVH, and DMH, although the Calcr^NTS^ cells appeared to promote FOS-IR in the PVH and DMH more strongly than did Cck^NTS^ cells (Figures S4I–S4T). Interestingly, while the distribution of projections and Dq-stimulated FOS-IR within the target regions was similar in most cases, Calcr^NTS^ cells targeted a PBN region slightly more dorsal and medial to the region targeted by Cck^NTS^ cells (Figures S4B, S4F, S4J, S4N, and S4R). Despite this difference in PBN projection targets, the optogenetic activation of PBN terminals from either Calcr^NTS^ or Cck^NTS^ neurons suppressed food intake at the onset of the dark cycle and following an overnight fast (Figures 5A–5C).

Because CGRP^PBN^ cells contribute to the suppression of food intake during the activation of Cck^NTS^ neurons and mediate aversive responses (Carter et al., 2015, Carter et al., 2013, Roman et al., 2016), we examined the potential innervation and regulation of CGRP^PBN^ cells by Calcr^NTS^ cells. In our initial experiments, we stained for hM3Dq-mCherry and CGRP (which identifies the region containing CGRP^PBN^ cells but poorly reveals CGRP^PBN^ soma) in Calcr^NTS^-Dq and Cck^NTS^-Dq mice (Figure S5). While Cck^NTS^-Dq mice demonstrated substantial mCherry innervation of the CGRP-IR PBN field, Calcr^NTS^-Dq mice exhibited little overlap between the main PBN projection field of Calcr^NTS^ cells and CGRP^PBN^ cells. Furthermore, sCT promoted FOS-IR in the CGRP^PBN^ field in Calcr^NTS^KO and control mice (Figure S5). Thus, unlike Cck^NTS^ cells, it appears that Calcr^NTS^ cells neither innervate CGRP^PBN^ cells nor are required for the activation of CGRP^PBN^ cells by sCT.

To directly examine the ability of Calcr^NTS^ and Cck^NTS^ cells to activate CGRP^PBN^ cells, we bred *Calcr*^*cre*^ and *Cck*^*cre*^ onto the *Calca*^*cre−GFP*^ background (Carter et al., 2015), which permits the GFP-dependent detection of CGRP^PBN^ cells. We injected AAV^Flex−hM3Dq^ into the NTS of these mice and examined the distribution of innervation and FOS-IR within the PBN and its colocalization with GFP-labeled CGRP^PBN^ cells (Figures 5D, 5E, and S5). This analysis confirmed the more dorsal medial distribution of projections and FOS-IR within the PBN following the activation of Calcr^NTS^ neurons than for Cck^NTS^ cells. Furthermore, although Cck^NTS^ cells promoted strong FOS accumulation in CGRP^PBN^ cells, Calcr^NTS^ cells did not. Thus, Calcr^NTS^ neurons suppress food intake via the PBN but (unlike Cck^NTS^ cells) poorly activate aversive CGRP^PBN^ cells, consistent with their non-aversive suppression of food intake. These data thus reveal the existence of a population of non-CGRP PBN neurons that mediates the non-aversive suppression of food intake by Calcr^NTS^ cells.

### Calcr^NTS^ Cells Mediate the Suppression of Food Intake by a Variety of Stimuli and Contribute to Long-Term Energy Balance

To understand the potential roles for Calcr^NTS^ neurons in the control of food intake, we examined the afferent inputs to these cells by means of single-synapse retrograde tracing with defective rabies virus (Flak et al., 2017, Wickersham et al., 2007) (Figure S6). This analysis revealed strong inputs from the nodose ganglion and a variety of forebrain sites, including the PVH, lateral hypothalamic area (LHA), and central amygdala.

**Figure 6:**
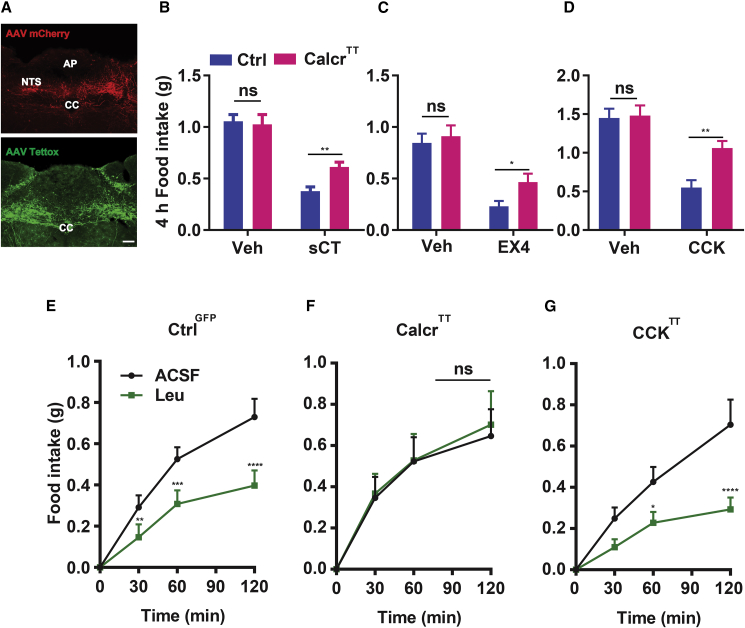
Calcr^NTS^ Neurons Contribute to the Suppression of Food Intake by Gut Peptide Receptors and Amino Acids (A) Representative images show viral transduction in control (Ctrl) (mCherry, red, top panel) and Calcr^NTS^-TT (Calcr^TT^) (GFP, green, bottom panel) mice. Scale bar, 150 μm; cc, central canal. (B–D) Food intake was measured over the first 4 h of treatment with (B) sCT (150 μg/kg, n = 18 in control group, n = 16 in Calcr^TT^ group), (C) EX4 (150 μg/kg, n = 14 in control group, n = 10-12 in Calcr^TT^ group), and (D) CCK (100 μg/kg; n = 5 in both groups). Mean ± SEM, unpaired t test was performed between Ctrl and Calcr^TT^ groups. ^∗^p < 0.05, ^∗∗^p < 0.01. (E–G) Suppression of food intake at the onset of the dark cycle in response to the intra-NTS injection of vehicle (ACSF) or Leu for control (E, GFP, n = 6), Calcr^TT^ (F, n = 7), and CCK^TT^ (G, n = 8) mice. Shown is mean ± SEM. Two-way ANOVA, Sidak’s multiple comparisons test was performed, ^∗^p < 0.05, ^∗∗^p < 0.01, ^∗∗∗^p < 0.001, ^∗∗∗∗^p < 0.0001. ns, not significant.

Because the innervation of Calcr^NTS^ cells by vagal afferents in the nodose ganglion suggested that Calcr^NTS^ cells receive input from the gut, we examined their activation by a variety of anorectic agonists for gut peptide receptors, including sCT, CCK, and exendin-4 (Ex4, a GLP1R agonist). Each of these peptides, as well as refeeding following a fast, increased FOS-IR in Calcr^NTS^ cells, as well as in other NTS cells (Figures S7A and S7B). To examine roles for Calcr^NTS^ cells in the anorectic response to these peptides, we bilaterally injected AVV^Flex−TetTox^ (which mediates the cre-dependent expression of tetanus toxin [TetTox] to prevent synaptic neurotransmitter release [Bergey et al., 1987, Kim et al., 2009]) into the NTS of *Calcr*^*cre*^ mice (Figure 6A). These Calcr^NTS^-TT mice exhibited an attenuated suppression of food intake in response to sCT, as expected (Figure 6B). Furthermore, Calcr^NTS^-TT mice also exhibit blunted suppression of food intake by CCK and Ex4 (Figures 6C and 6D). Thus, Calcr^NTS^ neurons contribute to the anorectic response to a variety of food-intake-suppressing gut peptide receptor agonists.

We also examined the potential role for Calcr^NTS^ cells in the strong appetite-suppressing effects of the amino acid Leu. Intra-NTS Leu (NTS-Leu) suppresses food intake (Blouet and Schwartz, 2012) without producing a CTA (Figure S7E). Indeed, we found that NTS-Leu promotes the activation of Calcr^NTS^ cells (Figures S7C and S7D). Furthermore, NTS-Leu failed to suppress food intake in Calcr^NTS^-TT mice, although NTS-Leu suppressed food intake normally in Cck^NTS^-TT mice with silenced Cck^NTS^ neurons (Figures 6E–6G).

**Figure 7:**
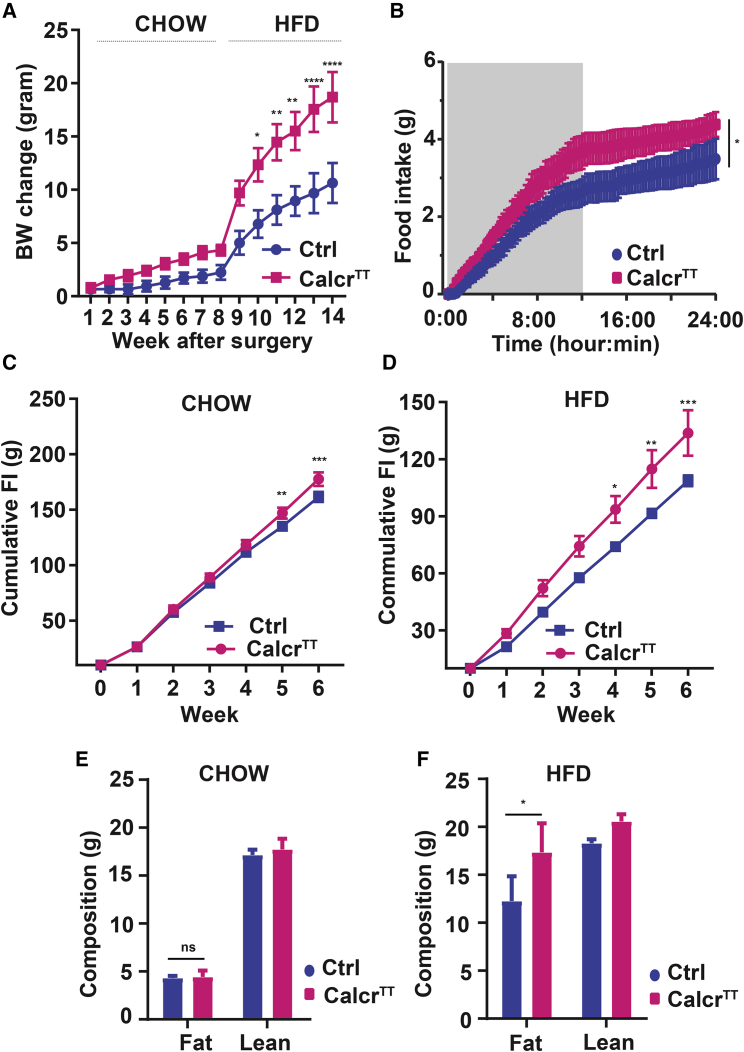
Silencing Calcr^NTS^ Neurons Increases Food Intake and Body Weight (A–F) Pairs of *Calcr*^*cre*^ mice were matched for initial body weight and injected with AAV^Flex−TetTox−GFP^ (Calcr^TT^) or AAV^Flex−mCherry^ (Ctrl). Mice were subjected to chow diet for 7 weeks, followed by another 6 weeks on HFD. (A) Body weight (change from baseline) for the duration of the experiment is shown (n = 10 in each group). (B) Food intake was continuously monitored over 24 h in a TSE system during the seventh week after surgery (n = 5 per group). Cumulative food intake on chow (C, n = 10 per group) and HFD (D, n = 10 per group) are shown. Body composition was determined after 7 weeks on chow diet (E, n = 7 in control group, n = 5 in Calcr^TT^ group) and 6 weeks on HFD (F, n = 5 per group). Mean ± SEM is shown. Two-way ANOVA, Sidak’s multiple comparisons test was performed in (A), (C), and (D). Two-way repeated-measures ANOVA was performed for (B). Unpaired t test was performed for (E) and (F). ^∗^p < 0.05, ^∗∗^p < 0.01, ^∗∗∗^p < 0.001, ^∗∗∗∗^p < 0.0001. ns, not significant.

To directly assess a potential role for Calcr^NTS^ neurons in energy homeostasis, we examined food intake and body weight in Calcr^NTS^-TT mice for 7 weeks on a chow diet, followed by an additional 6 weeks on HFD (Figure 7). We found that Calcr^NTS^-TT mice exhibited a tendency toward increased body weight on a chow diet. Indeed, the continuous analysis of food intake over 24 h in these mice revealed increased nocturnal food intake, and we also observed increased cumulative food intake over 7 weeks of home cage chow feeding (Figures 7B and 7C).

Furthermore, exposure to HFD resulted in a 25%–30% increase in food intake by Calcr^NTS^-TT mice compared to controls (Figure 7A) over the 6 weeks of HFD exposure, promoting an additional 8 g of weight gain compared to controls. Body composition analysis revealed that adipose mass represented the majority of the excess weight gain by Calcr^NTS^-TT mice (Figures 7E and 7F). Thus, silencing Calcr^NTS^ cells increases long-term food intake, body weight, and adiposity (especially in mice exposed to a palatable diet), revealing that these NTS neurons play an important role in overall energy balance, not just in the short-term suppression of food intake in response to gut-derived signals.

## Discussion

Our findings demonstrate that Calcr^NTS^ neurons mediate the long-term suppression of food intake by gut peptide mimetics and are required for the normal physiologic regulation of feeding and energy balance. Moreover, the activation of Calcr^NTS^ neurons is neither necessary nor sufficient for the acquisition of a CTA. In the context of the CTA-associated food intake suppression that results from the activation of Cck^NTS^ cells (Roman et al., 2017), our findings with Calcr^NTS^ neurons also demonstrate that the NTS systems that control the aversive and non-aversive suppression of feeding can be distinguished. Indeed, we show that while Calcr^NTS^ and Cck^NTS^ cells both inhibit MB-ARC neurons, Calcr^NTS^ cells do not activate aversive CGRP^PBN^ cells (as do Cck^NTS^ cells), but rather suppress food intake via distinct PBN targets. Our findings also reveal that Calcr^NTS^ cells not only participate in the short-term response to nutritional and gut peptide signals, but also play important roles in the long-term control of food intake and body weight, demonstrating a role for brainstem circuits in the control of overall energy balance, especially during exposure to HFD.

Because the activation of aversive symptoms can limit the utility of treatments designed to decrease food intake and body weight (Gianini et al., 2013), agents that activate neurons that suppress food intake without simultaneously stimulating aversive systems would represent ideal therapeutic agents. Hence Calcr^NTS^ cells, which contribute to the long-term suppression of food intake without provoking a CTA, represent a potentially useful target for the control of food intake. While the activation of *Gcg*-expressing NTS neurons in mice also suppresses food intake without activating a CTA, the decrease in food intake mediated by these cells is relatively small and transient (Gaykema et al., 2017), and their downstream targets have not been identified.

In contrast to the important role for *Calcr* in Calcr^NTS^ cells in CALCR agonist-mediated anorexia, *Calcr* in Sim1 or LepRb neurons is not required for this response to CALCR agonists. Furthermore, the undiminished PVH and ARC FOS responses to sCT in Calcr^Sim1^KO and Calcr^LepRb^KO mice, respectively, suggest that much sCT-induced hypothalamic FOS may be mediated indirectly, via *Calcr* neurons that project into the hypothalamus (potentially including Calcr^NTS^ cells). Furthermore, Calcr^LepRb^ neurons mainly represent orexigenic ARC NAG neurons (Pan et al., 2018), suggesting that the sCT-dependent direct activation of these cells might increase (rather than decrease) feeding. Indeed, we found that sCT slightly increases long-term food intake in Calcr^NTS^KO mice, consistent with the notion that sCT activates a set of orexigenic neurons (such as Calcr^LepRb^/NAG cells) but that this effect is normally masked by the anorexigenic action of Calcr^NTS^ cells. Consistently, we find that Calcr^NTS^ neuron activation overcomes feeding driven by ghrelin.

While Calcr^Sim1^ and Calcr^LepRb^ neurons are not required for the suppression of food intake by sCT, non-NTS *Calcr* neurons must participate in the acute anorectic response to sCT (during the first 1–2 h of treatment), even though Calcr^NTS^ cells mediate the long-term suppression of food intake. Indeed, AP lesions can attenuate the sCT-mediated suppression of food intake over 1–2 h in rats (Braegger et al., 2014), suggesting that sCT action via AP *Calcr* neurons might mediate the short-term attenuation of food intake in response to CALCR agonists. Similarly, because Calcr^NTS^ cells do not contribute to the aversive effects of sCT, mediate aversion, or contribute to the activation of CGRP^PBN^ cells, non-NTS Calcr cells must mediate the CGRP^PBN^-activating and aversive responses to sCT. Since the AP contributes to aversive signaling, including nausea (Wang et al., 2013), it is possible that Calcr^AP^ cells mediate the CTA-producing effects of sCT. Calcr^AP^ cells could also mediate the Calcr^NTS^-independent short-term suppression of food intake by sCT. Unfortunately, we and others have been unable to specifically target the AP by stereotaxic injection in mice, preventing us from directly testing this possibility.

To understand the potential circuit differences that underlie the aversive versus non-aversive suppression of food intake by Cck^NTS^ and Calcr^NTS^ cells, respectively, we first used MB-ARC FOS-IR as a surrogate to examine the control of NAG cells by Calcr^NTS^ neurons, since gut signals (that are presumably mediated by the hindbrain) inhibit these cells (Beutler et al., 2017) and because we found Calcr^NTS^ neuron activation blunts ghrelin-induced hyperphagia (which is mediated by NAG cells). Indeed, we found that the activation of Calcr^NTS^ neurons inhibited MB-ARC FOS-IR. While the ultimate circuit underlying this effect will require further studies, it is possible that Calcr^NTS^ cells project to and activate the GABAergic DMH cells that directly inhibit NAG cells (Garfield et al., 2016).

While the inhibition of MB-ARC neurons may contribute to the suppression of food intake by NTS neurons, Cck^NTS^ and Calcr^NTS^ cells similarly suppress MB-ARC neuron activity. Thus, the difference between the effects of Cck^NTS^ and Calcr^NTS^ neurons on aversion must lie in a distinct circuit. The DMH and/or PVH, both of which are innervated and activated by Cck^NTS^ and Calcr^NTS^ cells, could also contribute to the suppression of food intake by these NTS cells types. Indeed, activation of the Cck^NTS^→PVH circuit suppresses food intake (D’Agostino et al., 2016). Given that this projection from Cck^NTS^ cells is non-aversive (D’Agostino et al., 2016), however, these differences in PVH projections between Cck^NTS^ and Calcr^NTS^ cells are unlikely to underlie the difference in aversive signaling by the NTS cell types.

Because of the important roles played by CGRP^PBN^ cells in the aversive suppression of food intake (Campos et al., 2016), we examined the ability of Calcr^NTS^ neurons to suppress feeding via the PBN. While PBN projections from Calcr^NTS^ cells suppress feeding, these neurons innervate a distinct PBN region compared to Cck^NTS^ cells and poorly activate CGRP^PBN^ neurons compared to Cck^NTS^ cells. Hence, non-aversive, non-CGRP PBN neurons contribute to the anorectic effects of Calcr^NTS^ cells. Consistently, the PBN region activated by Calcr^NTS^ cells appears similar to that which relays a positive-valence vagal response to nutrient ingestion (Han et al., 2018).

Calcr^NTS^ neurons contribute to the suppression of food intake by a variety of gut peptide-mimetic stimuli (e.g., CCK and Ex4), as well as CALCR agonists. Calcr^NTS^ cells, but not Cck^NTS^ cells, also mediate the suppression of food intake by amino acids in the hindbrain (which do not produce a CTA). Although *Calcr* in Calcr^NTS^ neurons is not required for normal energy balance, our data reveal the requirement for signaling by Calcr^NTS^ neurons in the maintenance of long-term food intake control and energy homeostasis, especially in animals exposed to HFD. The importance of non-aversive Calcr^NTS^ cells to limit the consumption of HFD is not only consistent with the failure of silencing CGRP^PBN^ cells to alter long-term energy balance (Campos et al., 2016), but also suggests an important role for these cells in controlling the incentive value of palatable food. Thus, Calcr^NTS^ cells and their downstream circuits represent potentially useful therapeutic targets for the treatment of obesity.

Overall, Calcr^NTS^ neurons define a hindbrain system that suppresses food intake without activating aversive systems and participates in physiological energy balance (as well as in the anorectic response to pharmacologic activation). These results also demonstrate the separability of NTS circuits that mediate aversive anorexia from those that mediate the non-aversive suppression of food intake.

### Limitations of Study

Because hindbrain circuits differ in some respects between mice and rats (and presumably other mammals, including primates), it is possible that markers for NTS cell types that mediate aversive and non-aversive food intake suppression may differ by species. Furthermore, it will be important to identify the conditions under which, and mechanisms by which, the non-aversive NTS cells mediate their potential reinforcing effects.

## STAR★Methods

### Key Resources Table

**Table:** 

REAGENT or RESOURCE	SOURCE	IDENTIFIER
**Antibodies**		

FOS (DAB staining)	Santa Cruz	SC-52; RRID:AB_2106783
pSTAT3	Cell Signaling	9145; RRID:AB_2491009
FOS (Immunofluorescence)	Cell Signaling	2250; RRID:AB_2247211
CGRP	AbCam	Ab81887; RRID:AB_1658411
GFP	Aves Laboratories	GFP1020; RRID:AB_10000240
dsRed	Takara	632496; RRID:AB_10013483
TH	Novus Biologicals	NB300-109; RRID:AB_350437

**Bacterial and Virus Strains**		

Ad-iN-Syn-mCherry	Opland et al., 2013	N/A
AAVFlex-hM3Dq	Krashes et al., 2011	N/A
AAVFlexTetTox-GFP	Kim et al., 2009	N/A
AAVFlex-ChR2	Fenno et al., 2014	N/A
AAVFlex-GFP	Fenno et al., 2014	N/A
AAVFlexTVA+G	Flak et al., 2017	N/A
Defective pseudotyped rabies virus- tdTomato	Wickersham et al., 2007; University of Michigan Viral Vector Core	N/A
AAVFlex-hM4Di	Smith et al., 2016; AddGene	AddGene Cat#44362-AAV8

**Chemicals, Peptides, and Recombinant Proteins**		

CNO	Tocris	4936
sCT	Bachem	4033011
Davalintide	AstraZenica, Inc	N/A
PYY3-36	Bachem	4018889
LPS	Sigma	L2630
Exendin 4	Tocris	6355
CCK octapeptide (sulfated) ammonium	Bachem	H-2080
Leptin	AstraZenica, Inc	N/A
LiCl	Sigma	203637

**Experimental Models: Organisms/Strains**		

Calcr-cre	Pan et al., 2018	N/A
Calcr-flox	Yamaguchi et al., 2015	N/A
Cck-cre	Jackson laboratories	012706
Th-cre	Jackson laboratories	008601
NPY-Gfp	Pinto et al., 2004	N/A
AI14 (cre-inducible tdTomato)	Jackson laboratories	007914
Lepr-cre	Patterson et al., 2011; Jackson laboratories	032457
Sim1cre	Balthasar et al., 2005; Jackson laboratories	006451
Calca-cre-GFP	Carter et al., 2013	N/A

**Oligonucleotides**		

Calcr primers for qPCR: GCTGCTGGATGCTCAGTACA; AGTGTCGTCCCAGCACATC	IDT	N/A
Hprt primers for qPCR: GATTAGCGATGATGAACCAGGTT; CCTCCCATCTCCTTCATGACA	IDT	N/A

**Software and Algorithms**		

Prism v 7	Graphpad	N/A https://www.graphpad.com/scientific-software/prism/
Harmony	Perkin Elmer	www.perkinelmer.com/product/harmony
Photoshop	Adobe	www.adobe.com/products/photoshop.html
ImageJ	NIH	https://imagej.nih.gov/ij/download.html

### Lead Contact and Materials Availability

Further information and requests for resources and reagents should be directed to and will be fulfilled by the Lead Contact, MGM (mgmyers@umich.edu). This study did not generate new unique reagents.

### Experimental Model and Subject Details

#### Animals

Mice were bred in our colony in the Unit for Laboratory Animal Medicine at the University of Michigan (except as noted below for mice tested at the University of Cambridge); these mice and the procedures performed were approved by the University of Michigan Committee on the Use and Care of Animals and in accordance with Association for the Assessment and Approval of Laboratory Animal Care and National Institutes of Health guidelines. Mice at both sites were provided with food (Purina Lab Diet 5001, unless otherwise specificed) and water *ad libitum* (except as noted below) in temperature-controlled (25^o^C) rooms on a 12 h light-dark cycle with daily health status checks.

We purchased male and female C57BL/6 mice for experiments and breeding from Jackson Laboratories. *Calcr*^*cre*^,*Calcr*^*flox*^, and *Cck*^*cre*^ mice have been described (D’Agostino et al., 2016, Pan et al., 2018, Roman et al., 2016, Yamaguchi et al., 2015) and were propagated by intercrossing homozygous mice of the same genotype. *Th*^cre^ mice (Goodwin et al., 2019) were purchased from Jackson Laboratory (Stock No: 008601). NPY–green fluorescent protein (GFP) transgenic mice are as described (Pinto et al., 2004). Cre-induciable tdTomato reporter mice (AI14) were purchased from Jackson Laboratory (Stock No: 007914), and were crossed with *Calcr*^*cre*^ mice to generate Calcr^cre^; tdTomato reporter mice.

*Calca*^*cre-GFP*^ mice, the generous gift of Richard Palmiter (University of Washington) have been described (Carter et al., 2013) and were crossed with *Calcr*^*cre*^ or *Cck*^*cre*^ to generate *Calca*^*cre-GFP/+*^*; Calcr*^*cre/+*^ or *Calca*^*cre-GFP/+*^*; Cck*^*cre/+*^ mice to permit tracing and FOS studies with the visualization of *Calca*-containing CGRP^PBN^ neurons.

*Calcr*^*flox*^ mice were crossed twice onto the *Lepr*^*cre*^ (Patterson et al., 2011) background to generate *Lepr*^*cre/cre*^*;Calcr*^*flox/+*^ mice, which were intercrossed to generate *Lepr*^*cre/cre*^*;Calcr*^*flox/flox*^ (Calcr^LepRb^KO) and *Lepr*^*cre/cre*^ control mice. *Calcr*^*flox*^ mice were also crossed twice onto *Sim1*^*cre*^ (Balthasar et al., 2005) to generate *Sim1*^*cre/+*^*;Calcr*^*flox/flox*^ mice, which were bred to *Calcr*^*flox/flox*^ mice to generate *Sim1*^*cre/+*^*;Calcr*^*flox/flox*^ (Calcr^Sim1^KO) and *Calcr*^*flox/flox*^ controls for study. For all studies, animals were processed in the order of their ear tag number, which was randomly assigned at the time of tailing (before genotyping). All mouse models (with the exception of purchased C57BL/6 mice) were on the segregating C57BL/6;SJL;129 genetic background; sibling controls were used throughout. Male and female mice were used for all experiments where a single sex was not indicated; no sex differences in phenotype were detected.

For experiments performed at the University of Cambridge, all mice were single-housed in individually ventilated cages with standard bedding and enrichment in a temperature and humidity-controlled room on a 12-h light/dark cycle with *ad libitum* access to water and standard laboratory chow diet unless otherwise stated. 8-weeks old C57/Bl6J males were obtained from Charles River UK. All studies were approved by the local Ethics Committee and conducted according to the UK Home Office Animals (Scientific Procedures) Act 1986.

### Method Details

#### Viral Reagents and Stereotaxic Injections

Ad-iN-Syn-mCherry was generated and produced as described previously (Opland et al., 2013). AAV^Flex-hM3Dq^ (Krashes et al., 2011), AAV^Flex-TetTox-GFP^(Kim et al., 2009), AAV^Flex-ChR2^, AAV^GFP^ and AAV^Cre-mCherry^ (Fenno et al., 2014) were as previously described, and were prepared by the University of North Carolina Vector Core (Chapel Hill, NC). AAV^Flex-TVA+G^ and defective psuedotyped rabies-mCherry (Flak et al., 2017, Wickersham et al., 2007) were generated by the University of Michigan viral vector core. AAV^Flex-hM4Di^ (Smith et al., 2016) was ordered through Addgene (Catalog #: 44362-AAV8).

For injection, following the induction of isoflurane anesthesia and placement in a stereotaxic frame, the skulls of adult mice were exposed. For NTS injection, the obex was set as reference point for injection. After the reference was determined, a guide cannula with a pipette injector was lowered into the approximate NTS coordinates, which was A/P, −0.2; M/L, ± 0.2; D/V, −0.2 from the obex and 100 nL of virus was injected with using a picospritzer at a rate of 5-30 nL/min with pulses. Five minutes following injection, to allow for adequate dispersal and absorption of the virus, the injector was removed from the animal; the incision site was closed and glued. The mice received prophylactic analgesics before and after surgery (carprofen, 5 mg/kg, SQ). Mice were 8-12 weeks of age at the time of injection and were studied beginning 3-4 weeks post-surgery.

The mice injected with Ad-iN-Syn-mCherry were allowed one week to recover before being euthanized; the mice injected with AAV^Flex-hM3Dq^, AAV^Flex-hM4Di^, AAV^Flex-ChR2^, AAV^Cre-mCherry^, AAV^Flex-TetTox-GFP^, or control viruses were allowed at least 1 week to recover from surgery before experimentation.

#### Optogenetics

As described above, AAV^Flex-ChR2^ virus was delivered in NTS for *Calcr*^*cre*^ and *Cck*^*cre*^ mice, two fiber-optic cannulae (Doric Lenses and Thorlabs) were implanted above the lateral PBN (A/P: 5.2 mm, M/L: ± 1.6 mm, D/V: 2.9 mm) and affixed to the skull using Metabond (Fisher). After 3 weeks recovery from surgery, the mice were then subjected to optical stimulation using 473 nm wavelength by 1 s of 20Hz photo stimulation and 3 s resting with multiple repetitions for 1 to 2 h period of time, during which time food intake was measured.

#### Chronic Cannula Implantation for NTS Injections

Surgical procedures were performed under isoflurane anesthesia on animals that were 12-16 weeks of age. Animals were stereotaxically implanted with bilateral steel guide cannulae (Plastics One) positioned 2.0 mm above the caudomedial nucleus of the solitary tract (cannula holding bar in a 10° rostro-caudal angle, coordinates relative to occipital suture: A/P +0.5 mm, D/V-3 mm. +/− 0.4 lateral to midline). Beveled stainless steel injectors (33 gauge mounted onto a 26-gauge sleeve) extending 2.0 mm from the tip of the guide cannulas were used for injections. Animals were allowed a 1-week recovery during which they were handled daily and acclimatised to the brain injection procedure. Correct bilateral NTS cannula placement was confirmed histologically postmortem. Mice were randomly assigned to treatment groups and received a bilateral injection of 50nl/side vehicle (aCSF, Harvard Apparatus) or 2.1mM leucine via the pre-implanted cannula targeting the NTS in a cross-over manner by an experimenter blinded to genotype and treatment. Mice were fasted for 6 h before the injections and injections occurred 1 h before dark onset as previously described (Cavanaugh et al., 2015).

#### Phenotypic Studies

Animals were singly housed from the time of weaning (Calcr^LepRb^KO and Calcr^Sim1^KO) or beginning 7 days after surgery (Calcr^NTS^KO). Food intake and body weight were monitored weekly. Calcr^NTS^TetTox mice and their controls were monitored from the time of surgery, and were also studied for meal patterns in metabolic chambers (TSE Systems Inc) during the final week of chow feeding.

For stimulation studies, KO mice, DREADD-expressing mice and their controls that were either at least two-months old or two months post-surgery were treated with saline or drugs (CNO, 4936, Tocris; sCT, 4033011, Bachem; Davalintide and Leptin, AstraZenica/MedImmune from original lots produced at Amylin Pharmaceuticals; PYY3-36, 4018889, Bachem; LPS, L2630, Sigma; Exendin 4, 6355, Tocris; Cholecystokinin Octapeptide (sulfated) ammonium, H-2080, Bachem) at the onset of dark cycle, food intake was monitored over four h. For chronic food intake and body weight changes, mice were given saline for two to three days prior to injecting saline or drugs twice per day (approximately 5:30 PM and 8:00 AM) for 3 or 4 days, followed by saline injections for another two or three days to assess recovery from the treatment.

#### Perfusion and Immunohistochemistry

Mice (8 weeks of age or older) were anesthetized with a lethal dose of pentobarbital and transcardially perfused with phosphate-buffered saline (PBS) followed by 10% buffered formalin. Brains were removed, placed in 10% buffered formalin overnight, and dehydrated in 30% sucrose for 1 week. With use of a freezing microtome (Leica, Buffalo Grove, IL), brains were cut into 30-μm sections. Sections were treated sequentially with 1% hydrogen peroxide/0.5% sodium hydroxide, 0.3% glycine, 0.03% sodium dodecyl sulfate, and blocking solution (PBS with 0.1% triton, 3% normal donkey serum). The sections were incubated overnight at room temperature in rabbit anti-FOS (Santa Cruz, sc-52; 1: 1,000), and exposed the next day with either biotinylated (1:200 followed by avidin-biotin complex (ABC) amplification and 3,3-diaminobenzidine (DAB) reaction) or fluorescent secondary antibody (Molecular Probes, 1:200) to visualize proteins. Immunofluorescent staining was performed using primary antibodies (pSTAT3, #9145, Cell Signaling Technology, 1:250; FOS, #2250, Cell Signaling Technology, 1:1000; CGRP, ab81887, 1:1000, Abcam; GFP, GFP1020, Aves Laboratories, 1:1000; dsRed, 632496, Takara, 1:1000; Tyrosine Hydroxylase, NB300-109, Novus Biologicals, 1:1000), antibodies were reacted with species-specific Alexa Fluor-488, −568 or −647 conjugated secondary antibodies (Invitrogen, Thermo Fisher, 1:200). Images were collected on an Olympus (Center Valley, PA) BX53F microscope. images were pseudocolored using Photoshop software (Adobe) or ImageJ (NIH).

#### RNA Extraction, Reverse Transcription and RT-qPCR

Calcr^Sim1^KO and control mice (8 weeks of age or older) were euthanized with inhalation anesthetic isoflurane (McKesson, Piramal Critical Care #6679401725) using a drop jar. Microdissected PVH tissue for RNA preparation was stored in a −80°C freezer. RNA extraction was conducted using an RNeasy Mini kit (QIAGEN) and quantified using a NanoDrop 1000 (Thermo Scientific). RNA was treated with DNase (TURBO DNA-free Kit, Thermo Fisher Scientific, Cat #AM1907) and again quantified using a NanoDrop 1000. Approximately 500 ng of total RNA per sample underwent reverse transcription (iScript cDNA Synthesis Kit, Bio-Rad, Cat #1708891) using a thermal cycler (Mastercycler pro S, Eppendorf). Quantitative PCR (qPCR) was conducted using a StepOnePlus Real Time PCR System (Applied Biosystems) with SYBR Green PCR Master Mix (Applied Biosystems, Cat #4309155) and CalcR primers (Forward: 5′-GCTGCTGGATGCTCAGTACA-3′, Reverse: 5′-AGTGTCGTCCCAGCACATC-3′). The hypoxanthine guanine phosphoribosyl transferase (*Hprt*) gene was used as a reference gene (amplified using primers; Forward: 5′-GATTAGCGATGATGAACCAGGTT-3′, Reverse: 5′-CCTCCCATCTCCTTCATGACA-3′). A standard curve ladder was generated by pooling the cDNA from each sample and making 5 serial dilutions. From this, a standard curve line of best fit was produced, which allowed us to determine the relative RNA concentration of each sample. Samples were run in duplicate. These values were subjected to a Grubbs’ test to discard any of the outliers before conducting statistical analysis.

#### Conditioned Taste Aversion (CTA)

Two-flavoured drink CTA was performed as described (Halatchev and Cone, 2005). Briefly, mice (12 weeks of age or older) were individually housed in standard cages with low wire tops and free access to food and the lixits were removed. Mice were habituated to two water-containing bottles for 3-5 days until they learned to concentrate intake at 1000 h–1100 h. On conditioning days, mice were given 1 h access to either 0.05% cherry Kool-Aid paired with saline or grape Kool-Aid paired with the stimulus (e.g., LiCl, peptides, or CNO (0.8 mg/kg, 4936, Tocris Bioscience)). Mice were conditioned with these novel drinks for two rounds. Post conditioning day, mice were provided free access to the two flavoured drinks and two h intake were measured for each tastant.

Saccharin CTA: Mice that were 8 weeks of age or older were individually housed in standard cages with low wire tops and free access to food and the lixits were removed. Mice were habituated to two water-containing bottles for 3-5 days until they learned to concentrate their daily water consumption into these two water bottles. On the conditioning day, the mice received only two saccharin (0.15%, 240931, Sigma) bottles. Following the 30 min exposure to saccharin, mice were injected intraperitoneally with the desired stimulus (vehicle control (0.9% NaCl), lithium chloride (0.3 M, 203637, Sigma)) in a volume equivalent to 1% of each animal’s body weight (10 μL/g), or CNO, or sCT (150 μg/kg, 4033011, Bachem). Access to the two saccharin bottles continued for an additional 2 h, followed by the return of normal water bottles. Two days later, each mouse received access to two water bottles (one containing 0.15% saccharine, the other containing water), and the amount of fluid ingested from each water bottle was be measured.

High fat diet (HFD) CTA: following an overnight fast, mice that were 8 weeks of age or older were conditioned with HFD and paired with desired stimuli for 30 min following with an extra one h access for HFD (D012492, Research Diets). On the post-conditioning day, fasted mice received access to both HFD and chow and the consumption of each was measured.

#### CTA for Leu Injection

C57/Bl6J mice (n = 6 per group) implanted with a cannula targeting the NTS were singly housed with free access the two water bottles for 7 days. On days 1-3, mice of 12 weeks of age or older were water-deprived overnight and allowed access to water for 60 min in the morning and free access in the afternoon. On day 4, after overnight water deprivation, mice received an injection of aCSF or Leucine into the NTS (as above) followed by exposure to water containing 5% sucrose with either 0.05% grape or cherry flavour for 60 min. This was repeated 3 days later in a cross-over manner. On days 5-7 and 8-10, mice were exposed to the same regimen as on days 1-3. On day 8, after overnight water deprivation, mice were given access to a choice of two water bottles containing 5% sucrose with 0.05% grape or cherry flavour for 60 min and intake was recorded. ip LiCl was used as a positive control to induce conditioned taste aversion with the same paradigm.

#### *In Situ* Hybridization (ISH)

Three adult female *Cck*^*Cre/+*^*;TdTomato/+* mice of at least 8 weeks of age were anesthetized with isoflurane and then euthanized by decapitation. Brains were dissected, flash frozen in isopentane chilled on dry ice, and stored at −80°C. Sections were sliced at 16 μm thickness using a cryostat (Leica) and every fourth section was thaw-mounted onto SuperFrost Plus slides, allowed to dry for one h at −20°C, and then further stored at −80°C. Slides were then processed for RNAScope ISH per the manufacturer’s protocol (Advanced Cell Diagnostics). The multiplex fluorescent assay (320850) was used to visualize *Calcr* (477791) and Cre (312281-C3) probes using Amp 4 Alt-A. At each of 8 coronal planes for each mouse, 4 images comprising the entire NTS/AP complex were obtained with a QImaging Retiga 6000 monochrome camera attached to an Olympus BX53 fluorescent microscope under 20X objective. The four images were then stitched together using Photoshop (Adobe). CellProfiler (Lamprecht et al., 2007) was used to process all images identically to remove nonspecific background, outline specific cells using the DAPI nuclear signal and analyze presence or absence of signal for both probes. For each mouse, the total number of cells identified as positive for either or both probes were added from all 8 coronal planes (for NTS) or for the subset of the 6 planes where AP was present. Subsequently, the sums from the three mice were averaged for each region.

#### Multiplexed FISH with RNAscope for Leu Related Experiments

Brains were postfixed in 4% PFA solution overnight then cryoprotected in 30% sucrose solution in PBS for up to 24 h. Tissue was covered with optimal cutting temperature (OCT) media then sliced at 16 μm thickness using a Leica CM1950 cryostat directly onto Superfrost Plus slides (ThermoScientific) in an RNase free environment. Slides were then stored at −80°C. Sections were sliced in the coronal plane from Bregma −1.58 to −2.30 mm (Franklin, K. and Paxinos, G., 2008).

Fluorescence multiplex *in situ* RNA hybridization (FISH) was performed as previously described using RNAscope technology (Wang et al., 2012). After epitope retrieval and dehydration, sections on slides were processed for multiplexed FISH using the RNAScope LS Multiplex Assay (Advanced Cell Diagnostics). Samples were first permeabilized with heat in Bond Epitope Retrieval solution 2 (pH 9.0, Leica - AR9640) at 95°C for 2 min, incubated in protease reagent (Advanced Cell Diagnostics) at 42°C for 10 min, and finally treated with hydrogen peroxide for 10 min to inactivate endogenous peroxidases and the protease reagent. Samples were then incubated in z-probe mixtures (FOS 1:1, Calcr 1:50) for 2 h at 42°C and washed 3 times. DNA amplification trees were built through incubations in AMP1 (preamplifier), AMP2 (background reducer), then AMP3 (amplifier) reagents (Leica) for 15-30 min each at 42°C. Between incubations, slides were washed with LS Rinse buffer (Leica). After, samples were incubated in channel-specific horseradish peroxidase (HRP) reagents for 15 min at 42°C, TSA fluorophores for 30 min and HRP blocking reagent for 15 min at 42°C. The following TSA labels were used to visualize z-probes: Cy3 (1:500), FITC (1:500), and Cy5 (1:500) fluorophores (Perkin Elmer).

Brain sections were imaged using a spinning disk Operetta CLS (Perkin Elmer) in confocal mode using a sCMOS camera and a 40x automated-water dispensing objective. Sections were imaged with z stacks at intervals of 1 μm. ROIs included the NTS, AP and DMX. Gain and laser power settings remained the same between experimental and control conditions within each experiment. Harmony software (Perkin Elmer) was used to automatically quantify number of labeled RNA molecules (spots) per cell, and number of labeled cells.

### Quantification and Statistical Analysis

#### Statistics

Data are reported as mean ± standard error of the mean. Statistical analyses of physiologic data were performed with Prism software (version 7), including testing to ensure the data fit a normal distribution. Two way ANOVA, paired or unpaired t tests were used as indicated in the text and figure legends. p < 0.05 was considered statistically significant.

### Data and Code Availability

This study did not generate any unique datasets or code.

## Supplemental Information

Document S1. Figures S1–S7

Document S2. Article plus Supplemental Information
