# Supplementary material for: Calcitonin Receptor Neurons in the Mouse Nucleus Tractus Solitarius Control Energy Balance via the Non-aversive Suppression of Feeding
Source: Cell Metab. 2020 Feb 4;31(2):301–312.e5. doi: 10.1016/j.cmet.2019.12.012 (PMC7104375; doi:10.1016/j.cmet.2019.12.012)
Supplement: Document S1. Figures S1–S7 [file mmc1.pdf]

Cell Metabolism, Volume *31*

Supplemental Information

Calcitonin Receptor Neurons in the Mouse Nucleus Tractus Solitarius Control Energy Balance via the Non-aversive Suppression of Feeding

Wenwen Cheng, Ian Gonzalez, Warren Pan, Anthony H. Tsang, Jessica Adams, Ermelinda Ndoka, Desiree Gordian, Basma Khoury, Karen Roelofs, Simon S. Evers, Andrew MacKinnon, Shuangcheng Wu, Henriette Frikke-Schmidt, Jonathan N. Flak, James L. Trevaskis, Christopher J. Rhodes, So-ichiro Fukada, Randy J. Seeley, Darleen A. Sandoval, David P. Olson, Clemence Blouet, and Martin G. Myers Jr.

Supplemental Figure 1

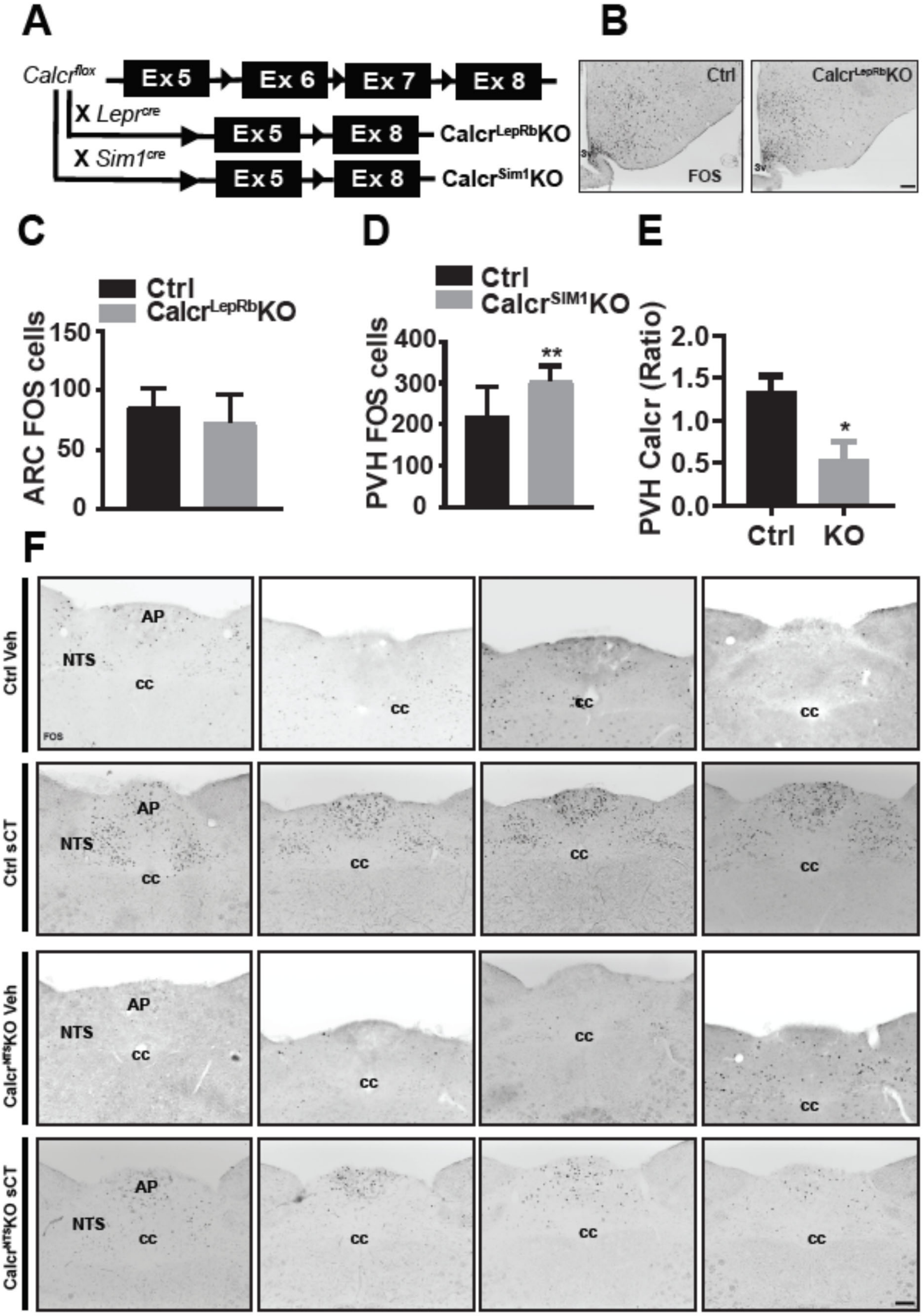

Supplemental Figure 1 (Related to Figure 1): FOS response to sCT in *Calcr* knock-out lines. (A) Schematic diagram showing the cross of *Calcr*^{flox} with *Lepr*^{cre} and *Sim1*^{cre} mice to generate Calcr^{LepRb}KO and Calcr^{Sim1}KO mice. **(B)** Representative images showing FOS–IR in the hypothalamus of control (Ctrl) and Calcr^{LepRb}KO mice following treatment with sCT (150 µg/kg, IP). Scale bar equals 150 µm; 3V- third cerebral ventricle. **(C)** Quantification of ARC FOS-IR cells 2 hours after sCT (150 µg/kg) injection in Calcr^{LepRb}KO (n=5) and control (Ctrl, n=6) mice. (**D**) Quantification of PVH FOS-IR cells 2 hours after sCT injection (100 µg/kg, IP) in Ctrl (n=7) and Calcr^{SIM1}KO (n=9) mice. (**E**) Quantification of Calcr mRNA by qRT-PCR in PVH microdissections from Ctrl (n=6) and Calcr^{sim1}KO (n=7) mice. C, D, E: Mean +/- SEM is shown; *p<0.05, **p<0.01 by unpaired t-test. **(F)**. *Ad lib* control and Calcr^{NTS}KO mice were treated with vehicle (Veh) or sCT (150 µg/kg, IP) and were perfused for immunostaining for FOS two hours later. Sections from four representative mice are shown for each genotype/condition. cc-central canal. Scale bar equals 150 µm.

Supplemental Figure 2

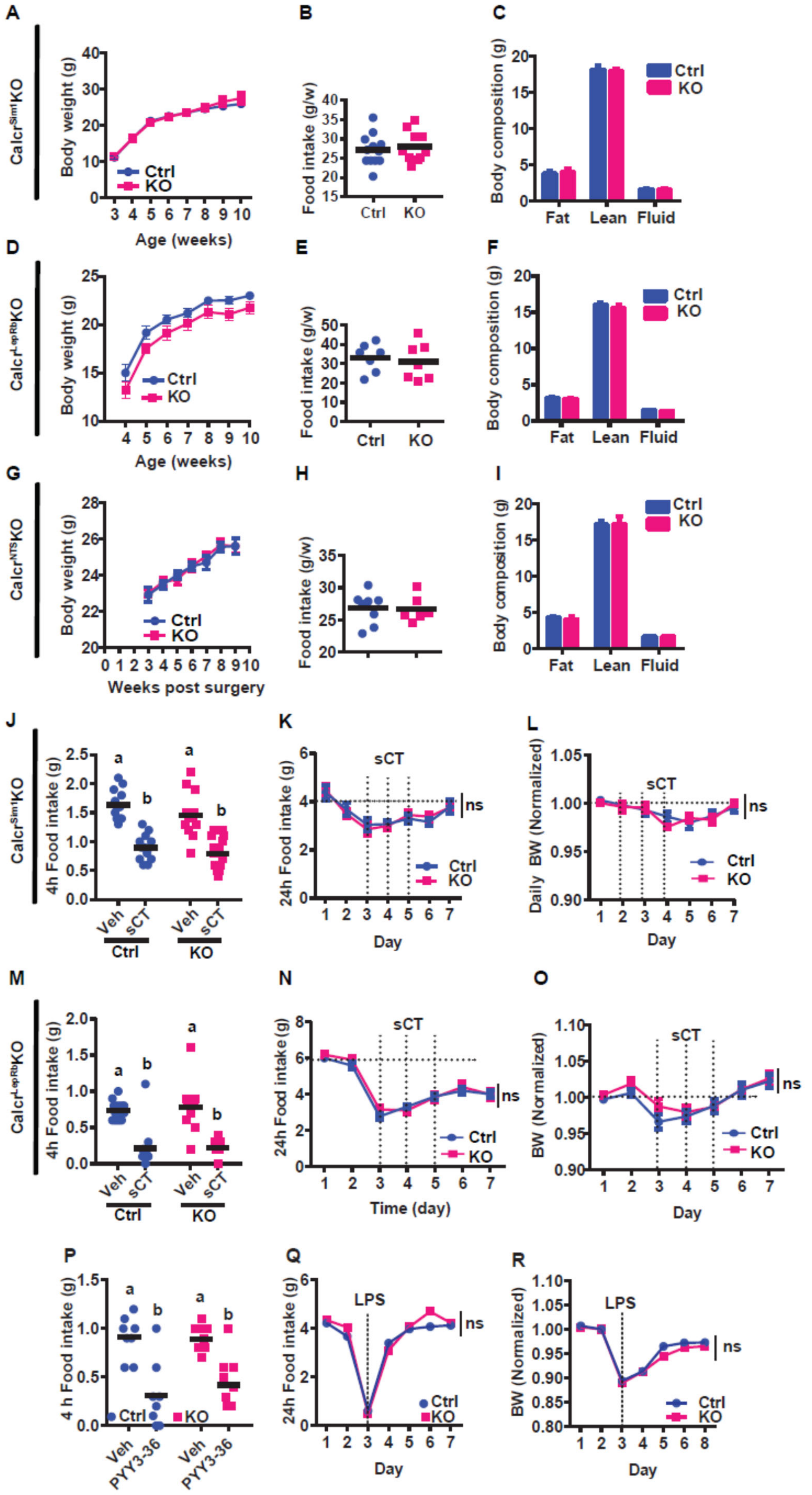

Supplemental Figure 2 (Related to Figure 1): Energy balance and response to anorectic agents in CalcrKO mouse lines. (A-C) Control and $Calcr^{Sim1}$KO mice were monitored until 10 weeks of age for body weight **(A),** food intake **(B),** and body composition (14-18 weeks of age) **(C)**; n=4-10 per group. (**D-F**) Control and $Calcr^{LepRb}$KO mice were monitored until 10 weeks of age for body weight **(D),** food intake **(E),** and body composition (10 weeks of age) **(F)**; n=6-13 per group. (**G-I**) Control and $Calcr^{NTS}$KO mice were monitored for 7 weeks following surgery for body weight **(G)** and food intake **(H).** Body composition was determined nine weeks post-surgery **(I)**; n=6-8 per group. One-way ANOVA, Tukey's multiple comparisons was performed in A, D and G; Two-way ANOVA, Sidak's multiple comparisons test was performed in B, C, E, F, H, and I. All comparisons, p=not significant (ns). Responses to sCT (**J-O**): Food intake for the first 4h of the dark cycle (**J, M**) and daily food intake (**K, N**) and body weight (change from baseline) (**L, O**) during chronic treatment with IP vehicle (Veh) or sCT twice per day in $Calcr^{Sim1}$KO (100 μg/kg sCT BID) (J-L) and $Calcr^{LepRb}$KO (150 μg/kg sCT BID) (M-O). Dotted vertical lines denote days of sCT treatment; horizontal dotted lines denote baseline values. Individual replicates plus mean is shown for (J, M). Mean +/- SEM is shown for all other panels; n=13-17 per group in (J-L), n=9-15 per group in (M-O). One-way ANOVA, Tukey's multiple comparisons was performed in J and M, different letters indicate differences $p<0.05$ for the indicated groups; Two-way ANOVA, Sidak's multiple comparisons test was performed in K, L, N and O. ns: not significant. Effects of PYY3-36 and LPS on food intake and body weight in $Calcr^{NTS}$KO mice. **(P-R) (P)** Control (Ctrl, Blue) and $Calcr^{NTS}$KO (KO, Red) mice were treated with PYY3-36 (560 μg/kg, IP) and food intake was measured for 4 hours at the onset of the dark cycle; n=8 per group. **(Q, R)** Control (Ctrl, Blue) and $Calcr^{NTS}$KO (KO, Red) mice were treated with LPS (300 μg/kg) and food intake and body weight were measured for subsequent days; n=8-10 per group. Mean +/- SEM is shown. One-way ANOVA, Tukey's multiple comparisons in P, different letters indicate difference, $p<0.05$. Two-way ANOVA, Sidak's multiple comparisons test was performed in Q and R. **$p<0.01$, ***$p<0.001$, ****$p<0.0001$, ns: not significant.

Supplemental Figure 3

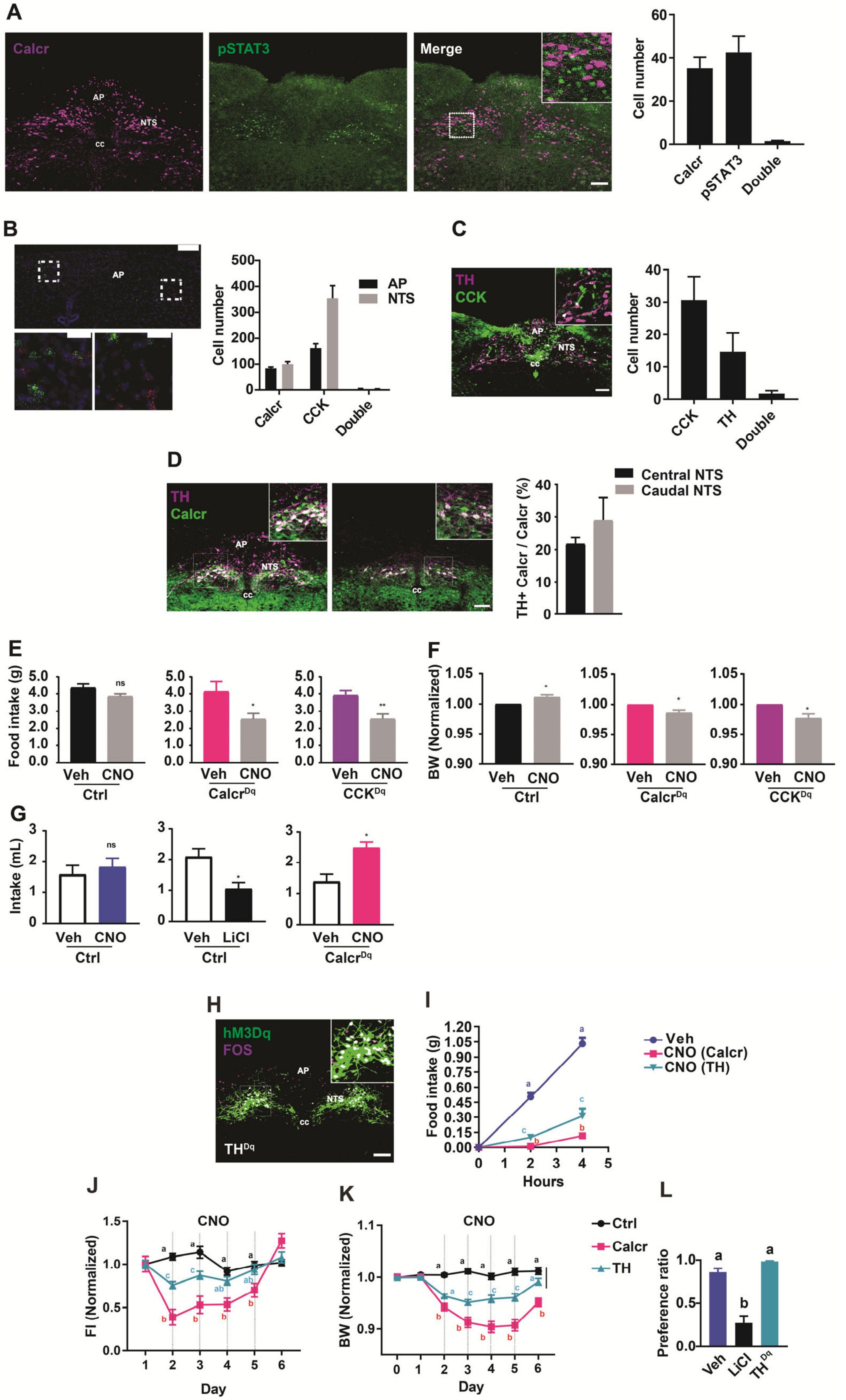

Supplemental Figure 3 (Related to Figure 2): Calcr^{NTS} neurons overlap with TH, but not CCK or LepRb neurons, and food intake following activation of Calcr^{NTS}, TH^{NTS}, or CCK^{NTS} neurons. (A) Representative images of the central NTS stained for pSTAT3 (green) and tdTomato (purple) in *Calcr^{cre};tdTomato* reporter mice treated with Leptin (5 mg/kg, IP, 2 hours). Calcr positive, pSTAT3 positive and double positive cell number were counted unilaterally in the central NTS. Mean +/- SEM is shown; n=3. cc- central canal; scale bar equals 150 μm. **(B)** Representative stitched image for RNAScope fluorescent ISH analysis of *Cre* (red) and *Calcr* (green) in the NTS of *Cck^{cre}* mice; blue is DAPI. Scale bar = 100 μm in large image, 25 μm in insets; cc- central canal. Cells positive for *Calcr*, *Cre*, or both were quantified in the AP and NTS. Mean +/- SEM is shown; n=3. (**C**) Representative image of the central and caudal NTS stained for TH (purple) and tdTomato (green) in *Calcr^{cre};tdTomato* reporter mice. Scale bar equals 150 μm; cc- central canal. Percent of *Calcr* (tdTomato) cells also containing TH-IR was quantified for each NTS region. Mean +/- SEM is shown; n=3. (**D**) Representative image of the central NTS stained for TH (purple) and tdTomato (green) in *CCKcre;tdTomato* reporter mice, scale bar eques 150 μm. Cells positive for *CCK*, *TH*, or both were quantified in the central NTS unilaterally. Mean +/- SEM is shown; n=3. **(E, F)** 24-hour response to a single dose of vehicle (Veh) or CNO (0.3 mg/kg, IP) in a crossover design at the onset of the dark cycle in control (Ctrl), Calcr^{Dq}, and CCK^{Dq} mice. Food intake was determined after 24 hours (E) and body weight was determined at baseline and after 24 hours (F). Mean +/- SEM is shown; n=6 in control group, n=6 in calcr group, n=7 in cck group. **(G)** Intake data for Figure 2G: Control (Ctrl; left and middle panel) or Calcr^{Dq} mice (right panel) were treated on alternating days with vehicle (Veh) paired with one randomly assigned flavor or stimulus (CNO (1 mg/kg) or LiCl (126 mg/kg)) paired with a different flavor for three conditioning sessions each. Consumption was measured for each flavor on the first post conditioning day. Mean +/- SEM is shown; n=11 in ctrl-CNO, n=12 in LiCl, n=8 in Calcr-Dq group. Paired t-test (E, F) or unpaired t-test (G), *p<0.05, **p<0.01, ns- not significant. **(H)** Representative images showing FOS (purple) and hM3Dq (green) 2 hours after CNO injection (1 mg/kg, IP) in TH^{NTS}-Dq (TH^{Dq}) mice. Scale bar equals 150 μm; cc- central canal. **(I)** Food (chow) intake during the first 4 hours of the dark cycle following treatment of Th^{Dq} mice with vehicle (Veh; n=23-25) and CNO (1 mg/kg, IP, n=8) compared with food intake in CNO-treated Calcr^{Dq} mice from Figure 2 (n=18-20). (**J, K**) Daily food intake (J) and body weight (K) were measured during 3 days of vehicle, 4 days of CNO (IP, 1 mg/kg, BID), and 2 additional days of vehicle injection for control (Ctrl; n=14) and TH^{Dq} mice (n=8), along with the Calcr^{Dq} mice plus Calcr^{Dq} mice from Figure 2 (n=13). **(L)** CTA was also determined by pairing CNO (1mg/kg, IP) injected TH^{Dq} mice with novel HFD (n=8 per group). Mean +/- SEM is shown. Two-way ANOVA, Sidak's multiple comparisons test was performed in I, J, and K; one-way ANOVA, Tukey's multiple comparisons was performed in L. Different letters indicate differences p<0.05.

Supplemental Figure 4

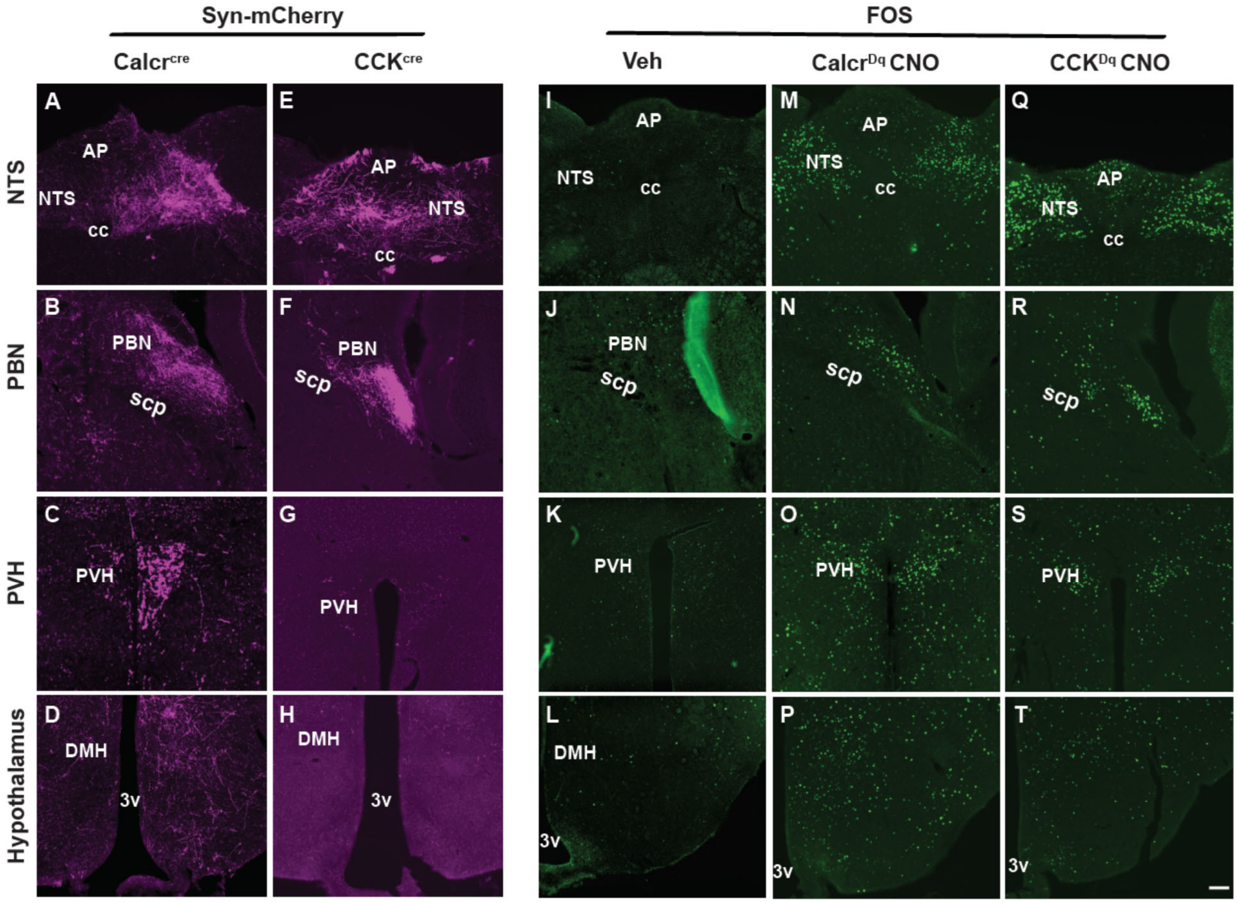

Supplemental Figure 4 (Related to Figure 5): Downstream targets of Calcr^{NTS} and CCK^{NTS} neurons. Following the injection of AAV$^{Flex\text{-}Syn\text{-}mCherry}$ into the NTS of Calcr^{cre} **(A-D)** or CCK^{cre} **(E-H)** mice, brains were sectioned and stained for mCherry (Purple). Representative images of major projection targets are shown. (**I-T**) Representative images showing FOS-IR (green) in major projection targets of Calcr^{NTS} neurons in Calcr^{NTS}-Dq **(M-P)** and CCK^{NTS}-Dq **(Q-T)** mice following injection with vehicle (Veh) **(I-L)** or CNO (0.8 mg/kg, IP, AM) (**M-T**). Scale bar equals 150 μm; cc= central canal, scp=superior cerebellar peduncle, 3V- third cerebral ventricle. PBN=parabrachial nucleus, PVH=paraventricular hypothalamus. DMH=Dorsal medial hypothalamus. Images are representative of 3 per group.

Supplemental Figure 5

a

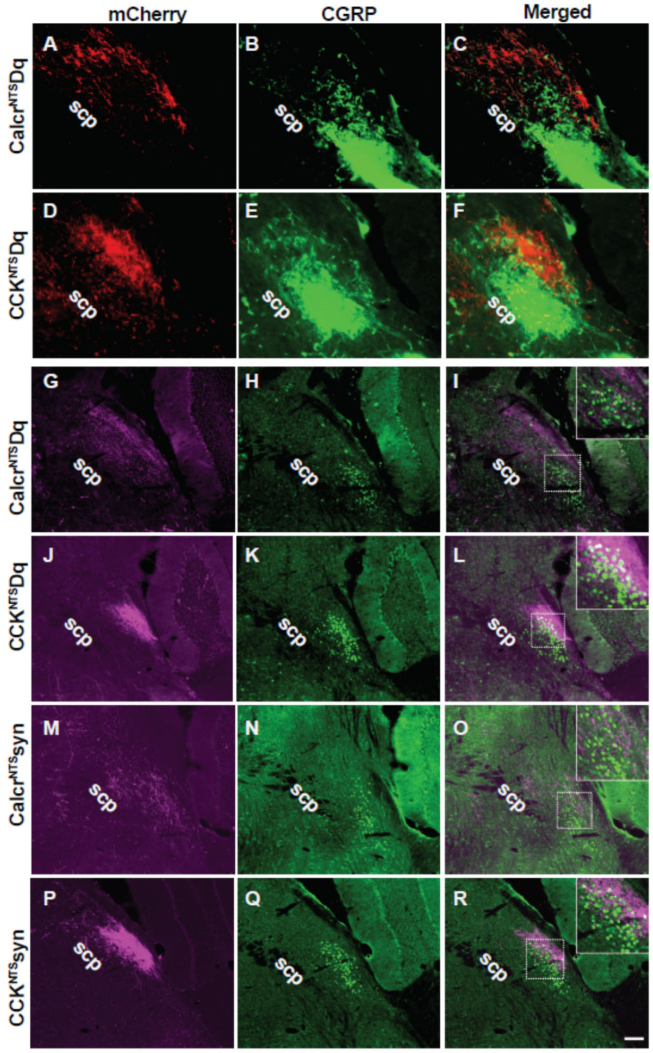

b

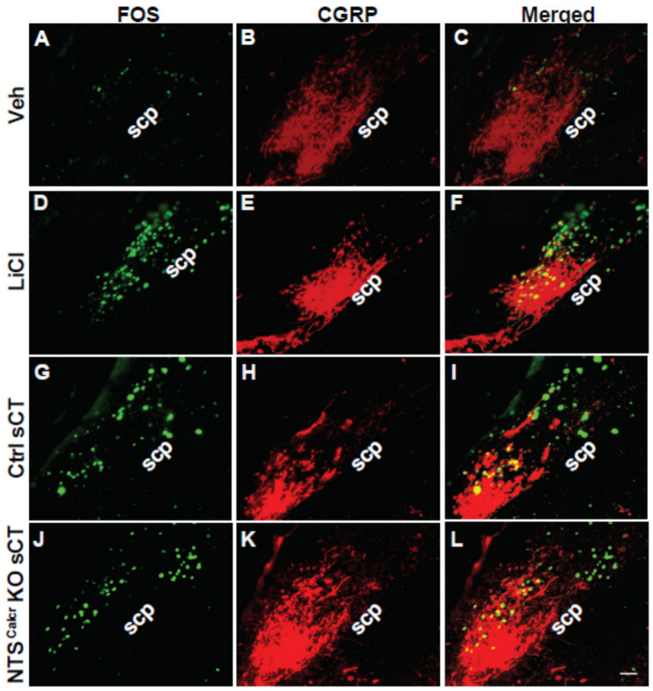

Supplemental Figure 5 (Related to Figure 5): CCK^{NTS} neurons, but not Calcr^{NTS} neurons, project to the CGRP-containing PBN region (a) and Colocalization of sCT-induced FOS with CGRP-IR in the PBN (b). (a: A-F) Representative images of hM3Dq (mCherry, red, left panels), CGRP (green, middle panels) and merged images (right panel) in the PBN of Calcr^{NTS}-Dq (A-C) and CCK^{NTS}-Dq mice (D-F). (**a: G-L**) Representative images of hM3Dq (mCherry, purple, left panels), GFP (green, middle panels) and merged images (right panel) in the PBN of Calcr^{NTS}-Dq/*Calca*$^{cre\text{-}GFP}$ (G-I) and CCK^{NTS}-Dq/*Calca*$^{cre\text{-}GFP}$ mice (J-L). Insets show digital zooms of the boxed area. (**a: M-R**) Representative images of Syn-mCherry (purple, left panels), GFP (green, middle panels) and merged images (right panel) in the PBN of *Calcr*^{cre}/*Calca*$^{cre\text{-}GFP}$ (M-O) and *Cck*^{cre}/*Calca*$^{cre\text{-}GFP}$ mice (P-R) following intra-NTS injection with Ad-Syn-mCherry. Insets show digital zooms of the boxed area. Images are representative of 3 per group. Scale bar equals 150 μm; scp=superior cerebellar peduncle. **(b: A-L)** Shown are representative images of FOS (green, left panels), CGRP (red, middle panels), and merged images (right) from mice treated with vehicle (Veh, **b: A-C**), LiCl (**b: D-F**), or with sCT (150 μg/kg,**b: G-L**) in control (Ctrl, A-I) or Calcr^{NTS}KO mice (**b: J-L**); representative of n=3 per group. Scale bar equals 75 μm; scp=superior cerebellar peduncle.

Supplemental Figure 6

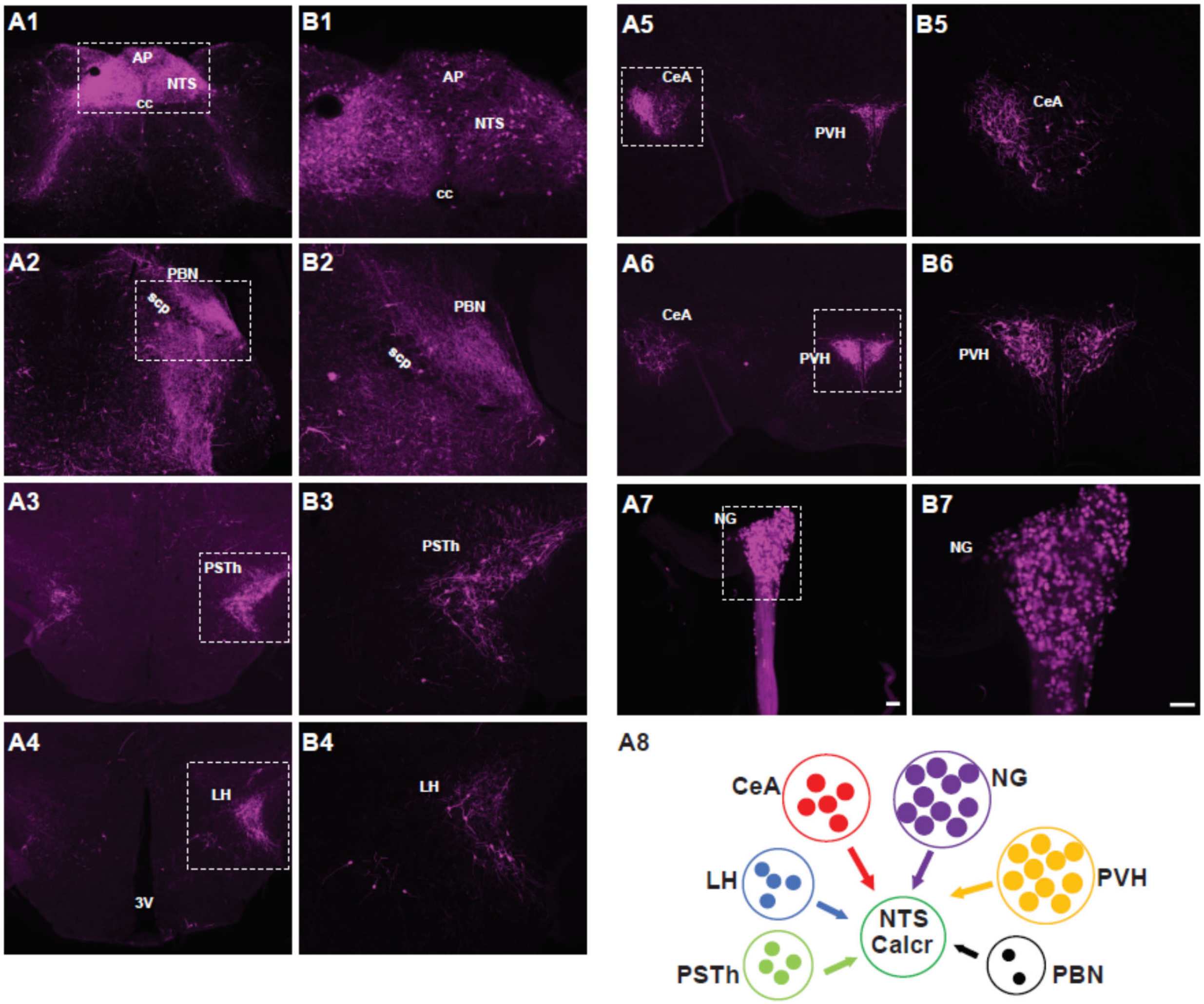

Supplemental Figure 6 (Related to Figure 5): Single-synapse rabies tracing reveals inputs to Calcr^{NTS} neurons. AAV$^{Flex\text{-}TVA+G}$ was injected into the NTS of *Calcr^{cre}* mice; 1 month later the mice were injected with pseudotyped defective mCherry-expressing rabies virus. Brains were collected one week later and sectioned to identify mCherry-expressing afferents. Representative images of areas with substantial staining are shown. Images in A1-A7 were taken at 4x; B1-B7 show 10x images of the boxed regions. A8 shows a summary about the inputs that Calcr^{NTS} neurons received throughout the nervous system, with number of dots proportional to the number of rabies-infected neurons detected in each region. Scale bars equal 150 µm. cc- central canal, PBN- parabrachial nucleus, scp=superior cerebellar peduncle, PSTh-parasubthalamic nucleus, LH-lateral hypothalamus, CeA- The central nucleus of the amygdala, PVH-para ventricular hypothalamus.NG- nodose ganglion. 3V- third cerebral ventricle. Images are representative of at least 3 animals.

Supplemental Figure 7

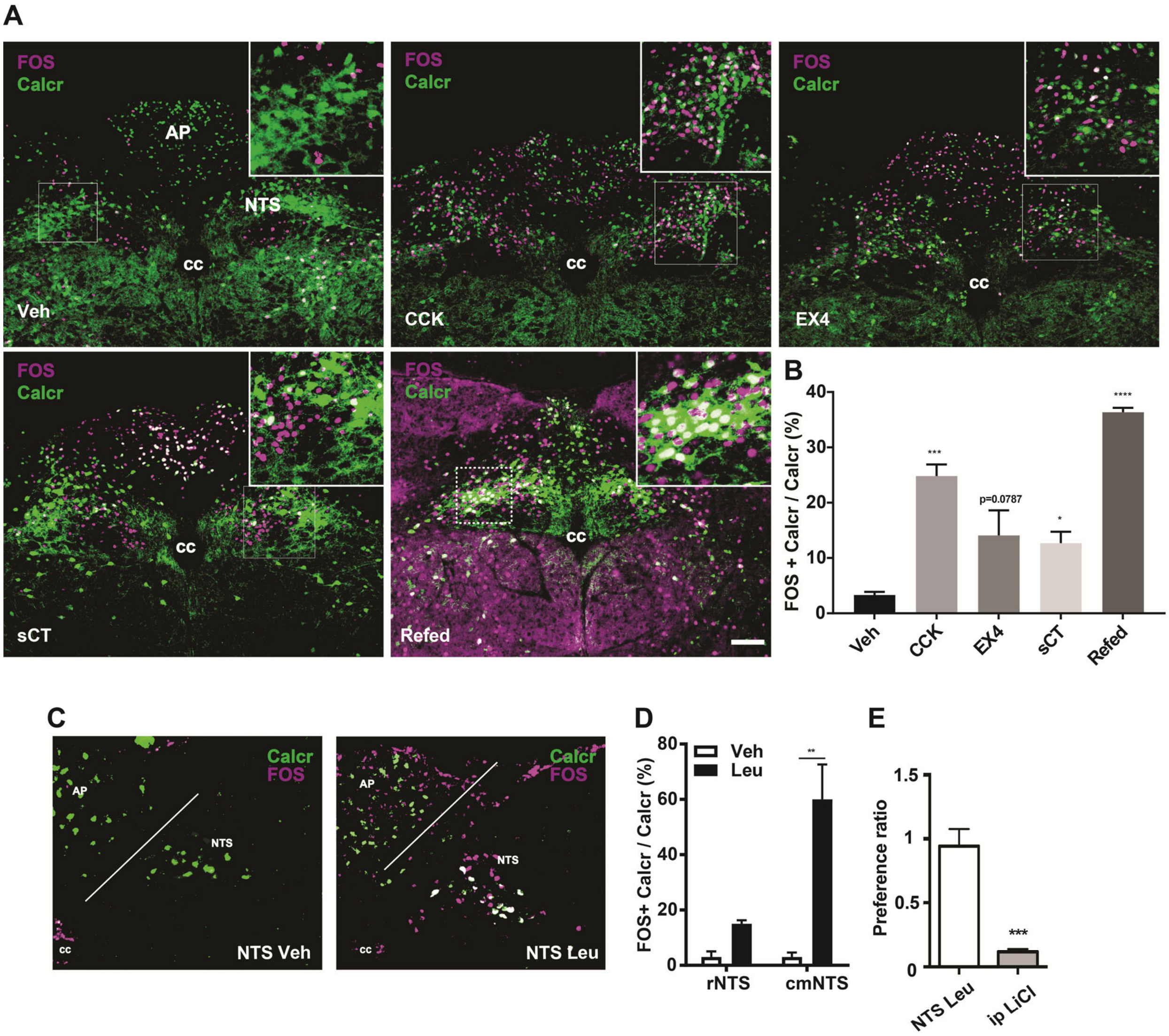

Supplemental Figure 7 (Related to Figure 6): Activation of Calcr^{NTS} neurons by gut peptide-mimetics and feeding, and NTS Leu activates Calcr^{NTS} cells non-aversively. (A) Representative NTS images from Calcr^{cre};tdTomato mice showing FOS (purple) and Calcr neurons (mCherry, green) two hours after injection with vehicle (Veh), CCK (IP, 100 µg/kg), EX4 (IP, 150 µg/kg), sCT (IP, 150 µg/kg), or refeeding with normal chow for 2 hours after an overnight fast, as indicated. cc- central canal. Scale bar equals 150 µm. (**B**) Calcr^{NTS} and FOS+Calcr^{NTS} neurons were counted and plotted as percent of total in the lower right panel. Mean +/- SEM is shown, n=3 per group. Mean +/- SEM is shown, unpaired t test was performed to compare each treatment group with Veh, *p<0.05, ***p<0.001, ****p<0.0001, or p value as noted**.** Images are representative of 3 per group; cc- central canal. Scale bar equals 150 µm. **(C)** Representative images the NTS in *Calcr^{cre}* mice stained for FOS (purple) and Calcr (green) following treatment with NTS vehicle (Veh, left panel) or NTS Leu (right panel). White line shows demarcation between AP and NTS. n=4 in each condition. (**D**)

Quantification of FOS-containing NTS Calcr cells from the rostral NTS (rNTS) and central medial NTS (cmNTS) from images such as shown in (C), n=4 in each group. (**E**) Quantification of CTA produced by NTS Leu I.C.V injection paired with saccharin, LiCl IP injected mice paired with saccharin were positive controls (n=6 in each group). Shown is mean +/-SEM. Unpaired t-test: **p<0.01, ***p<0.001.
