# Supplementary material for: Calcitonin Receptor Neurons in the Mouse Nucleus Tractus Solitarius Control Energy Balance via the Non-aversive Suppression of Feeding
Source: Cell Metab. 2020 Feb 4;31(2):301–312.e5. doi: 10.1016/j.cmet.2019.12.012 (PMC7104375; doi:10.1016/j.cmet.2019.12.012)
Supplement: Document S2. Article plus Supplemental Information [file mmc2.pdf]

Cell Metabolism

Article

# Calcitonin Receptor Neurons in the Mouse Nucleus Tractus Solitarius Control Energy Balance via the Non-aversive Suppression of Feeding

## Graphical Abstract

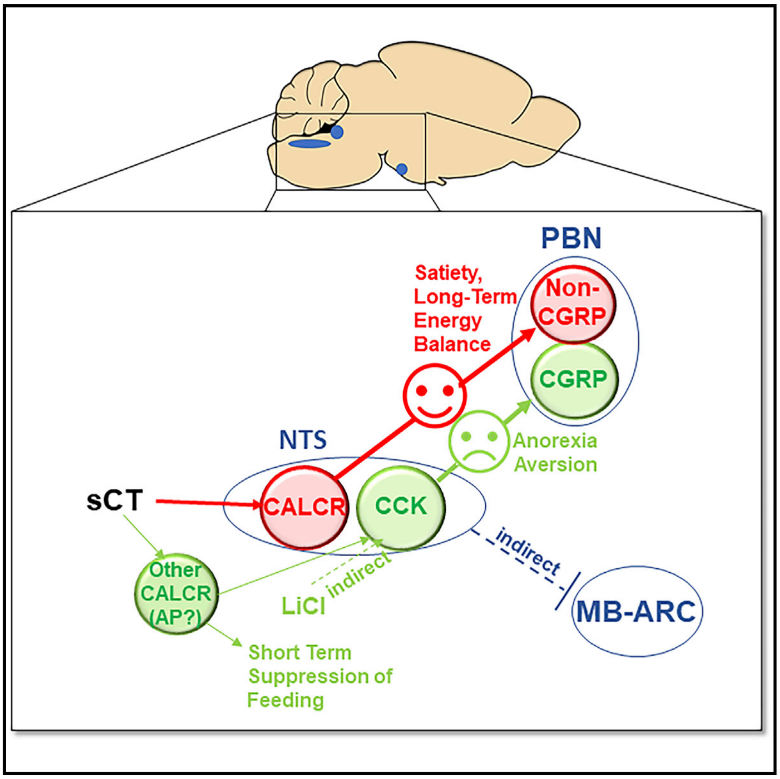


## Highlights

- NTS *Calcr* mediates food intake suppression but not aversive responses to sCT
- Activating NTS Calcr neurons non-aversively suppresses feeding
- These neurons act via non-CGRP PBN neurons
- These neurons control long-term energy balance, not just short-term feeding

## Authors

Wenwen Cheng, Ian Gonzalez, Warren Pan, ..., David P. Olson, Clemence Blouet, Martin G. Myers, Jr.

## Correspondence

csb69@medschl.cam.ac.uk (C.B.), mgmyers@umich.edu (M.G.M.)

## In Brief

While the hindbrain is often postulated to control only short-term parameters of feeding via circuits that mediate aversive responses when activated strongly, Cheng et al. have identified a hindbrain system that participates in the physiological control of energy balance and suppresses food intake without activating aversive systems or symptoms.

Cheng et al., 2020, Cell Metabolism *31*, 301–312
February 4, 2020 © 2019 Elsevier Inc.
https://doi.org/10.1016/j.cmet.2019.12.012

CellPress

CellPress

# Calcitonin Receptor Neurons in the Mouse Nucleus Tractus Solitarius Control Energy Balance via the Non-aversive Suppression of Feeding

Wenwen Cheng,[1] Ian Gonzalez,[2,3] Warren Pan,[1,4] Anthony H. Tsang,[5] Jessica Adams,[2] Ermelinda Ndoka,[1] Desiree Gordian,[1] Basma Khoury,[6] Karen Roelofs,[6] Simon S. Evers,[6] Andrew MacKinnon,[1] Shuangcheng Wu,[3] Henriette Frikke-Schmidt,[6] Jonathan N. Flak,[1] James L. Trevaskis,[7] Christopher J. Rhodes,[7] So-ichiro Fukada,[8] Randy J. Seeley,[6] Darleen A. Sandoval,[3,6] David P. Olson,[2,3] Clemence Blouet,[5,*] and Martin G. Myers, Jr.[1,2,3,4,9,*]
[1]Department of Internal Medicine, University of Michigan, Ann Arbor, MI 48105, USA
[2]Division of Endocrinology, Department of Pediatrics and Communicable Diseases, University of Michigan, Ann Arbor, MI 48105, USA
[3]Department of Molecular and Integrative Physiology, University of Michigan, Ann Arbor, MI 48105, USA
[4]Graduate Program in Cellular and Molecular Biology, University of Michigan, Ann Arbor, MI 48105, USA
[5]MRC Metabolic Diseases Unit, University of Cambridge Metabolic Research Laboratories, WT-MRC Institute of Metabolic Science, University of Cambridge, Cambridge CB2 0QQ, UK
[6]Department of Surgery, University of Michigan, Ann Arbor, MI 48105, USA
[7]Cardiovascular, Renal and Metabolic Diseases, AstraZenica LLC, Gaithersburg, MD 20878, USA
[8]Laboratory of Molecular and Cellular Physiology, Osaka University, Osaka 565-0871, Japan
[9]Lead Contact
*Correspondence: csb69@medschl.cam.ac.uk (C.B.), mgmyers@umich.edu (M.G.M.)
https://doi.org/10.1016/j.cmet.2019.12.012

## SUMMARY

To understand hindbrain pathways involved in the control of food intake, we examined roles for calcitonin receptor (CALCR)-containing neurons in the NTS. Ablation of NTS *Calcr* abrogated the long-term suppression of food intake, but not aversive responses, by CALCR agonists. Similarly, activating Calcr$^{NTS}$ neurons decreased food intake and body weight but (unlike neighboring Cck$^{NTS}$ cells) failed to promote aversion, revealing that Calcr$^{NTS}$ neurons mediate a non-aversive suppression of food intake. While both Calcr$^{NTS}$ and Cck$^{NTS}$ neurons decreased feeding via projections to the PBN, Cck$^{NTS}$ cells activated aversive CGRP$^{PBN}$ cells while Calcr$^{NTS}$ cells activated distinct non-CGRP PBN cells. Hence, Calcr$^{NTS}$ cells suppress feeding via non-aversive, non-CGRP PBN targets. Additionally, silencing Calcr$^{NTS}$ cells blunted food intake suppression by gut peptides and nutrients, increasing food intake and promoting obesity. Hence, Calcr$^{NTS}$ neurons define a hindbrain system that participates in physiological energy balance and suppresses food intake without activating aversive systems.

## INTRODUCTION

Obesity affects over one-third of the adult population in developed countries, leading to diabetes, cardiovascular disease, and other conditions that cause substantial morbidity and mortality (https://www.cdc.gov/obesity/data/adult.html). Unfortunately, most current medical therapies are ineffective in treating obesity (Vasanth Rao et al., 2019). Agents that mimic the action of gut peptides (such as agonists [including calcitonin and amylin] for the calcitonin receptor [CALCR] and glucagon-like peptide-1 receptor [GLP1R]) suppress long-term food intake and promote significant weight loss, though (Adams et al., 2018; Mietlicki-Baase et al., 2013; Secher et al., 2014; Lutz et al., 1995; Yamaguchi et al., 2015; Fujioka, 2015; Ladenheim, 2015). Thus, it is important to understand the neural mechanisms by which such peptides mediate their anorexic effects.

*Calcr* is widely distributed in the brain, including in several areas linked to food intake, including the hypothalamic arcuate nucleus (ARC), the paraventricular hypothalamic nucleus (PVH), and the amygdala (Becskei et al., 2004). The nucleus

**Context and Significance**

The most primitive portion of the brain, known as the hindbrain, has circuits that are widely thought to mediate only the short-term suppression of feeding by gut signals under normal physiologic conditions. These hindbrain circuits have been generally thought to suppress feeding through nausea and other aversive responses when activated strongly, as by drugs that mimic gut peptides. However, researchers at the University of Michigan identified a hindbrain system that not only participates in the physiological control of long-term energy balance, but also strongly and durably suppresses food intake without activating aversive systems or symptoms. In addition to revising our concept of food intake control by the hindbrain, these findings identify a circuit that represents a potentially ideal target for the treatment of obesity.

tractus solitarius (NTS; a CNS region crucial for integrating gut-derived prandial signals and promoting meal termination) (Beutler et al., 2017; Blouet and Schwartz, 2012; D'Agostino et al., 2016; Grill and Hayes, 2009, 2012; Hayes et al., 2011, 2016a; Hayes et al., 2010, 2016b) also contains *Calcr* cells (Calcr$^{NTS}$ neurons). Importantly, while agonists for CALCR (and other gut peptide receptors) decrease food intake, they also produce aversive responses that mimic gut malaise (Adams et al., 2018; Halatchev and Cone, 2005; Schier et al., 2012; Verbaeys et al., 2008), potentially limiting their therapeutic utility.

One line of thinking holds that meal-terminating brainstem circuits must also mediate aversive responses, such that overfeeding (and other gut-malaise-associated stimuli) would promote aversive signals by more strongly activating these circuits than would a normal-sized meal. Indeed, previous work has demonstrated that calcitonin gene-related peptide (CGRP)-containing neurons of the parabrachial nucleus (PBN; CGRP$^{PBN}$ cells) mediate aversion (as well as anorexia) in response to a variety of cues of gastrointestinal malaise (including that induced by intraperitoneal LiCl) and that silencing CGRP$^{PBN}$ cells increases meal size (Campos et al., 2016; Carter et al., 2015; Carter et al., 2013). For instance, stimulating PBN projections from *Cck*-expressing NTS (Cck$^{NTS}$) neurons activates CGRP$^{PBN}$ cells and promotes aversion and anorexia (Roman et al., 2017).

Normal food ingestion (or nutrient infusion into the gut) mediates positive reinforcement even while stimulating meal termination (Sclafani, 2013; Sclafani and Ackroff, 2012). Therefore, the circuits that terminate normal feeding must differ at least in part from those that convey aversive signals in response to gut malaise, and it should be possible to identify populations of NTS neurons that suppress food intake without activating CGRP$^{PBN}$ neurons or causing aversion.

While gut-peptide-responsive cells in the NTS (and in the adjacent area postrema [AP]) play prominent roles in meal termination and the control of meal size (Grill and Hayes, 2012), the gut peptide receptors on these cells only modestly (if at all) impact the long-term physiologic control of food intake and energy balance (Blouet and Schwartz, 2012). Hence, conventional wisdom holds that while NTS cells play important roles in the suppression of feeding in response to gut peptide receptor agonism and the control of meal termination, these NTS neurons do not participate in the physiologic regulation of long-term energy balance. This idea has not been tested directly by interfering with the overall function of NTS neurons, though. Thus, in addition to understanding the roles for NTS cells in the anorectic response to pharmacologic CALCR agonism and defining hindbrain systems that mediate the non-aversive suppression of food intake, it will be important to directly determine roles for NTS cells in the physiologic control of food intake and energy homeostasis. Here, we examined roles for Calcr$^{NTS}$ cells, demonstrating their role in mediating the non-aversive suppression of food intake and revealing their importance for the long-term control of energy balance.

## RESULTS

### Attenuation of the Anorectic, but Not Aversive, Response to CALCR Agonism in Calcr$^{NTS}$KO Mice

We sought to understand the roles for specific *Calcr*-expressing neurons for the control of food intake. Based upon *Calcr* expression patterns in the hypothalamus, we crossed *Calcr$^{flox}$* (Yamaguchi et al., 2015) onto the *Sim1$^{cre}$* (Balthasar et al., 2005) or *Lepr$^{cre}$* (Patterson et al., 2011) backgrounds (Calcr$^{Sim1}$KO [knockout] and Calcr$^{LepRb}$KO mice, respectively) (Figure S1A) to ablate *Calcr* in neurons of the PVH and in leptin receptor (LepRb) neurons (primarily neuropeptide Y-, agouti-related peptide-, and gamma-aminobutyric-acid-containing (NAG) cells of the ARC) (Pan et al., 2018). We also injected AAV$^{cre-mCherry}$ or AAV$^{GFP}$ (control) into the NTS of *Calcr$^{flox/flox}$* mice to ablate *Calcr* expression in this brain region (Calcr$^{NTS}$KO mice) (Figure 1A). We examined AAV reporter expression in the brains of all injected animals following the completion of experiments to ensure correct targeting (Figure 1B).

As expected, Calcr$^{NTS}$KO mice exhibited reduced salmon calcitonin (sCT)-stimulated NTS FOS-immunoreactivity (IR) (Figures 1C, 1D, and S1F). In contrast, Calcr$^{LepRb}$KO mice exhibited unchanged sCT-stimulated FOS-IR in the ARC (Figures S1B and S1C), and we detected increased sCT-stimulated FOS in the PVH of Calcr$^{Sim1}$KO mice despite decreased PVH *Calcr* expression in Calcr$^{Sim1}$KO mice (Figures S1D and S1E). These findings suggest that most sCT-stimulated FOS in the ARC and PVH is mediated indirectly (by other CALCR cells) and that PVH CALCR may suppress the sCT-dependent activation of PVH neurons by other CALCR-expressing cells.

Our analysis revealed no alteration in food intake, body weight, or body composition in Calcr$^{Sim1}$KO, Calcr$^{LepRb}$KO, and Calcr$^{NTS}$KO mice (Figures S2A–S2I). Hence, *Calcr* expression in the PVH, the NTS, and in ARC LepRb neurons does not meaningfully contribute to long-term energy balance under normal conditions.

To determine the anorectic response of these KO animals to CALCR agonists, we examined feeding following sCT administration. We found that sCT decreased food intake by >40% during the first 4 h of the dark cycle and that 3 days of twice-daily sCT administration suppressed food intake and lowered body weight similarly in Calcr$^{Sim1}$KO and Calcr$^{LepRb}$KO mice and their controls (Figures S2J–S2O). Thus, *Calcr* expression in PVH Sim1 neurons and hypothalamic LepRb neurons is not required for the suppression of food intake by sCT.

While we found that sCT suppressed food intake normally in Calcr$^{NTS}$KO mice for the first 2 h at the onset of the dark cycle, the anorectic effect of sCT in Calcr$^{NTS}$KO mice attenuated by 4 h, and sCT failed to suppress 24 h food intake and reduce body weight during 3 days of sCT administration in these animals (Figures 1E–1G). Indeed, 3-day treatment with sCT promoted body weight gain ($p = 0.047$) in the Calcr$^{NTS}$KO mice. Thus, not only are NTS *Calcr* (Calcr$^{NTS}$) neurons crucial for the anorectic response to sCT, but an unidentified set of *Calcr* neurons elsewhere in the brain may mediate an orexigenic signal that is normally overcome by the anorexic signal from the Calcr$^{NTS}$ cells.

In contrast to the reduced efficacy of sCT and davalintide (a co-agonist for the CALCR and amylin receptor [Mack et al., 2010]) on food intake suppression in Calcr$^{NTS}$KO mice (Figures 1E–1J), Calcr$^{NTS}$KO and control mice responded similarly to the NPY2R agonist, PYY3-36, and lipopolysaccharide (LPS) (Figures S2P–S2R), consistent with the specificity of NTS *Calcr* for the anorexic response to CALCR agonists. Overall, these data suggest that while NTS *Calcr* is not required for the control of

CellPress

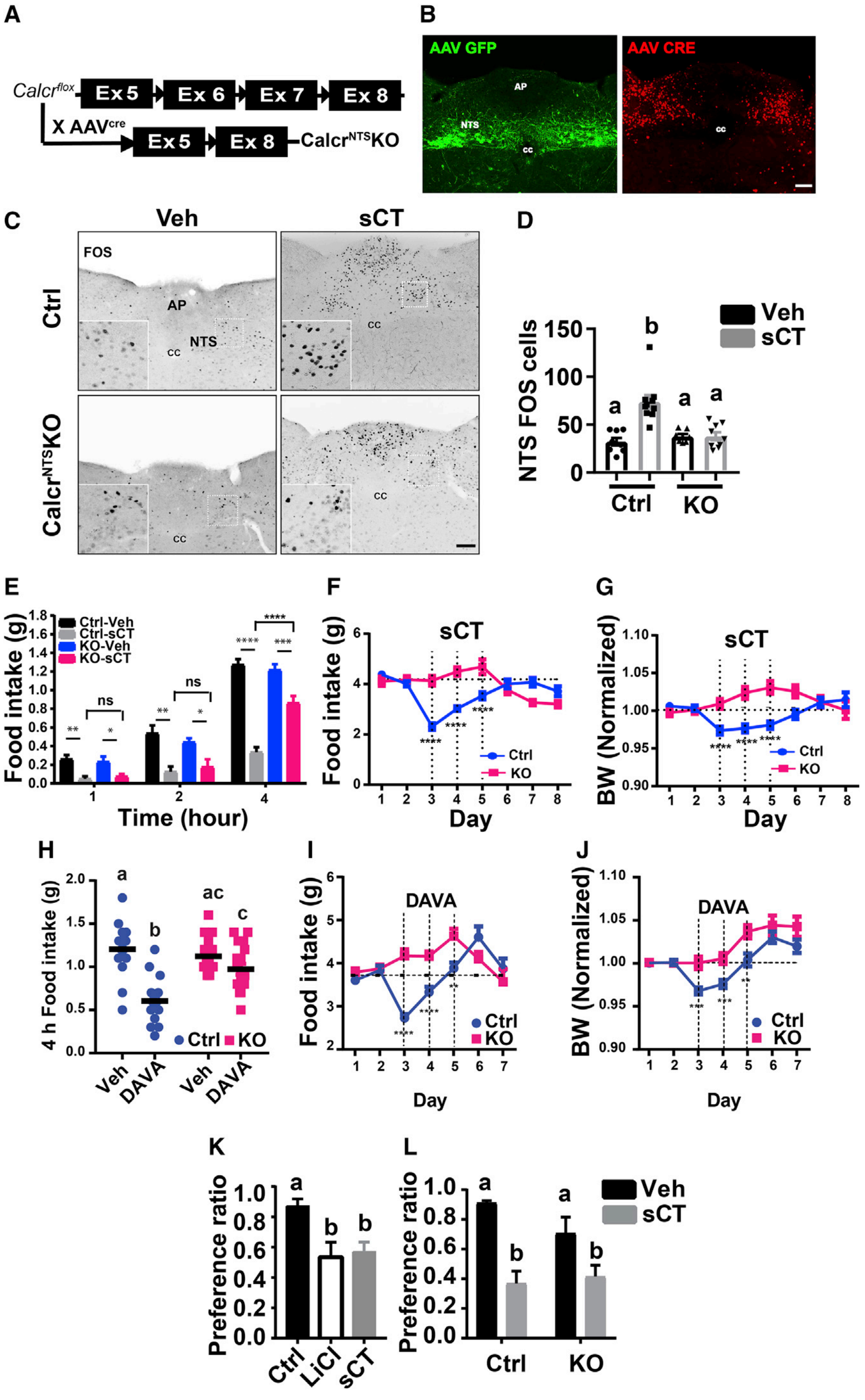

**Figure 1. Deletion of *Calcr* in Calcr$^{NTS}$ Neurons Prevents Food Intake Suppression but Not CTA Formation to sCT Treatment**

(A) Schematic diagram showing intra-NTS AAV$^{cre}$ delivery in *Calcr$^{flox}$* mice to generate Calcr$^{NTS}$KO mice.

(B) Representative images show AAV$^{GFP}$ (GFP, green, left panel) and AAV$^{cre}$ (mCherry, red, right panel) expression in the NTS of control and Calcr$^{NTS}$KO mice. Scale bar, 150 μm.

(C) Representative images showing FOS-IR (black) in vehicle- (Veh) and sCT- (IP, 150 μg/kg, 2 h) injected control and Calcr$^{NTS}$KO mice. Scale bar, 150 μm; cc, central canal.

(D) Quantification of FOS-IR cells in the NTS of mice treated as in (C). Shown is mean ± SEM; n = 4–10 per group.

(E) Control and Calcr$^{NTS}$KO mice were treated with Veh or sCT (150 μg/kg, IP) and food intake was measured for the subsequent 4 h. n = 8 in 1-h and 2-h groups, n = 26 in 4-h groups.

(F and G) Daily food intake (F) and body weight (G) were measured during 2 days of vehicle, 3 days of sCT (IP, 150 μg/kg, BID), and 3 additional days of vehicle injection. Food intake and body weight were normalized to baseline. n = 18 in control group, n = 8–10 in Calcr$^{NTS}$KO group.

(H–J) Control (Ctrl, blue) and Calcr$^{NTS}$KO (KO, red) mice were treated with Davalintide (560 μg/kg, IP, twice daily) and food intake was measured for the first 4 h of the dark cycle (H) or food intake (I) and body weight (J) was measured daily over 3 days of injections (dashed lines); n = 18 per group.

(K and L) Conditioned taste aversion (CTA) was determined following the IP injection of Veh, LiCl (126 mg/kg), or sCT (150 μg/kg) in control (K) or control and Calcr$^{NTS}$KO mice (L); n = 7–10 per group.

All graphs: Mean ± SEM is shown. One-way ANOVA, Tukey's multiple comparisons was performed in (D), (H), (K), and (L), different letters indicate differences between groups, $p < 0.05$. The same test was performed for each time point in (E); ns, not significant. Two-way ANOVA, Sidak's multiple comparisons test was performed for (F, G, I, and J). *$p < 0.05$, **$p < 0.01$, ***$p < 0.001$, ****$p < 0.0001$.

energy balance at baseline, *Calcr* in the NTS mediates the long-term effects of CALCR agonists on food intake and body weight.

To understand the potential role for Calcr$^{NTS}$ neurons in the aversive response to sCT, we utilized the conditioned taste aversion (CTA) assay, in which pairing of a stimulus with exposure to a novel tastant (e.g., grape flavor or 0.15% saccharine in drinking water, or high-fat diet [HFD]) inhibits the subsequent consumption of aversive stimulus-paired tastants. Like LiCl, sCT promotes a CTA in normal mice (Freed et al., 1981) (Figure 1K). Interestingly, although deletion of NTS *Calcr* abrogated the ability of sCT to reduce food intake (Figures 1E–1J), sCT treatment promoted a CTA in Calcr$^{NTS}$KO mice similarly to control animals (Figure 1L), revealing that NTS *Calcr* is not required for the formation of a CTA to sCT, thus suggesting that Calcr$^{NTS}$ cells might suppress food intake without participating in aversive responses.

## Calcr$^{NTS}$ Neurons Non-aversively Decrease Food Intake and Body Weight

To understand the relationship of Calcr$^{NTS}$ neurons to previously studied populations of NTS neurons, we examined the potential colocalization of Calcr$^{NTS}$ cells with NTS neurons that express LepRb, cholecystokinin (Cck), or tyrosine hydroxylase (Th) (Figures S3A–S3D). While LepRb$^{NTS}$ and Cck$^{NTS}$ were distinct

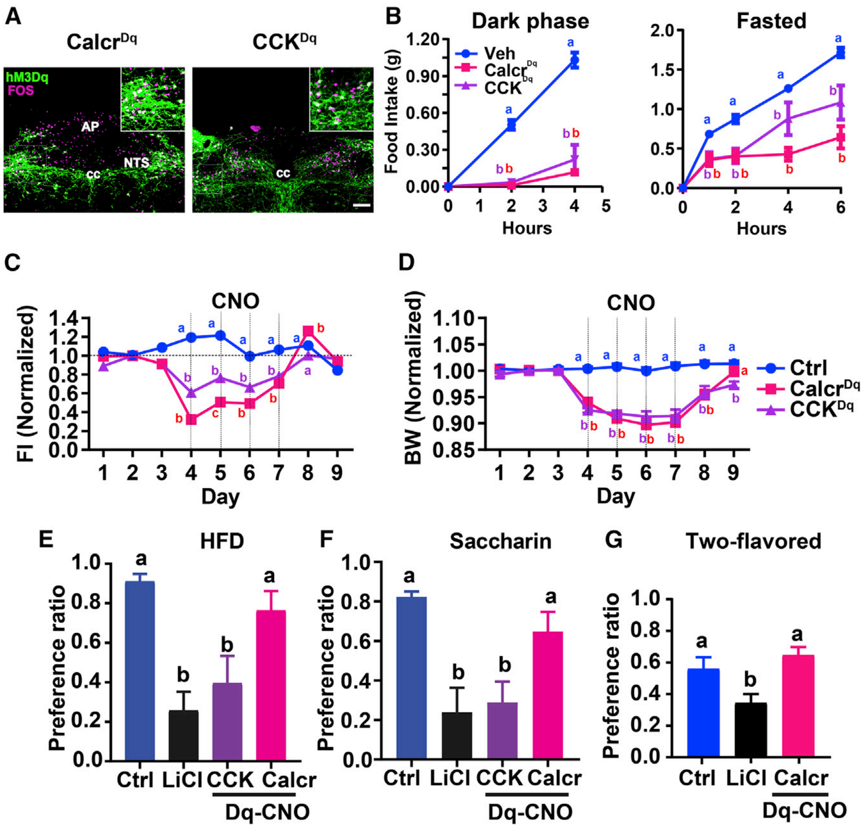


**Figure 2. Effects of DREADD-Mediated NTS Neuron Activation on Food Intake and Body Weight, and CTA Induction**

(A) Representative images showing FOS (purple) and hM3Dq (green) 2 h after CNO injection (1 mg/kg, IP) in Calcr$^{NTS}$-Dq (Calcr$^{Dq}$) and Cck$^{NTS}$-Dq (CCK$^{Dq}$) mice. Scale bar, 150 μm; cc, central canal.

(B) Food (chow) intake during CNO (1 mg/kg, IP) treatment of control (n = 23–25), Calcr$^{Dq}$ (n = 7–20), and CCK$^{Dq}$ (n = 5–9) mice during the first 4 h of the dark cycle (left panel) and the first 6 h following re-feeding in overnight fasted mice (right panel).

(C and D) Daily food intake (C) and body weight (D) were measured during 3 days of vehicle, 4 days of CNO (IP, 1 mg/kg, BID), and 2 additional days of vehicle injection. Food intake and body weight were normalized to baseline. Control: n = 12; Calcr$^{Dq}$: n = 12; and CCK$^{Dq}$: n = 7.

(E–G) CTA was determined for control, Calcr$^{hm3dq}$, and CCK$^{hm3dq}$ groups in which stimulus was paired with HFD (E, n = 10, 10, 11, and 9 for control, LiCl, CCK$^{Dq}$, and Calcr$^{Dq}$ groups, respectively), Saccharin (F, n = 13, 11, 8, and 12 for control, LiCl, CCK$^{Dq}$, and Calcr$^{Dq}$ groups, respectively) or one of two flavors (G, n = 11, 12, and 8 for control, LiCl, and Calcr$^{Dq}$ groups, respectively).

All graphs: Mean ± SEM is shown. Two-way ANOVA, Sidak's multiple comparisons test for (B), (C), and (D). One-way ANOVA, Tukey's multiple comparisons was performed in (E), (F), and (G). Different letters indicate difference between groups, $p < 0.05$.

from Calcr$^{NTS}$ cells, a significant proportion of Th$^{NTS}$ cells contain *Calcr* (and vice versa); Th$^{NTS}$ cells do not overlap significantly with Cck$^{NTS}$ cells.

Because the chemogenetic activation of Cck$^{NTS}$ cells has been shown to promote the aversive suppression of food intake (Roman et al., 2017), we utilized these cells as comparators for Calcr$^{NTS}$ neurons (Figure 2). We bilaterally injected AAV$^{Flex-hM3Dq}$ into the NTS of *Calcr$^{cre}$* or *Cck$^{cre}$* mice to cre-dependently express the Gq-coupled (activating; hM3Dq) designer receptor exclusively activated by designer drugs (DREADD) in Calcr$^{NTS}$ and Cck$^{NTS}$ cells, permitting their activation by the injection of clozapine-N-oxide (CNO) (Smith et al., 2016; Zhu and Roth, 2014). We used the post hoc detection of mCherry (which is fused to hM3Dq in AAV$^{Flex-hM3Dq}$) in these Calcr$^{NTS}$-Dq and Cck$^{NTS}$-Dq mice to ensure that we analyzed only mice with NTS-restricted hM3Dq expression (Figure 2A).

CNO treatment promoted FOS-IR in mCherry-expressing cells in the NTS of Calcr$^{NTS}$-Dq and Cck$^{NTS}$-Dq mice, consistent with the DREADD-mediated activation of Calcr$^{NTS}$ and Cck$^{NTS}$ cells, respectively, in these animals (Figure 2A). The activation of Calcr$^{NTS}$ or Cck$^{NTS}$ cells at the onset of the dark cycle or following an overnight fast completely abrogated the intake of normal chow for 2 h and continued to dramatically reduce food intake for several hours (Figures S3E and S3F), although food intake suppression was less robust in Cck$^{NTS}$-Dq mice than in Calcr$^{NTS}$-Dq animals by 4 h after refeeding in fasted animals (Figure 2B). Furthermore, the twice-daily injection of CNO reduced food intake by 70% for the first 24 h and then by approximately 50% thereafter in Calcr$^{NTS}$-Dq mice (Figure 2C). CNO reduced long-term food intake less dramatically in Cck$^{NTS}$-Dq mice, but both mouse lines lost approximately 10% of their body weight over the 4-day treatment (Figures 2C and 2D). Thus, both Calcr$^{NTS}$ and Cck$^{NTS}$ cells suppress food intake and body weight, although Calcr$^{NTS}$ cells mediate a greater long-term suppression of feeding than Cck$^{NTS}$ cells.

Interestingly, despite the rapid and complete suppression of short-term food intake by both Calcr$^{NTS}$ and Cck$^{NTS}$ cells, only the activation of Cck$^{NTS}$ cells promoted a CTA (Figures 2E–2G). Furthermore, in the two-flavor choice paradigm, animals consumed more of the flavor that was paired with the activation of Calcr$^{NTS}$ cells (Figure S3G); the lack of significant change in the preference ratio likely reflects the lower sensitivity of this measure due to normalization for total volume consumed. Thus, not only is *Calcr* in NTS neurons crucial for the anorectic (but not aversive) response to sCT treatment, but the activation of Calcr$^{NTS}$ cells causes the acute and chronic suppression of food intake while promoting reinforcement rather than aversion.

Because of the overlap between Calcr$^{NTS}$ and Th$^{NTS}$ cells, we also examined the response to activating Th$^{NTS}$ cells (Figures S3H–S3L). While (like Calcr$^{NTS}$ cells) we found that Th$^{NTS}$ activation failed to promote a CTA, activating Th$^{NTS}$ cells only weakly suppressed food intake compared to Calcr$^{NTS}$ cells; thus, we continued to focus on Calcr$^{NTS}$ neurons to understand NTS cells that mediate the non-aversive suppression of food intake. The more robust suppression of food intake by Calcr$^{NTS}$ than Th$^{NTS}$ cells might reflect some property of the non-Th Calcr$^{NTS}$ cells that mediate a stronger anorectic response than Th$^{NTS}$ cells.

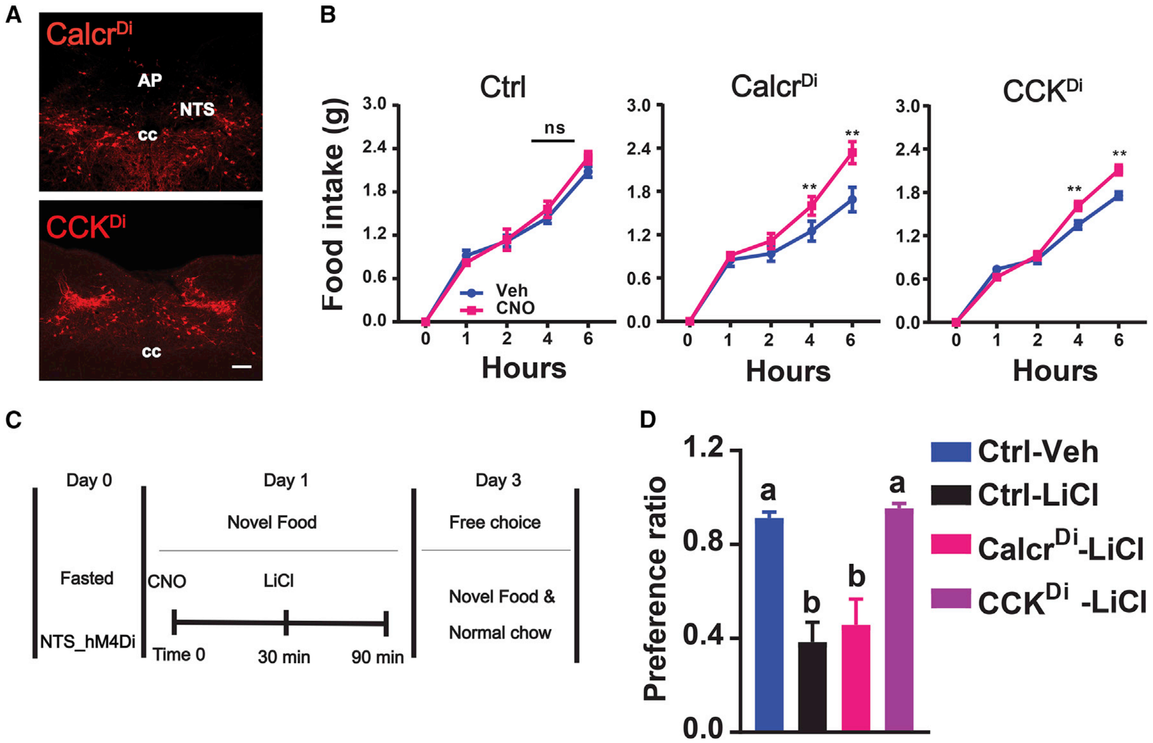


**Figure 3. Distinct Roles for Calcr$^{NTS}$ and Cck$^{NTS}$ Neurons in CTA Formation**

(A) Representative images showing hM4Di (red) in Calcr$^{NTS}$-Di (Calcr$^{Di}$) and Cck$^{NTS}$-Di (CCK$^{Di}$) mice. Scale bar, 150 μm; cc, central canal.

(B) Food intake for the first 6 h of refeeding following an overnight fast in the indicated groups of mice following treatment with vehicle (Veh) or CNO (IP, 1 mg/kg). Assay was performed using a crossover design with 1 week between conditions. Control: n = 5; Calcr$^{Di}$ and CCK$^{Di}$: n = 8 each.

(C) Schematic diagram showing the protocol for testing CTA acquisition to novel food (HFD) by LiCl injection paired with CNO-mediated (IP, 1 mg/kg) silencing of Calcr$^{NTS}$ or CCK$^{NTS}$ neurons.

(D) Quantification of CTA to HFD produced by LiCl injection paired with silencing Calcr$^{NTS}$ or CCK$^{NTS}$ neurons (n = 11 in vehicle and LiCl groups, n = 8 for in Calcr$^{Di}$ and CCK$^{Di}$ groups). Mean ± SEM is shown. Paired t test was performed for each time point in (B), **$p < 0.01$. One-way ANOVA, Tukey's multiple comparisons was performed in (D), different letters indicate difference between conditions, $p < 0.05$.

Although the known role for the NTS in the control of food intake is consistent with the notion that Calcr$^{NTS}$ and Cck$^{NTS}$ cells suppress food intake directly, it is also possible that the activation of these cells could promote other effects (e.g., pain) that indirectly blunt feeding. We thus bilaterally injected AAV$^{Flex-hM4Di}$ to express the Gi-coupled inhibitory hM4Di DREADD in Calcr$^{NTS}$ and Cck$^{NTS}$ cells (Calcr$^{NTS}$-Di and Cck$^{NTS}$-Di mice, respectively) (Figure 3A), permitting their acute inhibition by CNO. We found that CNO increased food intake following an overnight fast similarly in Calcr$^{NTS}$-Di and Cck$^{NTS}$-Di mice (Figure 3B). Furthermore, while the hM4Di-mediated inhibition of Calcr$^{NTS}$ cells did not alter CTA formation in response to LiCl administration, CNO administration blocked CTA formation in Cck$^{NTS}$-Di mice (Figures 3C and 3D). Thus, while Cck$^{NTS}$ cells provoke aversive responses while suppressing food intake and are required for CTA formation in response to LiCl, Calcr$^{NTS}$ cells similarly suppress food intake but are neither necessary nor sufficient to mediate a CTA.

## Downstream Targets of Calcr$^{NTS}$ and Cck$^{NTS}$ Neurons

Because Cck$^{NTS}$ cells promote CTA formation while Calcr$^{NTS}$ cells play no role in this process, these NTS cell types must act via different downstream circuits, at least in part. Nutritional signals from the gut suppress the activity of ARC NAG neurons (Beutler et al., 2017), presumably via a brainstem circuit. Because we found that the chemogenetic activation of Calcr$^{NTS}$ cells suppressed ghrelin-simulated food intake (which is largely mediated by the activation of NAG neurons) (Figure 4A), we tested the potential role for Calcr$^{NTS}$ cells in the inhibition of NAG cells. The fasting-induced activation of NAG cells can be monitored by the accumulation of FOS-IR in the medial basal ARC (MB-ARC; Figures 4B–4D) (Beutler et al., 2017; Olofsson et al., 2009); this fasting-induced MB-ARC activation can be decreased by refeeding. Similarly, the chemogenetic activation of Calcr$^{NTS}$ cells suppressed fasting-induced MB-ARC FOS-IR (Figures 4C and 4D). Thus, Calcr$^{NTS}$ cells inhibit fasting-induced MB-ARC FOS-IR cells, suggesting that hindbrain neurons control hypothalamic circuits crucial for the regulation of feeding. While the inhibition of MB-ARC cells may contribute to the suppression of food intake by Calcr$^{NTS}$ neurons, the activation of Cck$^{NTS}$ neurons also blunts fasting-induced MB-ARC FOS (Figures 4C and 4D). Hence, the difference in downstream signaling between Calcr$^{NTS}$ and Cck$^{NTS}$ cells must lie elsewhere.

To define potential differences in the food-intake-suppressing systems engaged by Calcr$^{NTS}$ and Cck$^{NTS}$ neurons, we identified their downstream projections by the cre-dependent

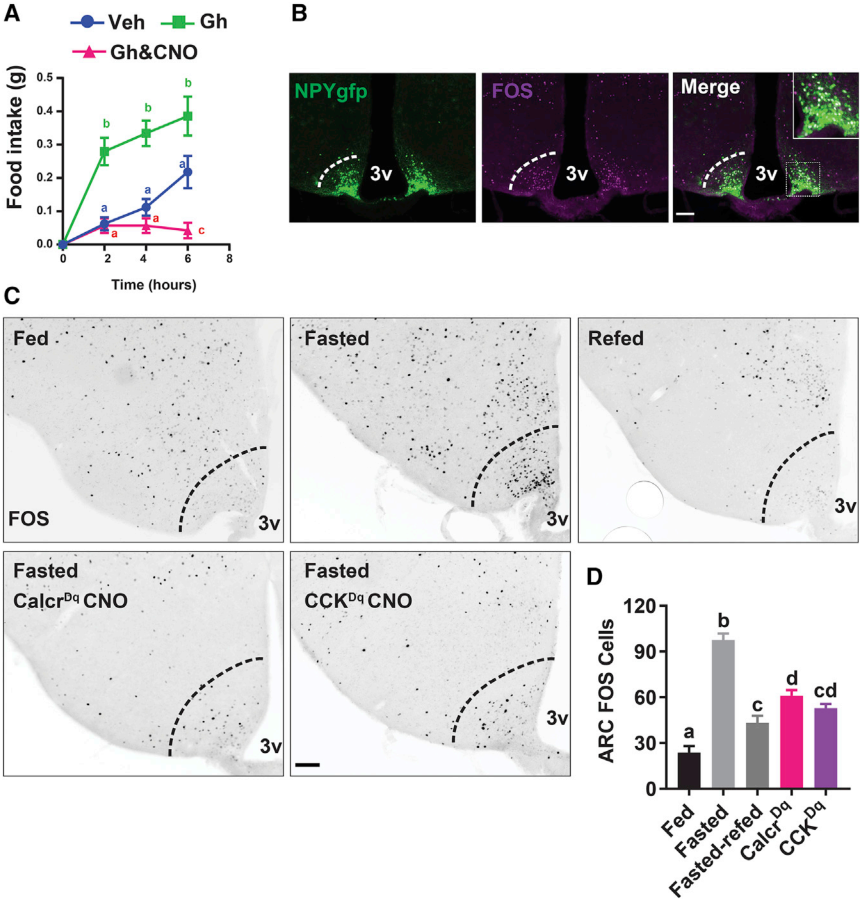


**Figure 4. Inhibition of Fasting-Induced MB-ARC FOS during Calcr$^{NTS}$ Neuron Activation**

(A) Calcr$^{NTS}$-Dq mice were treated with vehicle (Veh), Ghrelin (Gh, 400 μg/kg, IP), or Gh plus CNO (IP, 1 mg/kg) 3 h after the onset of the light cycle (9 AM) and food intake was monitored over the subsequent 6 h. Veh: n = 14, Gh and Gh+CNO: n = 12.

(B) Shown is a representative image of FOS-IR (purple) and GFP (green) in NPY$^{GFP}$ brain sections from overnight-fasted mice. Scale bar, 150 μm; 3V, third cerebral ventricle.

(C) Representative images showing ARC FOS (black) 3 h after the onset of the light cycle in *ad libitum*-fed (Fed), overnight fasted, overnight fasted and refed (Fast-refed), and overnight fasted with CNO (1 mg/kg, IP, 2 h prior to perfusion) in Calcr$^{Dq}$ and CCK$^{Dq}$ mice. Scale bar, 150 μm; 3V, third cerebral ventricle. Dashed line shows the limits of the MB-ARC region used for quantification.

(D) Quantification of ARC FOS for the groups shown in (C); n = 5, 6, 4, 9, and 5, respectively. Mean ± SEM is shown. Two-way ANOVA, Sidak's multiple comparisons test for (A). One-way ANOVA, Tukey's multiple comparisons was performed in (D). Different letters indicate difference between conditions, $p < 0.05$.

expression of a synaptophysin-mCherry (Syn-mCherry) fusion protein in these cells following the unilateral injection of AAV$^{Flex-Syn-mCherry}$ into the NTS of *Calcr$^{cre}$* and *Cck$^{cre}$* animals. This analysis revealed projections to the PBN, PVH, and dorsomedial hypothalamic nucleus (DMH) from both Calcr$^{NTS}$ and Cck$^{NTS}$ neurons (Figures S4A–S4H). Similarly, activation of Calcr$^{NTS}$ and Cck$^{NTS}$ cells each increased FOS-IR in the NTS, PBN, PVH, and DMH, although the Calcr$^{NTS}$ cells appeared to promote FOS-IR in the PVH and DMH more strongly than did Cck$^{NTS}$ cells (Figures S4I–S4T). Interestingly, while the distribution of projections and Dq-stimulated FOS-IR within the target regions was similar in most cases, Calcr$^{NTS}$ cells targeted a PBN region slightly more dorsal and medial to the region targeted by Cck$^{NTS}$ cells (Figures S4B, S4F, S4J, S4N, and S4R). Despite this difference in PBN projection targets, the optogenetic activation of PBN terminals from either Calcr$^{NTS}$ or Cck$^{NTS}$ neurons suppressed food intake at the onset of the dark cycle and following an overnight fast (Figures 5A–5C).

Because CGRP$^{PBN}$ cells contribute to the suppression of food intake during the activation of Cck$^{NTS}$ neurons and mediate aversive responses (Carter et al., 2015; Carter et al., 2013; Roman et al., 2016), we examined the potential innervation and regulation of CGRP$^{PBN}$ cells by Calcr$^{NTS}$ cells. In our initial experiments, we stained for hM3Dq-mCherry and CGRP (which identifies the region containing CGRP$^{PBN}$ cells but poorly reveals CGRP$^{PBN}$ soma) in Calcr$^{NTS}$-Dq and Cck$^{NTS}$-Dq mice (Figure S5). While Cck$^{NTS}$-Dq mice demonstrated substantial mCherry innervation of the CGRP-IR PBN field, Calcr$^{NTS}$-Dq mice exhibited little overlap between the main PBN projection field of Calcr$^{NTS}$ cells and CGRP$^{PBN}$ cells. Furthermore, sCT promoted FOS-IR in the CGRP$^{PBN}$ field in Calcr$^{NTS}$KO and control mice (Figure S5). Thus, unlike Cck$^{NTS}$ cells, it appears that Calcr$^{NTS}$ cells neither innervate CGRP$^{PBN}$ cells nor are required for the activation of CGRP$^{PBN}$ cells by sCT.

To directly examine the ability of Calcr$^{NTS}$ and Cck$^{NTS}$ cells to activate CGRP$^{PBN}$ cells, we bred *Calcr$^{cre}$* and *Cck$^{cre}$* onto the *Calca$^{cre-GFP}$* background (Carter et al., 2015), which permits the GFP-dependent detection of CGRP$^{PBN}$ cells. We injected AAV$^{Flex-hM3Dq}$ into the NTS of these mice and examined the distribution of innervation and FOS-IR within the PBN and its colocalization with GFP-labeled CGRP$^{PBN}$ cells (Figures 5D, 5E, and S5). This analysis confirmed the more dorsal medial distribution of projections and FOS-IR within the PBN following the activation of Calcr$^{NTS}$ neurons than for Cck$^{NTS}$ cells. Furthermore, although Cck$^{NTS}$ cells promoted strong FOS accumulation in CGRP$^{PBN}$ cells, Calcr$^{NTS}$ cells did not. Thus, Calcr$^{NTS}$ neurons suppress food intake via the PBN but (unlike Cck$^{NTS}$ cells) poorly activate aversive CGRP$^{PBN}$ cells, consistent with their non-aversive suppression of food intake. These data thus reveal the existence of a population of non-CGRP PBN neurons that mediates the non-aversive suppression of food intake by Calcr$^{NTS}$ cells.

## Calcr$^{NTS}$ Cells Mediate the Suppression of Food Intake by a Variety of Stimuli and Contribute to Long-Term Energy Balance

To understand the potential roles for Calcr$^{NTS}$ neurons in the control of food intake, we examined the afferent inputs to these cells by means of single-synapse retrograde tracing with

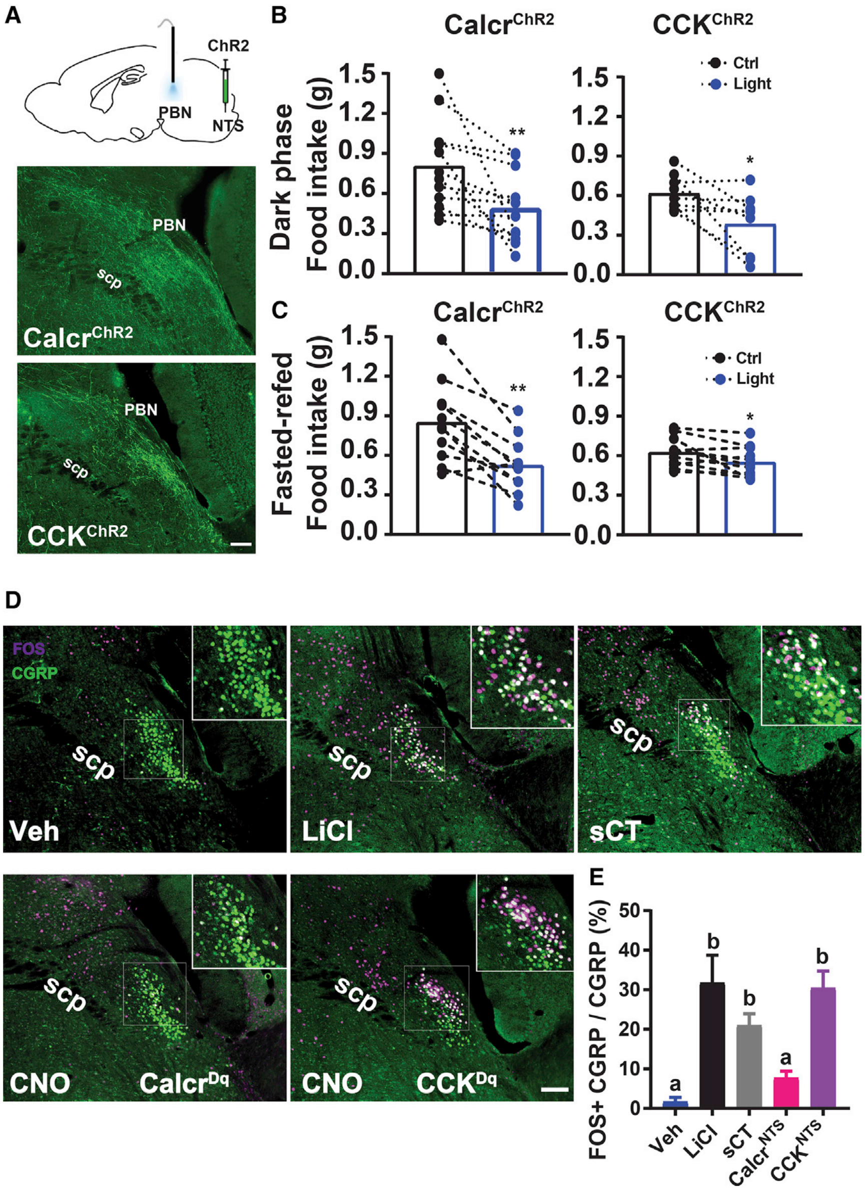


**Figure 5. Roles for PBN Projections from Calcr$^{NTS}$ and CCK$^{NTS}$ Neurons**

(A) Diagram showing experimental setup and images showing ChR2-GFP-containing projections (green) into the PBN of Calcr$^{NTS}$-ChR2 (Calcr$^{ChR2}$) and Cck$^{NTS}$-ChR2 (CCK$^{ChR2}$) mice. Scale bar, 150 μm; scp, superior cerebellar peduncle.

(B and C) Food intake over the first 2 h of the dark cycle (B) or during the first hour following the return of food to overnight-fasted mice (C) under control (Ctrl) or blue-light-stimulated (Light) conditions. Mean ± SEM is shown, paired t test was performed, $^{*}p < 0.05$, $^{**}p < 0.01$. Dark cycle: Calcr$^{ChR2}$ n = 12, CCK$^{ChR2}$ n = 9; refeeding: Calcr$^{ChR2}$ n = 12, CCK$^{ChR2}$ n = 11.

(D) Representative images of FOS (purple) and CGRP cells (GFP, green) in the PBN of mice on the *Calca*$^{cre-GFP}$ background 2 h after treatment with vehicle (Veh), sCT (150 μg/kg, IP), LiCl (126 mg/kg, IP), or CNO (1 mg/kg, IP) in Calcr$^{NTS}$-Dq (Calcr$^{Dq}$) and Cck$^{NTS}$-Dq (CCK$^{Dq}$) mice. Scale bar, 150 μm; scp, superior cerebellar peduncle.

(E) Quantification of FOS-positive CGRP$^{PBN}$ neurons from the groups in (D). Mean ± SEM is shown; n = 3 per group. One-way ANOVA, Tukey's multiple comparisons was performed, different letters indicate differences, $p < 0.05$.

defective rabies virus (Flak et al., 2017; Wickersham et al., 2007) (Figure S6). This analysis revealed strong inputs from the nodose ganglion and a variety of forebrain sites, including the PVH, lateral hypothalamic area (LHA), and central amygdala.

Because the innervation of Calcr$^{NTS}$ cells by vagal afferents in the nodose ganglion suggested that Calcr$^{NTS}$ cells receive input from the gut, we examined their activation by a variety of anorectic agonists for gut peptide receptors, including sCT, CCK, and exendin-4 (Ex4, a GLP1R agonist). Each of these peptides, as well as refeeding following a fast, increased FOS-IR in Calcr$^{NTS}$ cells, as well as in other NTS cells (Figures S7A and S7B). To examine roles for Calcr$^{NTS}$ cells in the anorectic response to these peptides, we bilaterally injected AVV$^{Flex-TetTox}$ (which mediates the cre-dependent expression of tetanus toxin [TetTox] to prevent synaptic neurotransmitter release [Bergey et al., 1987; Kim et al., 2009]) into the NTS of *Calcr*$^{cre}$ mice (Figure 6A). These Calcr$^{NTS}$-TT mice exhibited an attenuated suppression of food intake in response to sCT, as expected (Figure 6B). Furthermore, Calcr$^{NTS}$-TT mice also exhibit blunted suppression of food intake by CCK and Ex4 (Figures 6C and 6D). Thus, Calcr$^{NTS}$ neurons contribute to the anorectic response to a variety of food-intake-suppressing gut peptide receptor agonists.

We also examined the potential role for Calcr$^{NTS}$ cells in the strong appetite-suppressing effects of the amino acid Leu. Intra-NTS Leu (NTS-Leu) suppresses food intake (Blouet and Schwartz, 2012) without producing a CTA (Figure S7E). Indeed, we found that NTS-Leu promotes the activation of Calcr$^{NTS}$ cells (Figures S7C and S7D). Furthermore, NTS-Leu failed to suppress food intake in Calcr$^{NTS}$-TT mice, although NTS-Leu suppressed food intake normally in Cck$^{NTS}$-TT mice with silenced Cck$^{NTS}$ neurons (Figures 6E–6G).

To directly assess a potential role for Calcr$^{NTS}$ neurons in energy homeostasis, we examined food intake and body weight in Calcr$^{NTS}$-TT mice for 7 weeks on a chow diet, followed by an additional 6 weeks on HFD (Figure 7). We found that Calcr$^{NTS}$-TT mice exhibited a tendency toward increased body weight on a chow diet. Indeed, the continuous analysis of food intake over 24 h in these mice revealed increased nocturnal food intake, and we also observed increased cumulative food intake over 7 weeks of home cage chow feeding (Figures 7B and 7C).

Furthermore, exposure to HFD resulted in a 25%–30% increase in food intake by Calcr$^{NTS}$-TT mice compared to controls (Figure 7A) over the 6 weeks of HFD exposure, promoting an additional

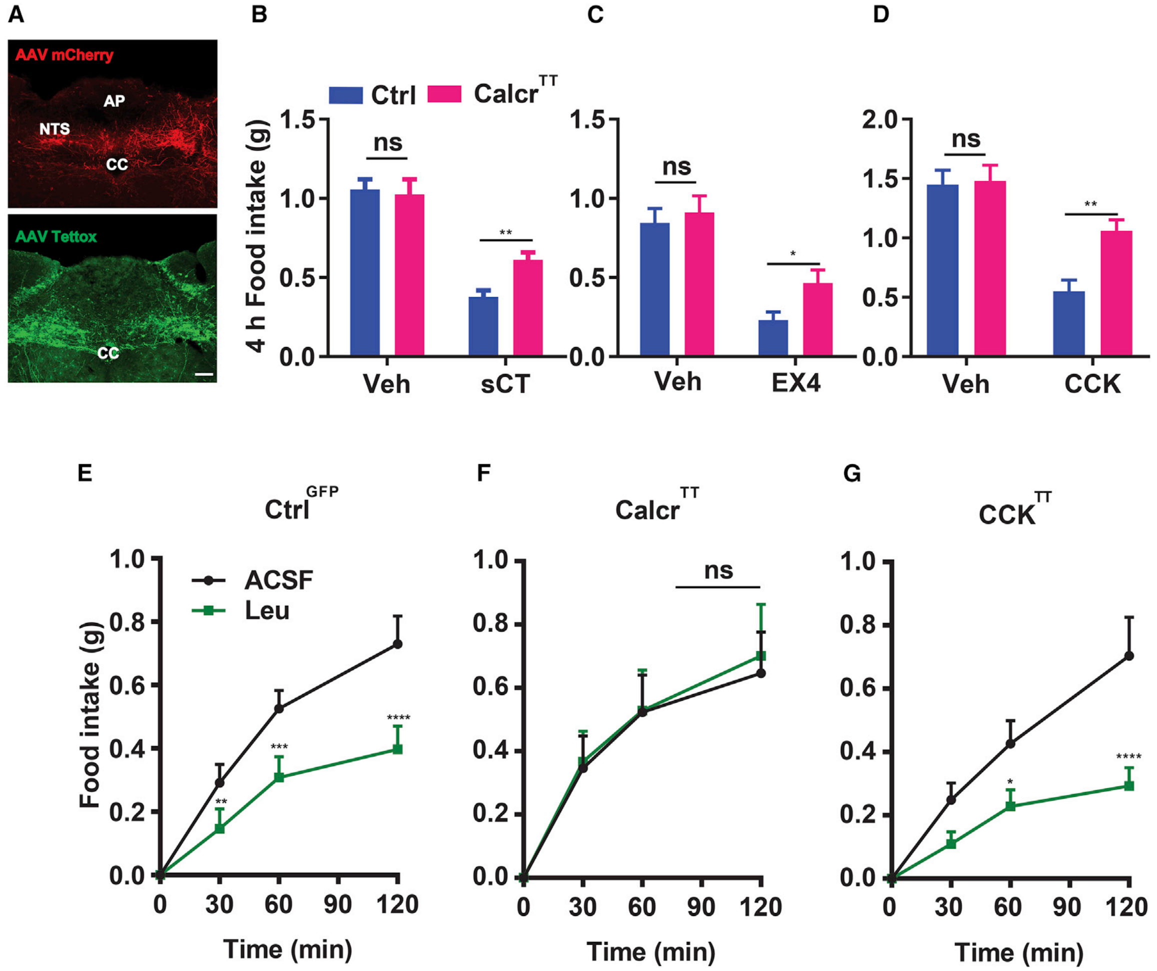


**Figure 6. Calcr$^{NTS}$ Neurons Contribute to the Suppression of Food Intake by Gut Peptide Receptors and Amino Acids**

(A) Representative images show viral transduction in control (Ctrl) (mCherry, red, top panel) and Calcr$^{NTS}$-TT (Calcr$^{TT}$) (GFP, green, bottom panel) mice. Scale bar, 150 μm; cc, central canal.

(B–D) Food intake was measured over the first 4 h of treatment with (B) sCT (150 μg/kg, n = 18 in control group, n = 16 in Calcr$^{TT}$ group), (C) EX4 (150 μg/kg, n = 14 in control group, n = 10-12 in Calcr$^{TT}$ group), and (D) CCK (100 μg/kg; n = 5 in both groups). Mean ± SEM, unpaired t test was performed between Ctrl and Calcr$^{TT}$ groups. *$p < 0.05$, **$p < 0.01$.

(E–G) Suppression of food intake at the onset of the dark cycle in response to the intra-NTS injection of vehicle (ACSF) or Leu for control (E, GFP, n = 6), Calcr$^{TT}$ (F, n = 7), and CCK$^{TT}$ (G, n = 8) mice. Shown is mean ± SEM. Two-way ANOVA, Sidak's multiple comparisons test was performed, *$p < 0.05$, **$p < 0.01$, ***$p < 0.001$, ****$p < 0.0001$. ns, not significant.

8 g of weight gain compared to controls. Body composition analysis revealed that adipose mass represented the majority of the excess weight gain by Calcr$^{NTS}$-TT mice (Figures 7E and 7F). Thus, silencing Calcr$^{NTS}$ cells increases long-term food intake, body weight, and adiposity (especially in mice exposed to a palatable diet), revealing that these NTS neurons play an important role in overall energy balance, not just in the short-term suppression of food intake in response to gut-derived signals.

## DISCUSSION

Our findings demonstrate that Calcr$^{NTS}$ neurons mediate the long-term suppression of food intake by gut peptide mimetics and are required for the normal physiologic regulation of feeding and energy balance. Moreover, the activation of Calcr$^{NTS}$ neurons is neither necessary nor sufficient for the acquisition of a CTA. In the context of the CTA-associated food intake suppression that results from the activation of Cck$^{NTS}$ cells (Roman et al., 2017), our findings with Calcr$^{NTS}$ neurons also demonstrate that the NTS systems that control the aversive and non-aversive suppression of feeding can be distinguished. Indeed, we show that while Calcr$^{NTS}$ and Cck$^{NTS}$ cells both inhibit MB-ARC neurons, Calcr$^{NTS}$ cells do not activate aversive CGRP$^{PBN}$ cells (as do Cck$^{NTS}$ cells), but rather suppress food intake via distinct PBN targets. Our findings also reveal that Calcr$^{NTS}$ cells not only participate in the short-term response to nutritional and gut peptide signals, but also play important roles in the long-term control of food intake and body weight, demonstrating a role for brainstem circuits in the control of overall energy balance, especially during exposure to HFD.

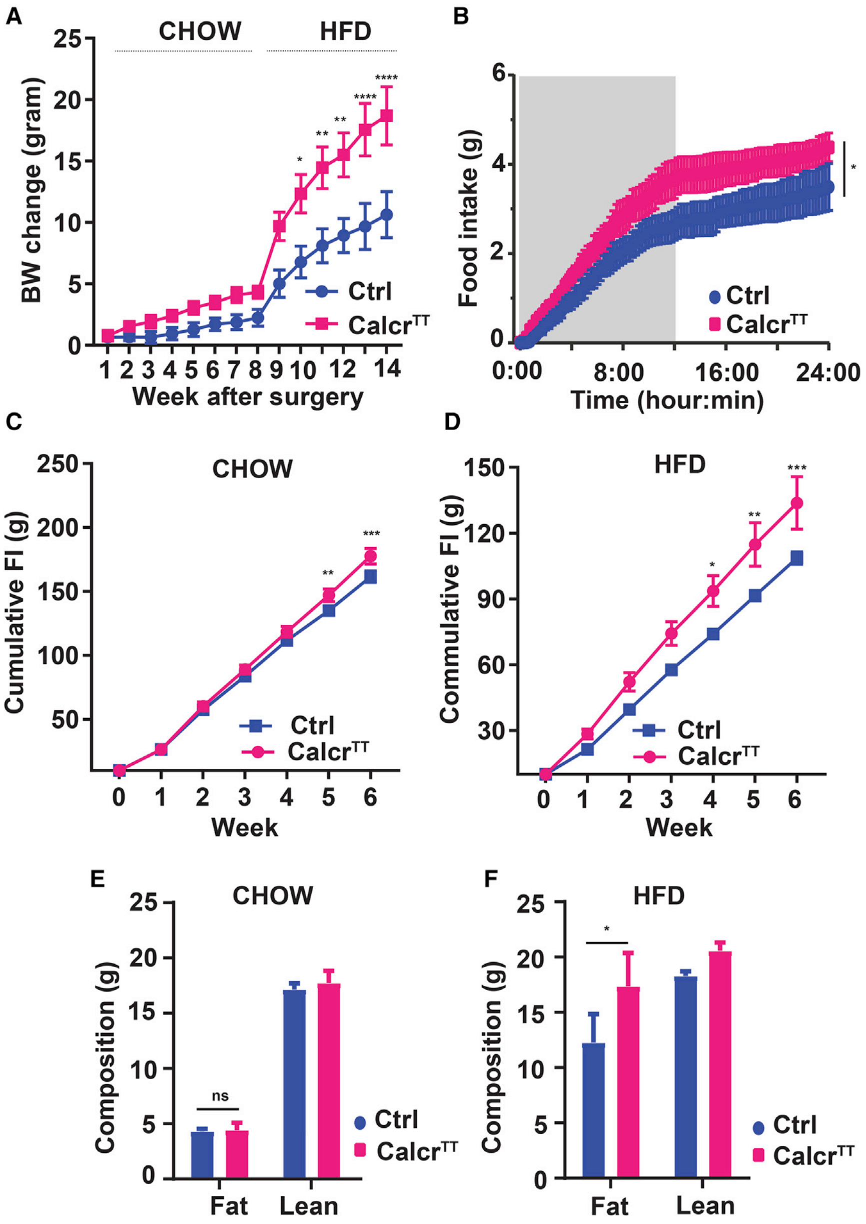


**Figure 7. Silencing Calcr$^{NTS}$ Neurons Increases Food Intake and Body Weight**

(A–F) Pairs of *Calcr$^{cre}$* mice were matched for initial body weight and injected with AAV$^{Flex-TetTox-GFP}$ (Calcr$^{TT}$) or AAV$^{Flex-mCherry}$ (Ctrl). Mice were subjected to chow diet for 7 weeks, followed by another 6 weeks on HFD. (A) Body weight (change from baseline) for the duration of the experiment is shown (n = 10 in each group). (B) Food intake was continuously monitored over 24 h in a TSE system during the seventh week after surgery (n = 5 per group). Cumulative food intake on chow (C, n = 10 per group) and HFD (D, n = 10 per group) are shown. Body composition was determined after 7 weeks on chow diet (E, n = 7 in control group, n = 5 in Calcr$^{TT}$ group) and 6 weeks on HFD (F, n = 5 per group). Mean ± SEM is shown. Two-way ANOVA, Sidak's multiple comparisons test was performed in (A), (C), and (D). Two-way repeated-measures ANOVA was performed for (B). Unpaired t test was performed for (E) and (F). *$p < 0.05$, **$p < 0.01$, ***$p < 0.001$, ****$p < 0.0001$. ns, not significant.

Because the activation of aversive symptoms can limit the utility of treatments designed to decrease food intake and body weight (Gianini et al., 2013), agents that activate neurons that suppress food intake without simultaneously stimulating aversive systems would represent ideal therapeutic agents. Hence Calcr$^{NTS}$ cells, which contribute to the long-term suppression of food intake without provoking a CTA, represent a potentially useful target for the control of food intake. While the activation of *Gcg*-expressing NTS neurons in mice also suppresses food intake without activating a CTA, the decrease in food intake mediated by these cells is relatively small and transient (Gaykema et al., 2017), and their downstream targets have not been identified.

In contrast to the important role for *Calcr* in Calcr$^{NTS}$ cells in CALCR agonist-mediated anorexia, *Calcr* in Sim1 or LepRb neurons is not required for this response to CALCR agonists. Furthermore, the undiminished PVH and ARC FOS responses to sCT in Calcr$^{Sim1}$KO and Calcr$^{LepRb}$KO mice, respectively, suggest that much sCT-induced hypothalamic FOS may be mediated indirectly, via *Calcr* neurons that project into the hypothalamus (potentially including Calcr$^{NTS}$ cells). Furthermore, Calcr$^{LepRb}$ neurons mainly represent orexigenic ARC NAG neurons (Pan et al., 2018), suggesting that the sCT-dependent direct activation of these cells might increase (rather than decrease) feeding. Indeed, we found that sCT slightly increases long-term food intake in Calcr$^{NTS}$KO mice, consistent with the notion that sCT activates a set of orexigenic neurons (such as Calcr$^{LepRb}$/NAG cells) but that this effect is normally masked by the anorexigenic action of Calcr$^{NTS}$ cells. Consistently, we find that Calcr$^{NTS}$ neuron activation overcomes feeding driven by ghrelin.

While Calcr$^{Sim1}$ and Calcr$^{LepRb}$ neurons are not required for the suppression of food intake by sCT, non-NTS *Calcr* neurons must participate in the acute anorectic response to sCT (during the first 1–2 h of treatment), even though Calcr$^{NTS}$ cells mediate the long-term suppression of food intake. Indeed, AP lesions can attenuate the sCT-mediated suppression of food intake over 1–2 h in rats (Braegger et al., 2014), suggesting that sCT action via AP *Calcr* neurons might mediate the short-term attenuation of food intake in response to CALCR agonists. Similarly, because Calcr$^{NTS}$ cells do not contribute to the aversive effects of sCT, mediate aversion, or contribute to the activation of CGRP$^{PBN}$ cells, non-NTS Calcr cells must mediate the CGRP$^{PBN}$-activating and aversive responses to sCT. Since the AP contributes to aversive signaling, including nausea (Wang

et al., 2013), it is possible that Calcr$^{AP}$ cells mediate the CTA-producing effects of sCT. Calcr$^{AP}$ cells could also mediate the Calcr$^{NTS}$-independent short-term suppression of food intake by sCT. Unfortunately, we and others have been unable to specifically target the AP by stereotaxic injection in mice, preventing us from directly testing this possibility.

To understand the potential circuit differences that underlie the aversive versus non-aversive suppression of food intake by Cck$^{NTS}$ and Calcr$^{NTS}$ cells, respectively, we first used MB-ARC FOS-IR as a surrogate to examine the control of NAG cells by Calcr$^{NTS}$ neurons, since gut signals (that are presumably mediated by the hindbrain) inhibit these cells (Beutler et al., 2017) and because we found Calcr$^{NTS}$ neuron activation blunts ghrelin-induced hyperphagia (which is mediated by NAG cells). Indeed, we found that the activation of Calcr$^{NTS}$ neurons inhibited MB-ARC FOS-IR. While the ultimate circuit underlying this effect will require further studies, it is possible that Calcr$^{NTS}$ cells project to and activate the GABAergic DMH cells that directly inhibit NAG cells (Garfield et al., 2016).

While the inhibition of MB-ARC neurons may contribute to the suppression of food intake by NTS neurons, Cck$^{NTS}$ and Calcr$^{NTS}$ cells similarly suppress MB-ARC neuron activity. Thus, the difference between the effects of Cck$^{NTS}$ and Calcr$^{NTS}$ neurons on aversion must lie in a distinct circuit. The DMH and/or PVH, both of which are innervated and activated by Cck$^{NTS}$ and Calcr$^{NTS}$ cells, could also contribute to the suppression of food intake by these NTS cells types. Indeed, activation of the Cck$^{NTS}$ → PVH circuit suppresses food intake (D'Agostino et al., 2016). Given that this projection from Cck$^{NTS}$ cells is non-aversive (D'Agostino et al., 2016), however, these differences in PVH projections between Cck$^{NTS}$ and Calcr$^{NTS}$ cells are unlikely to underlie the difference in aversive signaling by the NTS cell types.

Because of the important roles played by CGRP$^{PBN}$ cells in the aversive suppression of food intake (Campos et al., 2016), we examined the ability of Calcr$^{NTS}$ neurons to suppress feeding via the PBN. While PBN projections from Calcr$^{NTS}$ cells suppress feeding, these neurons innervate a distinct PBN region compared to Cck$^{NTS}$ cells and poorly activate CGRP$^{PBN}$ neurons compared to Cck$^{NTS}$ cells. Hence, non-aversive, non-CGRP PBN neurons contribute to the anorectic effects of Calcr$^{NTS}$ cells. Consistently, the PBN region activated by Calcr$^{NTS}$ cells appears similar to that which relays a positive-valence vagal response to nutrient ingestion (Han et al., 2018).

Calcr$^{NTS}$ neurons contribute to the suppression of food intake by a variety of gut peptide-mimetic stimuli (e.g., CCK and Ex4), as well as CALCR agonists. Calcr$^{NTS}$ cells, but not Cck$^{NTS}$ cells, also mediate the suppression of food intake by amino acids in the hindbrain (which do not produce a CTA). Although *Calcr* in Calcr$^{NTS}$ neurons is not required for normal energy balance, our data reveal the requirement for signaling by Calcr$^{NTS}$ neurons in the maintenance of long-term food intake control and energy homeostasis, especially in animals exposed to HFD. The importance of non-aversive Calcr$^{NTS}$ cells to limit the consumption of HFD is not only consistent with the failure of silencing CGRP$^{PBN}$ cells to alter long-term energy balance (Campos et al., 2016), but also suggests an important role for these cells in controlling the incentive value of palatable food. Thus, Calcr$^{NTS}$ cells and their downstream circuits represent potentially useful therapeutic targets for the treatment of obesity.

Overall, Calcr$^{NTS}$ neurons define a hindbrain system that suppresses food intake without activating aversive systems and participates in physiological energy balance (as well as in the anorectic response to pharmacologic activation). These results also demonstrate the separability of NTS circuits that mediate aversive anorexia from those that mediate the non-aversive suppression of food intake.

## Limitations of Study

Because hindbrain circuits differ in some respects between mice and rats (and presumably other mammals, including primates), it is possible that markers for NTS cell types that mediate aversive and non-aversive food intake suppression may differ by species. Furthermore, it will be important to identify the conditions under which, and mechanisms by which, the non-aversive NTS cells mediate their potential reinforcing effects.

## STAR★METHODS

Detailed methods are provided in the online version of this paper and include the following:

- KEY RESOURCES TABLE
- LEAD CONTACT AND MATERIALS AVAILABILITY
- EXPERIMENTAL MODEL AND SUBJECT DETAILS
  - Animals
- METHOD DETAILS
  - Viral Reagents and Stereotaxic Injections
  - Optogenetics
  - Chronic Cannula Implantation for NTS Injections
  - Phenotypic Studies
  - Perfusion and Immunohistochemistry
  - RNA Extraction, Reverse Transcription and RT-qPCR
  - Conditioned Taste Aversion (CTA)
  - CTA for Leu Injection
  - *In Situ* Hybridization (ISH)
  - Multiplexed FISH with RNAscope for Leu Related Experiments
- QUANTIFICATION AND STATISTICAL ANALYSIS
  - Statistics
- DATA AND CODE AVAILABILITY

## SUPPLEMENTAL INFORMATION

Supplemental Information can be found online at https://doi.org/10.1016/j.cmet.2019.12.012.


## ACKNOWLEDGMENTS

We thank B. Roth and K. Deisseroth for AAV plasmid constructs and Richard Palmiter for the gift of *Calca*$^{cre-GFP}$ mice. We thank members of the Myers and Olson labs for helpful discussions. Research support was provided by the Michigan Diabetes Research Center (NIH P30 DK020572, including the Molecular Genetics and Animal Studies Cores), the American Diabetes Association (1-16-PDF-021 to W.C. and 17-INI-15 to J.N.F.), the Marilyn H. Vincent Foundation (to M.G.M.), and MedImmune, LLC (to M.G.M.). This work was also supported in part by NIH Awards DK082480 (D.A.S.), T32 GM008322 (I.G.), and DK104999 (D.P.O.), and by research support from Boehringer Ingelheim (D.A.S. and R.J.S.), Ethicon Endo-Surgery Inc. (D.A.S. and R.J.S.), Sanofi (R.J.S.), and Novo Nordisk A/S (D.A.S. and R.J.S.). C.B. and her group would also like to thank Gregory Strachan at the Wellcome Trust-MRC Institute of Metabolic Science for his assistance with image analysis, and Julia Jones

and Heather Zecchini at the Cancer Research UK Cambridge Institute for their assistance with RNAscope staining and imaging. This work was supported by the Medical Research Council (MR/N003276/1), the Medical Research Council Metabolic Disease Unit, and the Wellcome Trust Strategic Award for the MRL Disease Model Core and Imaging facilities (MRC_MC_UU_12012/5 and 100574/Z/12/Z).

## AUTHOR CONTRIBUTIONS

W.C., I.G., W.P., A.H.T., J.A., E.N., D.G., B.K., K.R., S.S.E., A.M., S.W., C.B., and H.F.-S. researched and analyzed data. S.F. provided crucial reagents. W.C., I.G., W.P., J.L.T., C.J.R., R.J.S., D.A.S., D.P.O., C.B., and M.G.M. designed experiments and wrote and edited the manuscript. All authors reviewed and edited the manuscript. M.G.M. is the guarantor of the manuscript.

## DECLARATION OF INTERESTS

J.L.T. and C.J.R. are past or present employees of MedImmune, LLC and hold stock in AstraZeneca. The other authors declare that they have no conflicts of interest relevant to this manuscript.

Received: August 30, 2018
Revised: August 29, 2019
Accepted: December 20, 2019
Published: January 16, 2020

## REFERENCES

Adams, J.M., Pei, H., Sandoval, D.A., Seeley, R.J., Chang, R.B., Liberles, S.D., and Olson, D.P. (2018). Liraglutide Modulates Appetite and Body Weight Through Glucagon-Like Peptide 1 Receptor-Expressing Glutamatergic Neurons. Diabetes *67*, 1538–1548.

Balthasar, N., Dalgaard, L.T., Lee, C.E., Yu, J., Funahashi, H., Williams, T., Ferreira, M., Tang, V., McGovern, R.A., Kenny, C.D., et al. (2005). Divergence of melanocortin pathways in the control of food intake and energy expenditure. Cell *123*, 493–505.

Becskei, C., Riediger, T., Zünd, D., Wookey, P., and Lutz, T.A. (2004). Immunohistochemical mapping of calcitonin receptors in the adult rat brain. Brain Res. *1030*, 221–233.

Bergey, G.K., Bigalke, H., and Nelson, P.G. (1987). Differential effects of tetanus toxin on inhibitory and excitatory synaptic transmission in mammalian spinal cord neurons in culture: a presynaptic locus of action for tetanus toxin. J. Neurophysiol. *57*, 121–131.

Beutler, L.R., Chen, Y., Ahn, J.S., Lin, Y.C., Essner, R.A., and Knight, Z.A. (2017). Dynamics of Gut-Brain Communication Underlying Hunger. Neuron *96*, 461–475.e5.

Blouet, C., and Schwartz, G.J. (2012). Brainstem nutrient sensing in the nucleus of the solitary tract inhibits feeding. Cell Metab. *16*, 579–587.

Braegger, F.E., Asarian, L., Dahl, K., Lutz, T.A., and Boyle, C.N. (2014). The role of the area postrema in the anorectic effects of amylin and salmon calcitonin: behavioral and neuronal phenotyping. Eur. J. Neurosci. *40*, 3055–3066.

Campos, C.A., Bowen, A.J., Schwartz, M.W., and Palmiter, R.D. (2016). Parabrachial CGRP Neurons Control Meal Termination. Cell Metab. *23*, 811–820.

Carter, M.E., Soden, M.E., Zweifel, L.S., and Palmiter, R.D. (2013). Genetic identification of a neural circuit that suppresses appetite. Nature *503*, 111–114.

Carter, M.E., Han, S., and Palmiter, R.D. (2015). Parabrachial calcitonin gene-related peptide neurons mediate conditioned taste aversion. J. Neurosci. *35*, 4582–4586.

Cavanaugh, A.R., Schwartz, G.J., and Blouet, C. (2015). High-fat feeding impairs nutrient sensing and gut brain integration in the caudomedial nucleus of the solitary tract in mice. PLoS One *10*, e0118888.

D'Agostino, G., Lyons, D.J., Cristiano, C., Burke, L.K., Madara, J.C., Campbell, J.N., Garcia, A.P., Land, B.B., Lowell, B.B., Dileone, R.J., and Heisler, L.K. (2016). Appetite controlled by a cholecystokinin nucleus of the solitary tract to hypothalamus neurocircuit. eLife *5*, pii: e12225.

Fenno, L.E., Mattis, J., Ramakrishnan, C., Hyun, M., Lee, S.Y., He, M., Tucciarone, J., Selimbeyoglu, A., Berndt, A., Grosenick, L., et al. (2014). Targeting cells with single vectors using multiple-feature Boolean logic. Nat. Methods *11*, 763–772.

Flak, J.N., Arble, D., Pan, W., Patterson, C., Lanigan, T., Goforth, P.B., Sacksner, J., Joosten, M., Morgan, D.A., Allison, M.B., et al. (2017). A leptin-regulated circuit controls glucose mobilization during noxious stimuli. J. Clin. Invest. *127*, 3103–3113.

Franklin, K. and Paxinos, G. (2008). The Coronal Plates and Diagrams. The Mouse Brain in Stereotaxic Coordinates, Compact 3rd Edition (Academic Press).

Freed, W.J., Bing, L.A., and Wyatt, R.J. (1981). Calcitonin: aversive effects in rats? Science *211*, 734.

Fujioka, K. (2015). Current and emerging medications for overweight or obesity in people with comorbidities. Diabetes Obes. Metab. *17*, 1021–1032.

Garfield, A.S., Shah, B.P., Burgess, C.R., Li, M.M., Li, C., Steger, J.S., Madara, J.C., Campbell, J.N., Kroeger, D., Scammell, T.E., et al. (2016). Dynamic GABAergic afferent modulation of AgRP neurons. Nat. Neurosci. *19*, 1628–1635.

Gaykema, R.P., Newmyer, B.A., Ottolini, M., Raje, V., Warthen, D.M., Lambeth, P.S., Niccum, M., Yao, T., Huang, Y., Schulman, I.G., et al. (2017). Activation of murine pre-proglucagon-producing neurons reduces food intake and body weight. J. Clin. Invest. *127*, 1031–1045.

Gianini, L.M., White, M.A., and Masheb, R.M. (2013). Eating pathology, emotion regulation, and emotional overeating in obese adults with Binge Eating Disorder. Eat. Behav. *14*, 309–313.

Goodwin, L.O., Splinter, E., Davis, T.L., Urban, R., He, H., Braun, R.E., Chesler, E.J., Kumar, V., van Min, M., Ndukum, J., et al. (2019). Large-scale discovery of mouse transgenic integration sites reveals frequent structural variation and insertional mutagenesis. Genome Res. *29*, 494–505.

Grill, H.J., and Hayes, M.R. (2009). The nucleus tractus solitarius: a portal for visceral afferent signal processing, energy status assessment and integration of their combined effects on food intake. Int. J. Obes. *33* (*Suppl 1*), S11–S15.

Grill, H.J., and Hayes, M.R. (2012). Hindbrain neurons as an essential hub in the neuroanatomically distributed control of energy balance. Cell Metab. *16*, 296–309.

Halatchev, I.G., and Cone, R.D. (2005). Peripheral administration of PYY(3-36) produces conditioned taste aversion in mice. Cell Metab. *1*, 159–168.

Hayes, M.R., Skibicka, K.P., Leichner, T.M., Guarnieri, D.J., DiLeone, R.J., Bence, K.K., and Grill, H.J. (2010). Endogenous leptin signaling in the caudal nucleus tractus solitarius and area postrema is required for energy balance regulation. Cell Metab. *11*, 77–83.

Han, W., Tellez, L.A., Perkins, M.H., Perez, I.O., Qu, T., Ferreira, J., Ferreira, T.L., Quinn, D., Liu, Z.W., Gao, X.B., et al. (2018). A Neural Circuit for Gut-Induced Reward. Cell *175*, 665–678.

Hayes, M.R., Leichner, T.M., Zhao, S., Lee, G.S., Chowansky, A., Zimmer, D., De Jonghe, B.C., Kanoski, S.E., Grill, H.J., and Bence, K.K. (2011). Intracellular signals mediating the food intake-suppressive effects of hindbrain glucagon-like peptide-1 receptor activation. Cell Metab. *13*, 320–330.

Hayes, M.R., Leichner, T.M., Zhao, S., Lee, G.S., Chowansky, A., Zimmer, D., De Jonghe, B.C., Kanoski, S.E., Grill, H.J., and Bence, K.K. (2016a). Intracellular Signals Mediating the Food Intake-Suppressive Effects of Hindbrain Glucagon-like Peptide-1 Receptor Activation. Cell Metab. *23*, 745.

Hayes, M.R., Skibicka, K.P., Leichner, T.M., Guarnieri, D.J., DiLeone, R.J., Bence, K.K., and Grill, H.J. (2016b). Endogenous Leptin Signaling in the Caudal Nucleus Tractus Solitarius and Area Postrema Is Required for Energy Balance Regulation. Cell Metab. *23*, 744.

Kim, J.C., Cook, M.N., Carey, M.R., Shen, C., Regehr, W.G., and Dymecki, S.M. (2009). Linking genetically defined neurons to behavior through a broadly applicable silencing allele. Neuron *63*, 305–315.

Krashes, M.J., Koda, S., Ye, C., Rogan, S.C., Adams, A.C., Cusher, D.S., Maratos-Flier, E., Roth, B.L., and Lowell, B.B. (2011). Rapid, reversible activation of AgRP neurons drives feeding behavior in mice. J. Clin. Invest. *121*, 1424–1428.

CellPress


Ladenheim, E.E. (2015). Liraglutide and obesity: a review of the data so far. Drug Des. Devel. Ther. *9*, 1867–1875.

Lamprecht, M.R., Sabatini, D.M., and Carpenter, A.E. (2007). CellProfiler: free, versatile software for automated biological image analysis. Biotechniques *42*, 71–75.

Lutz, T.A., Geary, N., Szabady, M.M., Del Prete, E., and Scharrer, E. (1995). Amylin decreases meal size in rats. Physiol. Behav. *58*, 1197–1202.

Mack, C.M., Soares, C.J., Wilson, J.K., Athanacio, J.R., Turek, V.F., Trevaskis, J.L., Roth, J.D., Smith, P.A., Gedulin, B., Jodka, C.M., et al. (2010). Davalintide (AC2307), a novel amylin-mimetic peptide: enhanced pharmacological properties over native amylin to reduce food intake and body weight. Int. J. Obes. *34*, 385–395.

Mietlicki-Baase, E.G., Ortinski, P.I., Rupprecht, L.E., Olivos, D.R., Alhadeff, A.L., Pierce, R.C., and Hayes, M.R. (2013). The food intake-suppressive effects of glucagon-like peptide-1 receptor signaling in the ventral tegmental area are mediated by AMPA/kainate receptors. Am. J. Physiol. Endocrinol. Metab. *305*, E1367–E1374.

Olofsson, L.E., Pierce, A.A., and Xu, A.W. (2009). Functional requirement of AgRP and NPY neurons in ovarian cycle-dependent regulation of food intake. Proc. Natl. Acad. Sci. USA *106*, 15932–15937.

Opland, D., Sutton, A., Woodworth, H., Brown, J., Bugescu, R., Garcia, A., Christensen, L., Rhodes, C., Myers, M., Jr., and Leinninger, G. (2013). Loss of neurotensin receptor-1 disrupts the control of the mesolimbic dopamine system by leptin and promotes hedonic feeding and obesity. Mol. Metab. *2*, 423–434.

Pan, W., Adams, J.M., Allison, M.B., Patterson, C., Flak, J.N., Jones, J., Strohbehn, G., Trevaskis, J., Rhodes, C.J., Olson, D.P., and Myers, M.G., Jr. (2018). Essential Role for Hypothalamic Calcitonin Receptor–Expressing Neurons in the Control of Food Intake by Leptin. Endocrinology *159*, 1860–1872.

Patterson, C.M., Leshan, R.L., Jones, J.C., and Myers, M.G., Jr. (2011). Molecular mapping of mouse brain regions innervated by leptin receptor-expressing cells. Brain Res. *1378*, 18–28.

Pinto, S., Roseberry, A.G., Liu, H., Diano, S., Shanabrough, M., Cai, X., Friedman, J.M., and Horvath, T.L. (2004). Rapid rewiring of arcuate nucleus feeding circuits by leptin. Science *304*, 110–115.

Roman, C.W., Derkach, V.A., and Palmiter, R.D. (2016). Genetically and functionally defined NTS to PBN brain circuits mediating anorexia. Nat. Commun. *7*, 11905.

Roman, C.W., Sloat, S.R., and Palmiter, R.D. (2017). A tale of two circuits: $CCK^{NTS}$ neuron stimulation controls appetite and induces opposing motivational states by projections to distinct brain regions. Neuroscience *358*, 316–324.

Schier, L.A., Davidson, T.L., and Powley, T.L. (2012). Rapid stimulus-bound suppression of intake in response to an intraduodenal nonnutritive sweetener after training with nutritive sugars predicting malaise. Am. J. Physiol. Regul. Integr. Comp. Physiol. *302*, R1351–R1363.

Sclafani, A. (2013). Gut-brain nutrient signaling. Appetition vs. satiation. Appetite *71*, 454–458.

Sclafani, A., and Ackroff, K. (2012). Role of gut nutrient sensing in stimulating appetite and conditioning food preferences. Am. J. Physiol. Regul. Integr. Comp. Physiol. *302*, R1119–R1133.

Secher, A., Jelsing, J., Baquero, A.F., Hecksher-Sørensen, J., Cowley, M.A., Dalbøge, L.S., Hansen, G., Grove, K.L., Pyke, C., Raun, K., et al. (2014). The arcuate nucleus mediates GLP-1 receptor agonist liraglutide-dependent weight loss. J. Clin. Invest. *124*, 4473–4488.

Smith, K.S., Bucci, D.J., Luikart, B.W., and Mahler, S.V. (2016). DREADDS: Use and application in behavioral neuroscience. Behav. Neurosci. *130*, 137–155.

Vasanth Rao, V.R.B., Candasamy, M., and Bhattamisra, S.K. (2019). Obesity an overview: Genetic conditions and recent developments in therapeutic interventions. Diabetes Metab. Syndr. *13*, 2112–2120.

Verbaeys, I., León-Tamariz, F., Pottel, H., Decuypere, E., Swennen, Q., and Cokelaere, M. (2008). PEGylated cholecystokinin is more potent in inducing anorexia than conditioned taste aversion in rats. Br. J. Pharmacol. *155*, 417–423.

Wang, F., Flanagan, J., Su, N., Wang, L.-C., Bui, S., Nielson, A., Wu, X., Vo, H.-T., Ma, X.-J., and Luo, Y. (2012). RNAscope: a novel in situ RNA analysis platform for formalin-fixed, paraffin-embedded tissues. J. Mol. Diagn. *14*, 22–29.

Wang, T.J.C., Fontenla, S., McCann, P., Young, R.J., McNamara, S., Rao, S., Mechalakos, J.G., and Lee, N.Y. (2013). Correlation of Planned Dose to Area Postrema and Dorsal Vagal Complex with Clinical Symptoms of Nausea and Vomiting in Oropharyngeal Cancer (OPC) patients treated with radiation alone using IMRT. J. Radiat. Oncol. *2*, 407–412.

Wickersham, I.R., Lyon, D.C., Barnard, R.J., Mori, T., Finke, S., Conzelmann, K.K., Young, J.A., and Callaway, E.M. (2007). Monosynaptic restriction of transsynaptic tracing from single, genetically targeted neurons. Neuron *53*, 639–647.

Yamaguchi, M., Watanabe, Y., Ohtani, T., Uezumi, A., Mikami, N., Nakamura, M., Sato, T., Ikawa, M., Hoshino, M., Tsuchida, K., et al. (2015). Calcitonin Receptor Signaling Inhibits Muscle Stem Cells from Escaping the Quiescent State and the Niche. Cell Rep. *13*, 302–314.

Zhu, H., and Roth, B.L. (2014). DREADD: a chemogenetic GPCR signaling platform. Int. J. Neuropsychopharmacol. *18*, pii: pyu007.

# STAR★METHODS

## KEY RESOURCES TABLE

| REAGENT or RESOURCE | SOURCE | IDENTIFIER |
|---|---|---|
| Antibodies | | |
| FOS (DAB staining) | Santa Cruz | SC-52; RRID:AB_2106783 |
| pSTAT3 | Cell Signaling | 9145; RRID:AB_2491009 |
| FOS (Immunofluorescence) | Cell Signaling | 2250; RRID:AB_2247211 |
| CGRP | AbCam | Ab81887; RRID:AB_1658411 |
| GFP | Aves Laboratories | GFP1020; RRID:AB_10000240 |
| dsRed | Takara | 632496; RRID:AB_10013483 |
| TH | Novus Biologicals | NB300-109; RRID:AB_350437 |
| Bacterial and Virus Strains | | |
| Ad-iN-Syn-mCherry | Opland et al., 2013 | N/A |
| AAVFlex-hM3Dq | Krashes et al., 2011 | N/A |
| AAVFlexTetTox-GFP | Kim et al., 2009 | N/A |
| AAVFlex-ChR2 | Fenno et al., 2014 | N/A |
| AAVFlex-GFP | Fenno et al., 2014 | N/A |
| AAVFlexTVA+G | Flak et al., 2017 | N/A |
| Defective pseudotyped rabies virus- tdTomato | Wickersham et al., 2007; University of Michigan Viral Vector Core | N/A |
| AAVFlex-hM4Di | Smith et al., 2016; AddGene | AddGene Cat#44362-AAV8 |
| Chemicals, Peptides, and Recombinant Proteins | | |
| CNO | Tocris | 4936 |
| sCT | Bachem | 4033011 |
| Davalintide | AstraZenica, Inc | N/A |
| PYY3-36 | Bachem | 4018889 |
| LPS | Sigma | L2630 |
| Exendin 4 | Tocris | 6355 |
| CCK octapeptide (sulfated) ammonium | Bachem | H-2080 |
| Leptin | AstraZenica, Inc | N/A |
| LiCl | Sigma | 203637 |
| Experimental Models: Organisms/Strains | | |
| Calcr-cre | Pan et al., 2018 | N/A |
| Calcr-flox | Yamaguchi et al., 2015 | N/A |
| Cck-cre | Jackson laboratories | 012706 |
| Th-cre | Jackson laboratories | 008601 |
| NPY-Gfp | Pinto et al., 2004 | N/A |
| Al14 (cre-inducible tdTomato) | Jackson laboratories | 007914 |
| Lepr-cre | Patterson et al., 2011; Jackson laboratories | 032457 |
| Sim1cre | Balthasar et al., 2005; Jackson laboratories | 006451 |
| Calca-cre-GFP | Carter et al., 2013 | N/A |
| Oligonucleotides | | |
| Calcr primers for qPCR: GCTGCTGGATGCTCAGTACA; AGTGTCGTCCCAGCACATC | IDT | N/A |
| Hprt primers for qPCR: GATTAGCGATGATGAACCAGGTT; CCTCCCATCTCCTTCATGACA | IDT | N/A |

(*Continued on next page*)

| Continued | | |
|---|---|---|
| REAGENT or RESOURCE | SOURCE | IDENTIFIER |
| Software and Algorithms | | |
| Prism v 7 | Graphpad | N/A https://www.graphpad.com/scientific-software/prism/ |
| Harmony | Perkin Elmer | www.perkinelmer.com/product/harmony |
| Photoshop | Adobe | www.adobe.com/products/photoshop.html |
| ImageJ | NIH | https://imagej.nih.gov/ij/download.html |

## LEAD CONTACT AND MATERIALS AVAILABILITY

Further information and requests for resources and reagents should be directed to and will be fulfilled by the Lead Contact, MGM (mgmyers@umich.edu). This study did not generate new unique reagents.

## EXPERIMENTAL MODEL AND SUBJECT DETAILS

### Animals

Mice were bred in our colony in the Unit for Laboratory Animal Medicine at the University of Michigan (except as noted below for mice tested at the University of Cambridge); these mice and the procedures performed were approved by the University of Michigan Committee on the Use and Care of Animals and in accordance with Association for the Assessment and Approval of Laboratory Animal Care and National Institutes of Health guidelines. Mice at both sites were provided with food (Purina Lab Diet 5001, unless otherwise specificed) and water *ad libitum* (except as noted below) in temperature-controlled (25°C) rooms on a 12 h light-dark cycle with daily health status checks.

We purchased male and female C57BL/6 mice for experiments and breeding from Jackson Laboratories. *Calcr*$^{cre}$,*Calcr*$^{flox}$, and *Cck*$^{cre}$ mice have been described (D'Agostino et al., 2016; Pan et al., 2018; Roman et al., 2016; Yamaguchi et al., 2015) and were propagated by intercrossing homozygous mice of the same genotype. *Th*$^{cre}$ mice (Goodwin et al., 2019) were purchased from Jackson Laboratory (Stock No: 008601). NPY–green fluorescent protein (GFP) transgenic mice are as described (Pinto et al., 2004). Cre-inducible tdTomato reporter mice (AI14) were purchased from Jackson Laboratory (Stock No: 007914), and were crossed with *Calcr*$^{cre}$ mice to generate Calcr$^{cre}$; tdTomato reporter mice.

*Calca*$^{cre\text{-}GFP}$ mice, the generous gift of Richard Palmiter (University of Washington) have been described (Carter et al., 2013) and were crossed with *Calcr*$^{cre}$ or *Cck*$^{cre}$ to generate *Calca*$^{cre\text{-}GFP/+}$; *Calcr*$^{cre/+}$ or *Calca*$^{cre\text{-}GFP/+}$; *Cck*$^{cre/+}$ mice to permit tracing and FOS studies with the visualization of *Calca*-containing CGRP$^{PBN}$ neurons.

*Calcr*$^{flox}$ mice were crossed twice onto the *Lepr*$^{cre}$ (Patterson et al., 2011) background to generate *Lepr*$^{cre/cre}$;*Calcr*$^{flox/+}$ mice, which were intercrossed to generate *Lepr*$^{cre/cre}$;*Calcr*$^{flox/flox}$ (Calcr$^{LepRb}$KO) and *Lepr*$^{cre/cre}$ control mice. *Calcr*$^{flox}$ mice were also crossed twice onto *Sim1*$^{cre}$ (Balthasar et al., 2005) to generate *Sim1*$^{cre/+}$;*Calcr*$^{flox/flox}$ mice, which were bred to *Calcr*$^{flox/flox}$ mice to generate *Sim1*$^{cre/+}$;*Calcr*$^{flox/flox}$ (Calcr$^{Sim1}$KO) and *Calcr*$^{flox/flox}$ controls for study. For all studies, animals were processed in the order of their ear tag number, which was randomly assigned at the time of tailing (before genotyping). All mouse models (with the exception of purchased C57BL/6 mice) were on the segregating C57BL/6;SJL;129 genetic background; sibling controls were used throughout. Male and female mice were used for all experiments where a single sex was not indicated; no sex differences in phenotype were detected.

For experiments performed at the University of Cambridge, all mice were single-housed in individually ventilated cages with standard bedding and enrichment in a temperature and humidity-controlled room on a 12-h light/dark cycle with *ad libitum* access to water and standard laboratory chow diet unless otherwise stated. 8-weeks old C57/Bl6J males were obtained from Charles River UK. All studies were approved by the local Ethics Committee and conducted according to the UK Home Office Animals (Scientific Procedures) Act 1986.

## METHOD DETAILS

### Viral Reagents and Stereotaxic Injections

Ad-iN-Syn-mCherry was generated and produced as described previously (Opland et al., 2013). AAV$^{Flex\text{-}hM3Dq}$ (Krashes et al., 2011), AAV$^{Flex\text{-}TetTox\text{-}GFP}$(Kim et al., 2009), AAV$^{Flex\text{-}ChR2}$, AAV$^{GFP}$ and AAV$^{Cre\text{-}mCherry}$ (Fenno et al., 2014) were as previously described, and were prepared by the University of North Carolina Vector Core (Chapel Hill, NC). AAV$^{Flex\text{-}TVA+G}$ and defective psuedotyped rabies-mCherry (Flak et al., 2017; Wickersham et al., 2007) were generated by the University of Michigan viral vector core. AAV$^{Flex\text{-}hM4Di}$ (Smith et al., 2016) was ordered through Addgene (Catalog #: 44362-AAV8).

For injection, following the induction of isoflurane anesthesia and placement in a stereotaxic frame, the skulls of adult mice were exposed. For NTS injection, the obex was set as reference point for injection. After the reference was determined, a guide cannula with a pipette injector was lowered into the approximate NTS coordinates, which was A/P, −0.2; M/L, ± 0.2; D/V, −0.2 from the obex

and 100 nL of virus was injected with using a picospritzer at a rate of 5-30 nL/min with pulses. Five minutes following injection, to allow for adequate dispersal and absorption of the virus, the injector was removed from the animal; the incision site was closed and glued. The mice received prophylactic analgesics before and after surgery (carprofen, 5 mg/kg, SQ). Mice were 8-12 weeks of age at the time of injection and were studied beginning 3-4 weeks post-surgery.

The mice injected with Ad-iN-Syn-mCherry were allowed one week to recover before being euthanized; the mice injected with $AAV^{Flex-hM3Dq}$, $AAV^{Flex-hM4Di}$, $AAV^{Flex-ChR2}$, $AAV^{Cre-mCherry}$, $AAV^{Flex-TetTox-GFP}$, or control viruses were allowed at least 1 week to recover from surgery before experimentation.

## Optogenetics

As described above, $AAV^{Flex-ChR2}$ virus was delivered in NTS for $Calcr^{cre}$ and $Cck^{cre}$ mice, two fiber-optic cannulae (Doric Lenses and Thorlabs) were implanted above the lateral PBN (A/P: 5.2 mm, M/L: ± 1.6 mm, D/V: 2.9 mm) and affixed to the skull using Metabond (Fisher). After 3 weeks recovery from surgery, the mice were then subjected to optical stimulation using 473 nm wavelength by 1 s of 20Hz photo stimulation and 3 s resting with multiple repetitions for 1 to 2 h period of time, during which time food intake was measured.

## Chronic Cannula Implantation for NTS Injections

Surgical procedures were performed under isoflurane anesthesia on animals that were 12-16 weeks of age. Animals were stereotaxically implanted with bilateral steel guide cannulae (Plastics One) positioned 2.0 mm above the caudomedial nucleus of the solitary tract (cannula holding bar in a 10° rostro-caudal angle, coordinates relative to occipital suture: A/P +0.5 mm, D/V-3 mm. +/− 0.4 lateral to midline). Beveled stainless steel injectors (33 gauge mounted onto a 26-gauge sleeve) extending 2.0 mm from the tip of the guide cannulas were used for injections. Animals were allowed a 1-week recovery during which they were handled daily and acclimatised to the brain injection procedure. Correct bilateral NTS cannula placement was confirmed histologically postmortem. Mice were randomly assigned to treatment groups and received a bilateral injection of 50nl/side vehicle (aCSF, Harvard Apparatus) or 2.1mM leucine via the pre-implanted cannula targeting the NTS in a cross-over manner by an experimenter blinded to genotype and treatment. Mice were fasted for 6 h before the injections and injections occurred 1 h before dark onset as previously described (Cavanaugh et al., 2015).

## Phenotypic Studies

Animals were singly housed from the time of weaning ($Calcr^{LepRb}$KO and $Calcr^{Sim1}$KO) or beginning 7 days after surgery ($Calcr^{NTS}$KO). Food intake and body weight were monitored weekly. $Calcr^{NTS}$TetTox mice and their controls were monitored from the time of surgery, and were also studied for meal patterns in metabolic chambers (TSE Systems Inc) during the final week of chow feeding.

For stimulation studies, KO mice, DREADD-expressing mice and their controls that were either at least two-months old or two months post-surgery were treated with saline or drugs (CNO, 4936, Tocris; sCT, 4033011, Bachem; Davalintide and Leptin, AstraZenica/MedImmune from original lots produced at Amylin Pharmaceuticals; PYY3-36, 4018889, Bachem; LPS, L2630, Sigma; Exendin 4, 6355, Tocris; Cholecystokinin Octapeptide (sulfated) ammonium, H-2080, Bachem) at the onset of dark cycle, food intake was monitored over four h. For chronic food intake and body weight changes, mice were given saline for two to three days prior to injecting saline or drugs twice per day (approximately 5:30 PM and 8:00 AM) for 3 or 4 days, followed by saline injections for another two or three days to assess recovery from the treatment.

## Perfusion and Immunohistochemistry

Mice (8 weeks of age or older) were anesthetized with a lethal dose of pentobarbital and transcardially perfused with phosphate-buffered saline (PBS) followed by 10% buffered formalin. Brains were removed, placed in 10% buffered formalin overnight, and dehydrated in 30% sucrose for 1 week. With use of a freezing microtome (Leica, Buffalo Grove, IL), brains were cut into 30-μm sections. Sections were treated sequentially with 1% hydrogen peroxide/0.5% sodium hydroxide, 0.3% glycine, 0.03% sodium dodecyl sulfate, and blocking solution (PBS with 0.1% triton, 3% normal donkey serum). The sections were incubated overnight at room temperature in rabbit anti-FOS (Santa Cruz, sc-52; 1: 1,000), and exposed the next day with either biotinylated (1:200 followed by avidin-biotin complex (ABC) amplification and 3,3-diaminobenzidine (DAB) reaction) or fluorescent secondary antibody (Molecular Probes, 1:200) to visualize proteins. Immunofluorescent staining was performed using primary antibodies (pSTAT3, #9145, Cell Signaling Technology, 1:250; FOS, #2250, Cell Signaling Technology, 1:1000; CGRP, ab81887, 1:1000, Abcam; GFP, GFP1020, Aves Laboratories, 1:1000; dsRed, 632496, Takara, 1:1000; Tyrosine Hydroxylase, NB300-109, Novus Biologicals, 1:1000), antibodies were reacted with species-specific Alexa Fluor-488, −568 or −647 conjugated secondary antibodies (Invitrogen, Thermo Fisher, 1:200). Images were collected on an Olympus (Center Valley, PA) BX53F microscope. images were pseudocolored using Photoshop software (Adobe) or ImageJ (NIH).

## RNA Extraction, Reverse Transcription and RT-qPCR

$Calcr^{Sim1}$KO and control mice (8 weeks of age or older) were euthanized with inhalation anesthetic isoflurane (McKesson, Piramal Critical Care #6679401725) using a drop jar. Microdissected PVH tissue for RNA preparation was stored in a −80°C freezer. RNA extraction was conducted using an RNeasy Mini kit (QIAGEN) and quantified using a NanoDrop 1000 (Thermo Scientific). RNA was treated with DNase (TURBO DNA-free Kit, Thermo Fisher Scientific, Cat #AM1907) and again quantified using a NanoDrop

1000. Approximately 500 ng of total RNA per sample underwent reverse transcription (iScript cDNA Synthesis Kit, Bio-Rad, Cat #1708891) using a thermal cycler (Mastercycler pro S, Eppendorf). Quantitative PCR (qPCR) was conducted using a StepOnePlus Real Time PCR System (Applied Biosystems) with SYBR Green PCR Master Mix (Applied Biosystems, Cat #4309155) and CalcR primers (Forward: 5′-GCTGCTGGATGCTCAGTACA-3′, Reverse: 5′-AGTGTCGTCCCAGCACATC-3′). The hypoxanthine guanine phosphoribosyl transferase (*Hprt*) gene was used as a reference gene (amplified using primers; Forward: 5′-GATTAGCGATGATG AACCAGGTT-3′, Reverse: 5′-CCTCCCATCTCCTTCATGACA-3′). A standard curve ladder was generated by pooling the cDNA from each sample and making 5 serial dilutions. From this, a standard curve line of best fit was produced, which allowed us to determine the relative RNA concentration of each sample. Samples were run in duplicate. These values were subjected to a Grubbs' test to discard any of the outliers before conducting statistical analysis.

## Conditioned Taste Aversion (CTA)

Two-flavoured drink CTA was performed as described (Halatchev and Cone, 2005). Briefly, mice (12 weeks of age or older) were individually housed in standard cages with low wire tops and free access to food and the lixits were removed. Mice were habituated to two water-containing bottles for 3-5 days until they learned to concentrate intake at 1000 h–1100 h. On conditioning days, mice were given 1 h access to either 0.05% cherry Kool-Aid paired with saline or grape Kool-Aid paired with the stimulus (e.g., LiCl, peptides, or CNO (0.8 mg/kg, 4936, Tocris Bioscience)). Mice were conditioned with these novel drinks for two rounds. Post conditioning day, mice were provided free access to the two flavoured drinks and two h intake were measured for each tastant.

Saccharin CTA: Mice that were 8 weeks of age or older were individually housed in standard cages with low wire tops and free access to food and the lixits were removed. Mice were habituated to two water-containing bottles for 3-5 days until they learned to concentrate their daily water consumption into these two water bottles. On the conditioning day, the mice received only two saccharin (0.15%, 240931, Sigma) bottles. Following the 30 min exposure to saccharin, mice were injected intraperitoneally with the desired stimulus (vehicle control (0.9% NaCl), lithium chloride (0.3 M, 203637, Sigma)) in a volume equivalent to 1% of each animal's body weight (10 μL/g), or CNO, or sCT (150 μg/kg, 4033011, Bachem). Access to the two saccharin bottles continued for an additional 2 h, followed by the return of normal water bottles. Two days later, each mouse received access to two water bottles (one containing 0.15% saccharine, the other containing water), and the amount of fluid ingested from each water bottle was be measured.

High fat diet (HFD) CTA: following an overnight fast, mice that were 8 weeks of age or older were conditioned with HFD and paired with desired stimuli for 30 min following with an extra one h access for HFD (D012492, Research Diets). On the post-conditioning day, fasted mice received access to both HFD and chow and the consumption of each was measured.

## CTA for Leu Injection

C57/Bl6J mice (n = 6 per group) implanted with a cannula targeting the NTS were singly housed with free access the two water bottles for 7 days. On days 1-3, mice of 12 weeks of age or older were water-deprived overnight and allowed access to water for 60 min in the morning and free access in the afternoon. On day 4, after overnight water deprivation, mice received an injection of aCSF or Leucine into the NTS (as above) followed by exposure to water containing 5% sucrose with either 0.05% grape or cherry flavour for 60 min. This was repeated 3 days later in a cross-over manner. On days 5-7 and 8-10, mice were exposed to the same regimen as on days 1-3. On day 8, after overnight water deprivation, mice were given access to a choice of two water bottles containing 5% sucrose with 0.05% grape or cherry flavour for 60 min and intake was recorded. ip LiCl was used as a positive control to induce conditioned taste aversion with the same paradigm.

## *In Situ* Hybridization (ISH)

Three adult female *Cck*$^{Cre/+}$*;TdTomato/+* mice of at least 8 weeks of age were anesthetized with isoflurane and then euthanized by decapitation. Brains were dissected, flash frozen in isopentane chilled on dry ice, and stored at −80°C. Sections were sliced at 16 μm thickness using a cryostat (Leica) and every fourth section was thaw-mounted onto SuperFrost Plus slides, allowed to dry for one h at −20°C, and then further stored at −80°C. Slides were then processed for RNAScope ISH per the manufacturer's protocol (Advanced Cell Diagnostics). The multiplex fluorescent assay (320850) was used to visualize *Calcr* (477791) and Cre (312281-C3) probes using Amp 4 Alt-A. At each of 8 coronal planes for each mouse, 4 images comprising the entire NTS/AP complex were obtained with a QImaging Retiga 6000 monochrome camera attached to an Olympus BX53 fluorescent microscope under 20X objective. The four images were then stitched together using Photoshop (Adobe). CellProfiler (Lamprecht et al., 2007) was used to process all images identically to remove nonspecific background, outline specific cells using the DAPI nuclear signal and analyze presence or absence of signal for both probes. For each mouse, the total number of cells identified as positive for either or both probes were added from all 8 coronal planes (for NTS) or for the subset of the 6 planes where AP was present. Subsequently, the sums from the three mice were averaged for each region.

## Multiplexed FISH with RNAscope for Leu Related Experiments

Brains were postfixed in 4% PFA solution overnight then cryoprotected in 30% sucrose solution in PBS for up to 24 h. Tissue was covered with optimal cutting temperature (OCT) media then sliced at 16 μm thickness using a Leica CM1950 cryostat directly onto Superfrost Plus slides (ThermoScientific) in an RNase free environment. Slides were then stored at −80°C. Sections were sliced in the coronal plane from Bregma −1.58 to −2.30 mm (Franklin, K. and Paxinos, G., 2008).

Fluorescence multiplex *in situ* RNA hybridization (FISH) was performed as previously described using RNAscope technology (Wang et al., 2012). After epitope retrieval and dehydration, sections on slides were processed for multiplexed FISH using the RNA-Scope LS Multiplex Assay (Advanced Cell Diagnostics). Samples were first permeabilized with heat in Bond Epitope Retrieval solution 2 (pH 9.0, Leica - AR9640) at 95°C for 2 min, incubated in protease reagent (Advanced Cell Diagnostics) at 42°C for 10 min, and finally treated with hydrogen peroxide for 10 min to inactivate endogenous peroxidases and the protease reagent. Samples were then incubated in z-probe mixtures (FOS 1:1, Calcr 1:50) for 2 h at 42°C and washed 3 times. DNA amplification trees were built through incubations in AMP1 (preamplifier), AMP2 (background reducer), then AMP3 (amplifier) reagents (Leica) for 15-30 min each at 42°C. Between incubations, slides were washed with LS Rinse buffer (Leica). After, samples were incubated in channel-specific horseradish peroxidase (HRP) reagents for 15 min at 42°C, TSA fluorophores for 30 min and HRP blocking reagent for 15 min at 42°C. The following TSA labels were used to visualize z-probes: Cy3 (1:500), FITC (1:500), and Cy5 (1:500) fluorophores (Perkin Elmer).

Brain sections were imaged using a spinning disk Operetta CLS (Perkin Elmer) in confocal mode using a sCMOS camera and a 40x automated-water dispensing objective. Sections were imaged with z stacks at intervals of 1 μm. ROIs included the NTS, AP and DMX. Gain and laser power settings remained the same between experimental and control conditions within each experiment. Harmony software (Perkin Elmer) was used to automatically quantify number of labeled RNA molecules (spots) per cell, and number of labeled cells.

## QUANTIFICATION AND STATISTICAL ANALYSIS

### Statistics

Data are reported as mean ± standard error of the mean. Statistical analyses of physiologic data were performed with Prism software (version 7), including testing to ensure the data fit a normal distribution. Two way ANOVA, paired or unpaired t tests were used as indicated in the text and figure legends. $p < 0.05$ was considered statistically significant.

## DATA AND CODE AVAILABILITY

This study did not generate any unique datasets or code.

Cell Metabolism, Volume *31*

## Supplemental Information

# Calcitonin Receptor Neurons in the Mouse

# Nucleus Tractus Solitarius Control Energy Balance

# via the Non-aversive Suppression of Feeding

**Wenwen Cheng, Ian Gonzalez, Warren Pan, Anthony H. Tsang, Jessica Adams, Ermelinda Ndoka, Desiree Gordian, Basma Khoury, Karen Roelofs, Simon S. Evers, Andrew MacKinnon, Shuangcheng Wu, Henriette Frikke-Schmidt, Jonathan N. Flak, James L. Trevaskis, Christopher J. Rhodes, So-ichiro Fukada, Randy J. Seeley, Darleen A. Sandoval, David P. Olson, Clemence Blouet, and Martin G. Myers Jr.**

Supplemental Figure 1

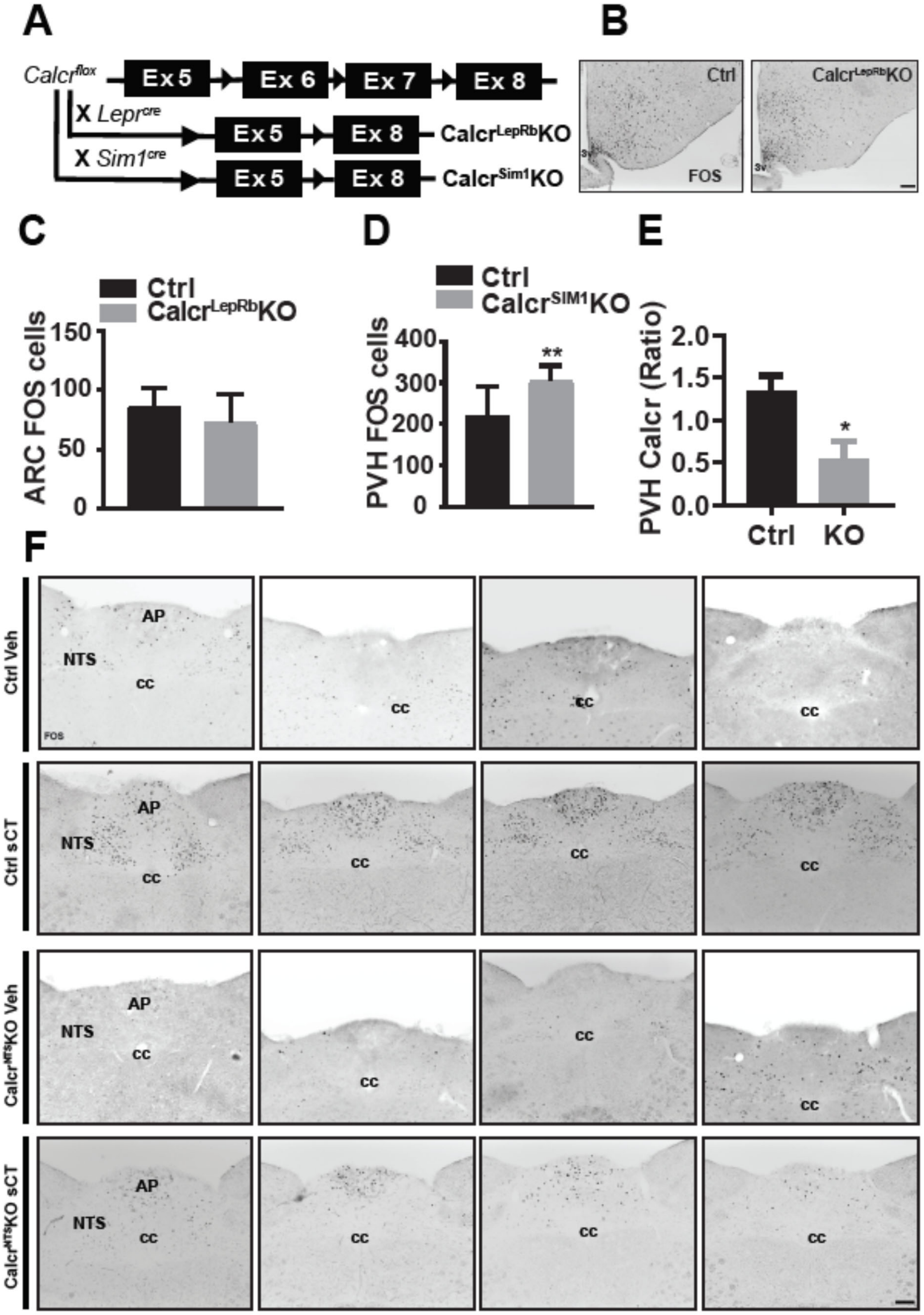

**Supplemental Figure 1 (Related to Figure 1): FOS response to sCT in *Calcr* knock-out lines. (A)** Schematic diagram showing the cross of $Calcr^{flox}$ with $Lepr^{cre}$ and $Sim1^{cre}$ mice to generate $\mathrm{Calcr}^{\mathrm{LepRb}}$KO and $\mathrm{Calcr}^{\mathrm{Sim1}}$KO mice. **(B)** Representative images showing FOS–IR in the hypothalamus of control (Ctrl) and $\mathrm{Calcr}^{\mathrm{LepRb}}$KO mice following treatment with sCT (150 μg/kg, IP). Scale bar equals 150 μm; 3V- third cerebral ventricle. **(C)** Quantification of ARC FOS-IR cells 2 hours after sCT (150 μg/kg) injection in $\mathrm{Calcr}^{\mathrm{LepRb}}$KO (n=5) and control (Ctrl, n=6) mice. (**D**) Quantification of PVH FOS-IR cells 2 hours after sCT injection (100 μg/kg, IP) in Ctrl (n=7) and $\mathrm{Calcr}^{\mathrm{SIM1}}$KO (n=9) mice. (**E**) Quantification of Calcr mRNA by qRT-PCR in PVH microdissections from Ctrl (n=6) and $\mathrm{Calcr}^{\mathrm{sim1}}$KO (n=7) mice. C, D, E: Mean +/- SEM is shown; *p<0.05, **p<0.01 by unpaired t-test. **(F)**. *Ad lib* control and $\mathrm{Calcr}^{\mathrm{NTS}}$KO mice were treated with vehicle (Veh) or sCT (150 μg/kg, IP) and were perfused for immunostaining for FOS two hours later. Sections from four representative mice are shown for each genotype/condition. cc- central canal. Scale bar equals 150 μm.

**Supplemental Figure 2**

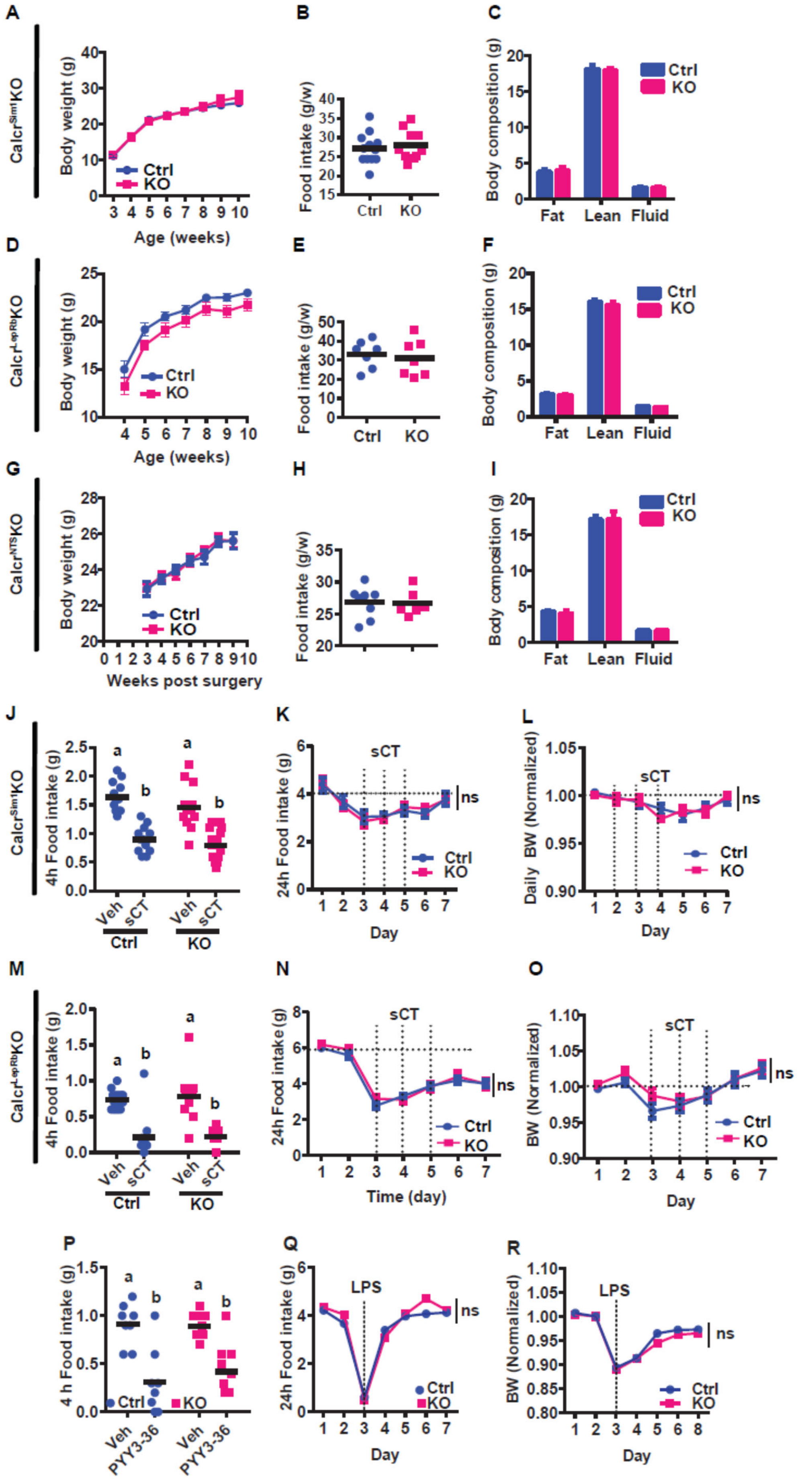

**Supplemental Figure 2 (Related to Figure 1): Energy balance and response to anorectic agents in CalcrKO mouse lines. (A-C)** Control and $Calcr^{Sim1}KO$ mice were monitored until 10 weeks of age for body weight **(A),** food intake **(B),** and body composition (14-18 weeks of age) **(C)**; n=4-10 per group. (**D-F**) Control and $Calcr^{LepRb}KO$ mice were monitored until 10 weeks of age for body weight **(D),** food intake **(E),** and body composition (10 weeks of age) **(F)**; n=6-13 per group. (**G-I**) Control and $Calcr^{NTS}KO$ mice were monitored for 7 weeks following surgery for body weight **(G)** and food intake **(H).** Body composition was determined nine weeks post-surgery **(I)**; n=6-8 per group. One-way ANOVA, Tukey's multiple comparisons was performed in A, D and G; Two-way ANOVA, Sidak's multiple comparisons test was performed in B, C, E, F, H, and I. All comparisons, p=not significant (ns). Responses to sCT (**J-O**): Food intake for the first 4h of the dark cycle (**J, M**) and daily food intake (**K, N**) and body weight (change from baseline) (**L, O**) during chronic treatment with IP vehicle (Veh) or sCT twice per day in $Calcr^{Sim1}KO$ (100 μg/kg sCT BID) (J-L) and $Calcr^{LepRb}KO$ (150 μg/kg sCT BID) (M-O). Dotted vertical lines denote days of sCT treatment; horizontal dotted lines denote baseline values. Individual replicates plus mean is shown for (J, M). Mean +/- SEM is shown for all other panels; n=13-17 per group in (J-L), n=9-15 per group in (M-O). One-way ANOVA, Tukey's multiple comparisons was performed in J and M, different letters indicate differences $p<0.05$ for the indicated groups; Two-way ANOVA, Sidak's multiple comparisons test was performed in K, L, N and O. ns: not significant. Effects of PYY3-36 and LPS on food intake and body weight in $Calcr^{NTS}KO$ mice. **(P-R) (P)** Control (Ctrl, Blue) and $Calcr^{NTS}KO$ (KO, Red) mice were treated with PYY3-36 (560 μg/kg, IP) and food intake was measured for 4 hours at the onset of the dark cycle; n=8 per group. **(Q, R)** Control (Ctrl, Blue) and $Calcr^{NTS}KO$ (KO, Red) mice were treated with LPS (300 μg/kg) and food intake and body weight were measured for subsequent days; n=8-10 per group. Mean +/- SEM is shown. One-way ANOVA, Tukey's multiple comparisons in P, different letters indicate difference, $p<0.05$. Two-way ANOVA, Sidak's multiple comparisons test was performed in Q and R. $^{**}p<0.01$, $^{***}p<0.001$, $^{****}p<0.0001$, ns: not significant.

Supplemental Figure 3

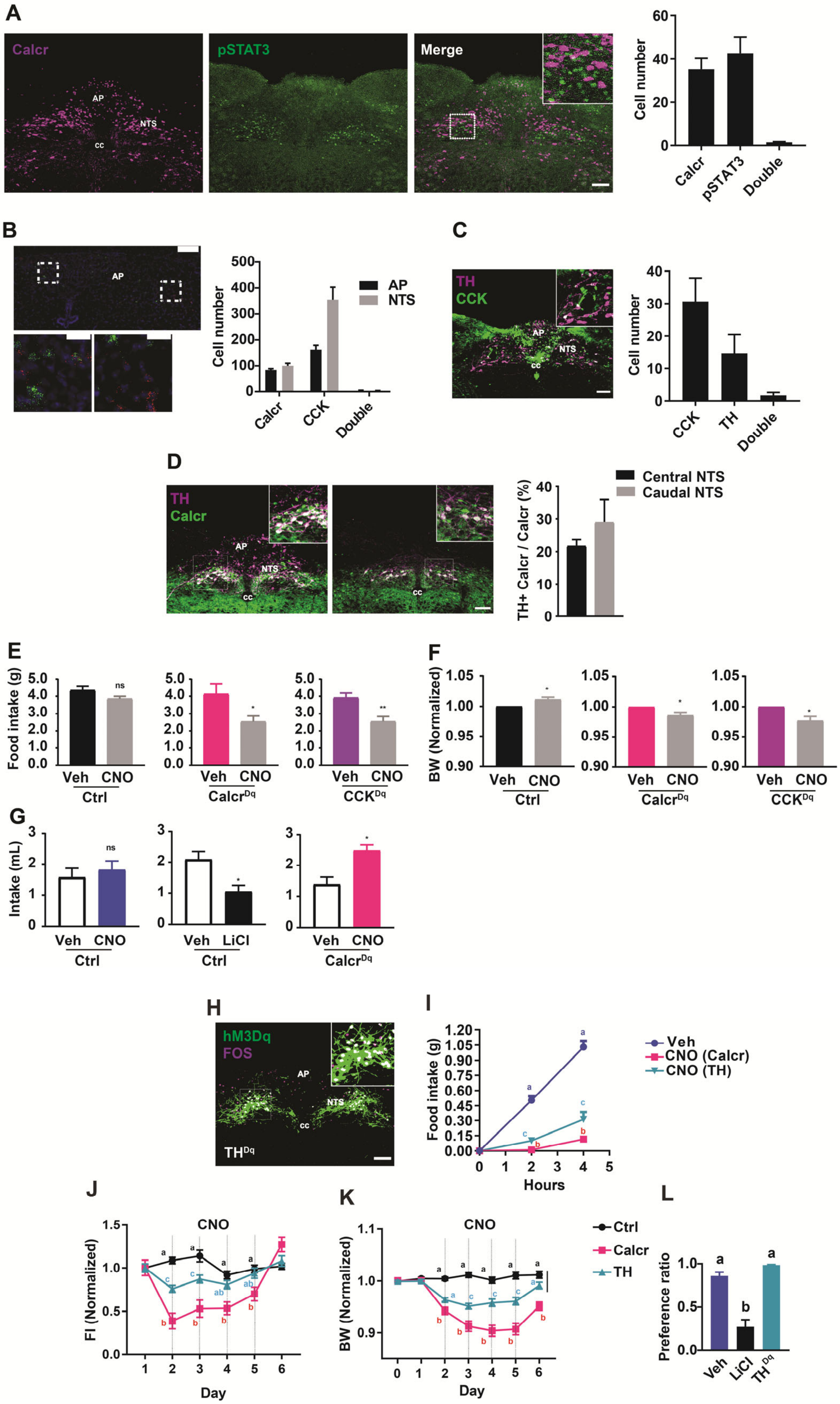

**Supplemental Figure 3 (Related to Figure 2): Calcr$^{NTS}$ neurons overlap with TH, but not CCK or LepRb neurons, and food intake following activation of Calcr$^{NTS}$, TH$^{NTS}$, or CCK$^{NTS}$ neurons. (A)** Representative images of the central NTS stained for pSTAT3 (green) and tdTomato (purple) in *Calcr$^{cre}$;tdTomato* reporter mice treated with Leptin (5 mg/kg, IP, 2 hours). Calcr positive, pSTAT3 positive and double positive cell number were counted unilaterally in the central NTS. Mean +/- SEM is shown; n=3. cc- central canal; scale bar equals 150 μm. **(B)** Representative stitched image for RNAScope fluorescent ISH analysis of *Cre* (red) and *Calcr* (green) in the NTS of *Cck$^{cre}$* mice; blue is DAPI. Scale bar = 100 μm in large image, 25 μm in insets; cc- central canal. Cells positive for *Calcr*, *Cre*, or both were quantified in the AP and NTS. Mean +/- SEM is shown; n=3. (**C**) Representative image of the central and caudal NTS stained for TH (purple) and tdTomato (green) in *Calcr$^{cre}$;tdTomato* reporter mice. Scale bar equals 150 μm; cc- central canal. Percent of *Calcr* (tdTomato) cells also containing TH-IR was quantified for each NTS region. Mean +/- SEM is shown; n=3. (**D**) Representative image of the central NTS stained for TH (purple) and tdTomato (green) in *CCKcre;tdTomato* reporter mice, scale bar eques 150 μm. Cells positive for *CCK*, *TH*, or both were quantified in the central NTS unilaterally. Mean +/- SEM is shown; n=3. **(E, F)** 24-hour response to a single dose of vehicle (Veh) or CNO (0.3 mg/kg, IP) in a crossover design at the onset of the dark cycle in control (Ctrl), Calcr$^{Dq}$, and CCK$^{Dq}$ mice. Food intake was determined after 24 hours (E) and body weight was determined at baseline and after 24 hours (F). Mean +/- SEM is shown; n=6 in control group, n=6 in calcr group, n=7 in cck group. **(G)** Intake data for Figure 2G: Control (Ctrl; left and middle panel) or Calcr$^{Dq}$ mice (right panel) were treated on alternating days with vehicle (Veh) paired with one randomly assigned flavor or stimulus (CNO (1 mg/kg) or LiCl (126 mg/kg)) paired with a different flavor for three conditioning sessions each. Consumption was measured for each flavor on the first post conditioning day. Mean +/- SEM is shown; n=11 in ctrl-CNO, n=12 in LiCl, n=8 in Calcr-Dq group. Paired t-test (E, F) or unpaired t-test (G), *p<0.05, **p<0.01, ns- not significant. **(H)** Representative images showing FOS (purple) and hM3Dq (green) 2 hours after CNO injection (1 mg/kg, IP) in TH$^{NTS}$-Dq (TH$^{Dq}$) mice. Scale bar equals 150 μm; cc- central canal. **(I)** Food (chow) intake during the first 4 hours of the dark cycle following treatment of Th$^{Dq}$ mice with vehicle (Veh; n=23-25) and CNO (1 mg/kg, IP, n=8) compared with food intake in CNO-treated Calcr$^{Dq}$ mice from Figure 2 (n=18-20). (**J, K**) Daily food intake (J) and body weight (K) were measured during 3 days of vehicle, 4 days of CNO (IP, 1 mg/kg, BID), and 2 additional days of vehicle injection for control (Ctrl; n=14) and TH$^{Dq}$ mice (n=8), along with the Calcr$^{Dq}$ mice plus Calcr$^{Dq}$ mice from Figure 2 (n=13). **(L)** CTA was also determined by pairing CNO (1mg/kg, IP) injected TH$^{Dq}$ mice with novel HFD (n=8 per group). Mean +/- SEM is shown. Two-way ANOVA, Sidak's multiple comparisons test was performed in I, J, and K; one-way ANOVA, Tukey's multiple comparisons was performed in L. Different letters indicate differences p<0.05.

**Supplemental Figure 4**

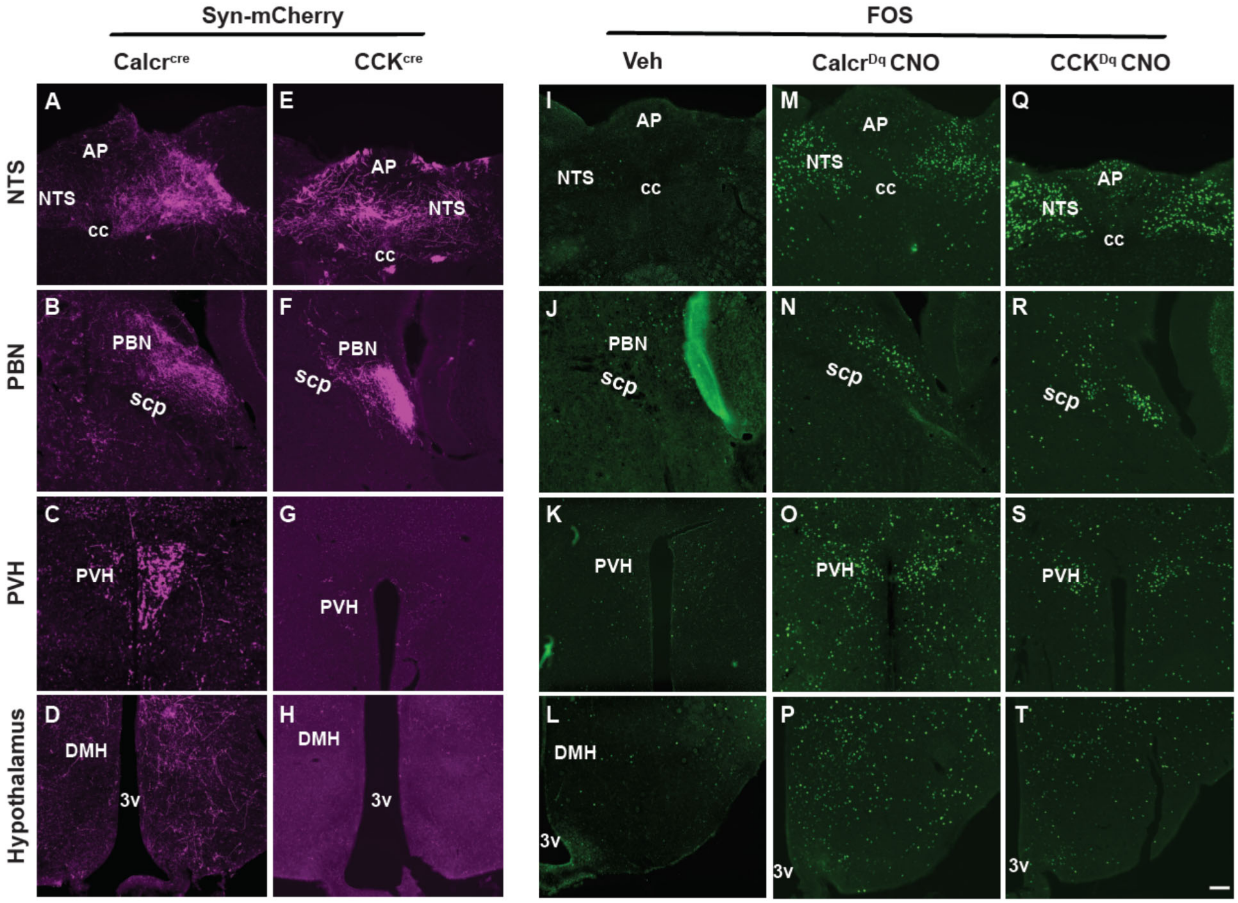


**Supplemental Figure 4 (Related to Figure 5): Downstream targets of Calcr$^{NTS}$ and CCK$^{NTS}$ neurons.** Following the injection of AAV$^{Flex\text{-}Syn\text{-}mCherry}$ into the NTS of Calcr$^{cre}$ **(A-D)** or CCK$^{cre}$ **(E-H)** mice, brains were sectioned and stained for mCherry (Purple). Representative images of major projection targets are shown. (**I-T**) Representative images showing FOS-IR (green) in major projection targets of Calcr$^{NTS}$ neurons in Calcr$^{NTS}$-Dq **(M-P)** and CCK$^{NTS}$-Dq **(Q-T)** mice following injection with vehicle (Veh) **(I-L)** or CNO (0.8 mg/kg, IP, AM) (**M-T**). Scale bar equals 150 μm; cc= central canal, scp=superior cerebellar peduncle, 3V- third cerebral ventricle. PBN=parabrachial nucleus, PVH=paraventricular hypothalamus. DMH=Dorsal medial hypothalamus. Images are representative of 3 per group.

Supplemental Figure 5

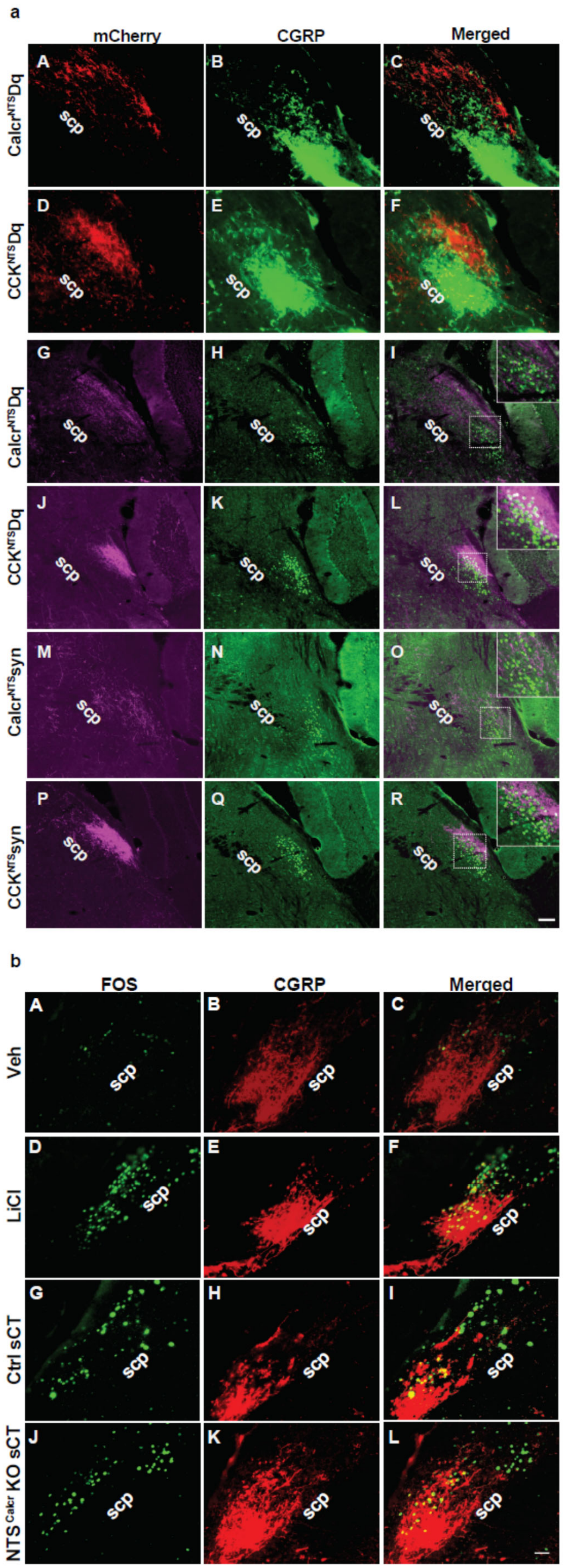

**Supplemental Figure 5 (Related to Figure 5): CCK$^{NTS}$ neurons, but not Calcr$^{NTS}$ neurons, project to the CGRP-containing PBN region (a) and Colocalization of sCT-induced FOS with CGRP-IR in the PBN (b). (a: A-F)** Representative images of hM3Dq (mCherry, red, left panels), CGRP (green, middle panels) and merged images (right panel) in the PBN of Calcr$^{NTS}$-Dq (A-C) and CCK$^{NTS}$-Dq mice (D-F). (**a: G-L**) Representative images of hM3Dq (mCherry, purple, left panels), GFP (green, middle panels) and merged images (right panel) in the PBN of Calcr$^{NTS}$-Dq/*Calca$^{cre\text{-}GFP}$* (G-I) and CCK$^{NTS}$-Dq/*Calca$^{cre\text{-}GFP}$* mice (J-L). Insets show digital zooms of the boxed area. (**a: M-R**) Representative images of Syn-mCherry (purple, left panels), GFP (green, middle panels) and merged images (right panel) in the PBN of *Calcr$^{cre}$/Calca$^{cre\text{-}GFP}$* (M-O) and *Cck$^{cre}$/Calca$^{cre\text{-}GFP}$* mice (P-R) following intra-NTS injection with Ad-Syn-mCherry. Insets show digital zooms of the boxed area. Images are representative of 3 per group. Scale bar equals 150 µm; scp=superior cerebellar peduncle. **(b: A-L)** Shown are representative images of FOS (green, left panels), CGRP (red, middle panels), and merged images (right) from mice treated with vehicle (Veh, **b: A-C**), LiCl (**b: D-F**), or with sCT (150 µg/kg,**b: G-L**) in control (Ctrl, A-I) or Calcr$^{NTS}$KO mice (**b: J-L**); representative of n=3 per group. Scale bar equals 75 µm; scp=superior cerebellar peduncle.

## Supplemental Figure 6

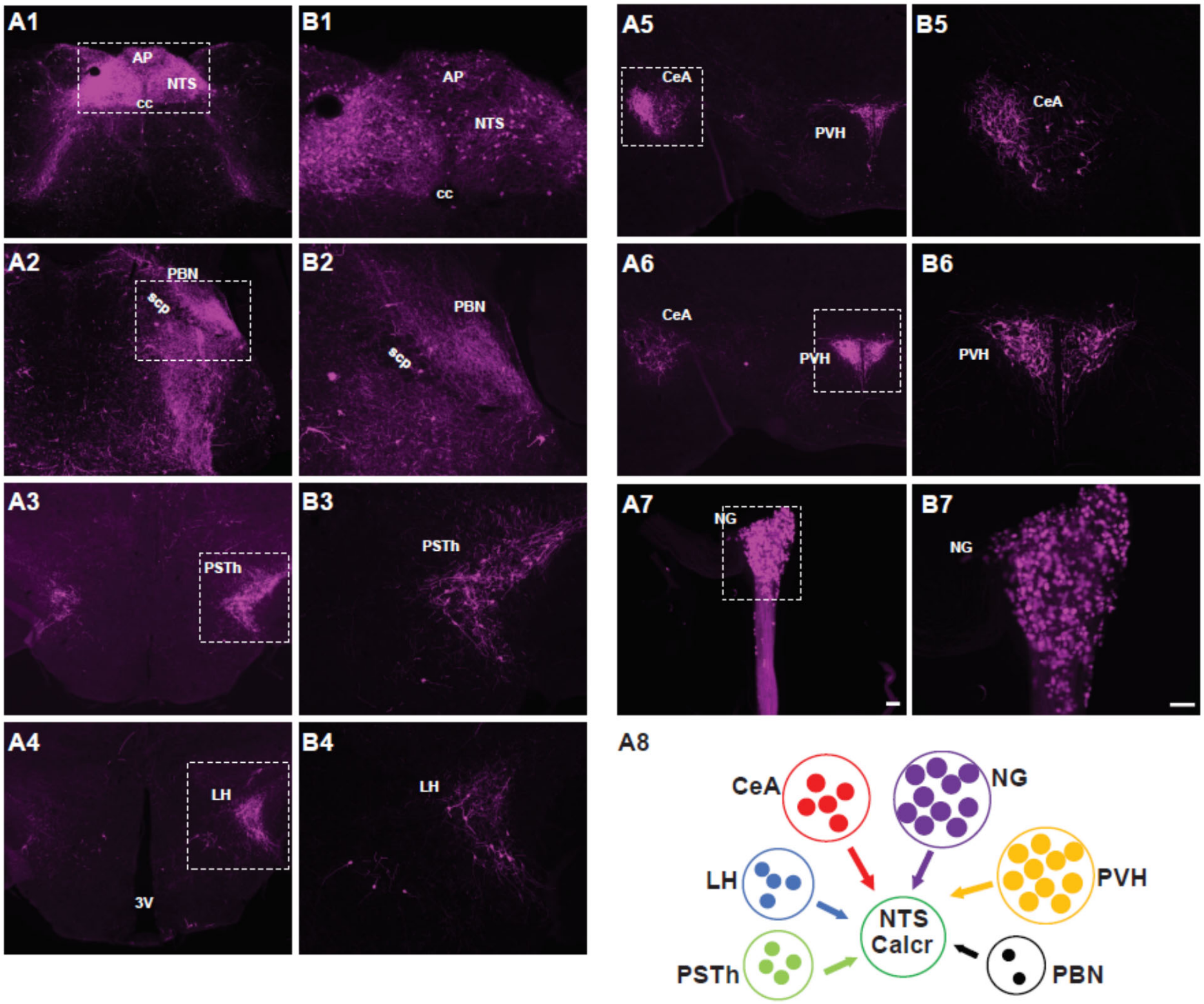


**Supplemental Figure 6 (Related to Figure 5): Single-synapse rabies tracing reveals inputs to Calcr$^{NTS}$ neurons.** AAV$^{Flex\text{-}TVA+G}$ was injected into the NTS of *Calcr$^{cre}$* mice; 1 month later the mice were injected with pseudotyped defective mCherry-expressing rabies virus. Brains were collected one week later and sectioned to identify mCherry-expressing afferents. Representative images of areas with substantial staining are shown. Images in A1-A7 were taken at 4x; B1-B7 show 10x images of the boxed regions. A8 shows a summary about the inputs that Calcr$^{NTS}$ neurons received throughout the nervous system, with number of dots proportional to the number of rabies-infected neurons detected in each region. Scale bars equal 150 µm. cc- central canal, PBN- parabrachial nucleus, scp=superior cerebellar peduncle, PSTh-parasubthalamic nucleus, LH-lateral hypothalamus, CeA- The central nucleus of the amygdala, PVH-para ventricular hypothalamus.NG- nodose ganglion. 3V- third cerebral ventricle. Images are representative of at least 3 animals.

## Supplemental Figure 7

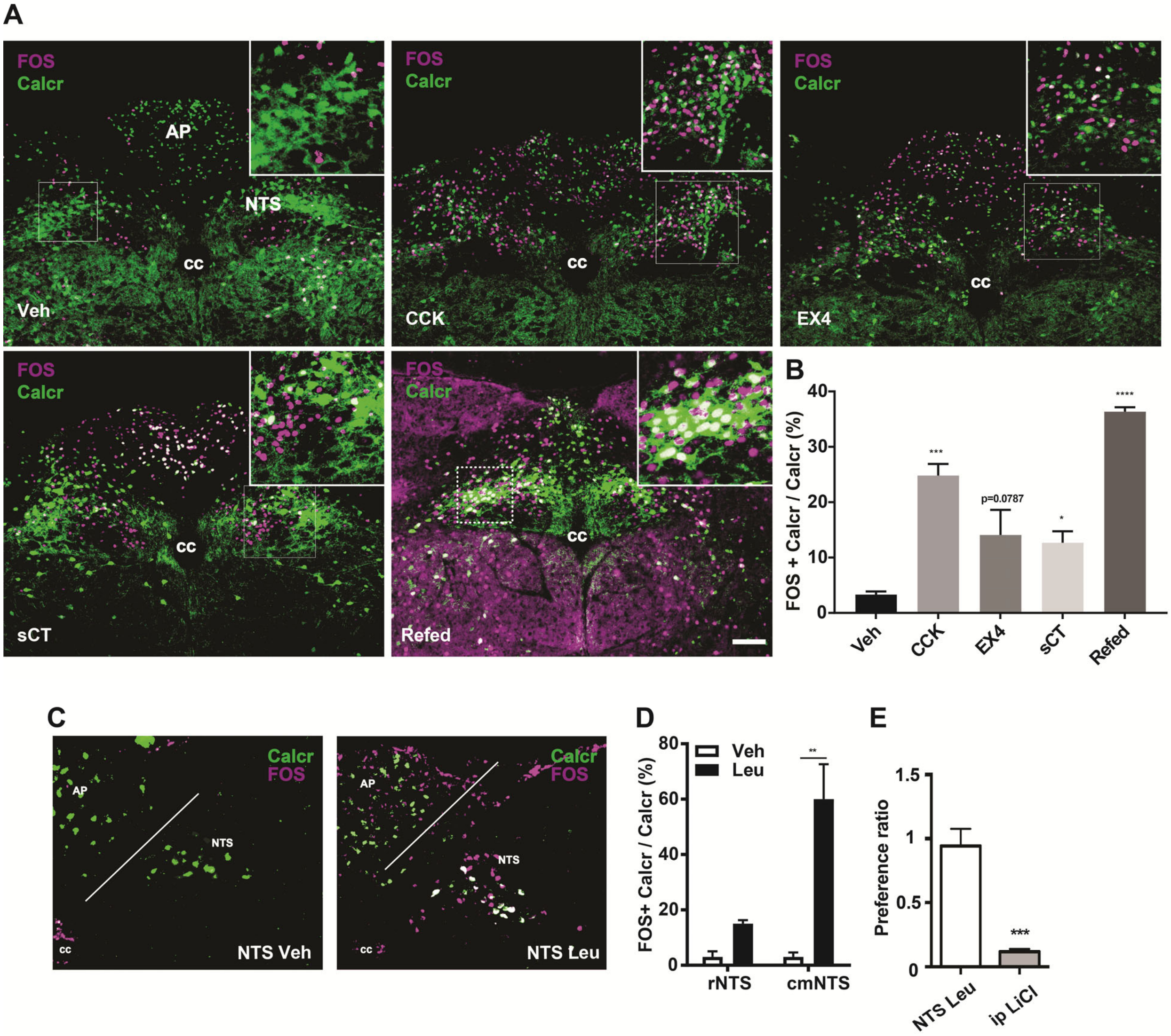


**Supplemental Figure 7 (Related to Figure 6): Activation of Calcr$^{NTS}$ neurons by gut peptide-mimetics and feeding, and NTS Leu activates Calcr$^{NTS}$ cells non-aversively. (A)** Representative NTS images from Calcr$^{cre}$;tdTomato mice showing FOS (purple) and Calcr neurons (mCherry, green) two hours after injection with vehicle (Veh), CCK (IP, 100 μg/kg), EX4 (IP, 150 μg/kg), sCT (IP, 150 μg/kg), or refeeding with normal chow for 2 hours after an overnight fast, as indicated. cc- central canal. Scale bar equals 150 μm. (**B**) Calcr$^{NTS}$ and FOS+Calcr$^{NTS}$ neurons were counted and plotted as percent of total in the lower right panel. Mean +/- SEM is shown, n=3 per group. Mean +/- SEM is shown, unpaired t test was performed to compare each treatment group with Veh, *p<0.05, ***p<0.001, ****p<0.0001, or p value as noted**.** Images are representative of 3 per group; cc- central canal. Scale bar equals 150 μm. **(C)** Representative images the NTS in *Calcr$^{cre}$* mice stained for FOS (purple) and Calcr (green) following treatment with NTS vehicle (Veh, left panel) or NTS Leu (right panel). White line shows demarcation between AP and NTS. n=4 in each condition. (**D**)

Quantification of FOS-containing NTS Calcr cells from the rostral NTS (rNTS) and central medial NTS (cmNTS) from images such as shown in (C), n=4 in each group. (**E**) Quantification of CTA produced by NTS Leu I.C.V injection paired with saccharin, LiCl IP injected mice paired with saccharin were positive controls (n=6 in each group). Shown is mean +/-SEM. Unpaired t-test: **p<0.01, ***p<0.001.
